# *HK2* knockdown is associated with context-dependent inflammatory and angiogenesis-related transcriptional programmes in glioblastoma cells

**DOI:** 10.3389/fimmu.2026.1842240

**Published:** 2026-06-16

**Authors:** Christopher Edwards, Taylor Richards, Hafid Omar Al-Hassi, Surya Prakash Shivakumar, Benjamin Small, Kate Butcher, Tracy Warr, Mark Morris, Timothy Dawson, Michael D. Jenkinson, Katherine Karakoula

**Affiliations:** 1 University of Wolverhampton, Faculty of Science and Engineering, Wolverhampton, United Kingdom; 2 Lancashire Teaching Hospital NHS Foundation Trust, Preston, United Kingdom; 3 Institute of Systems, Molecular and Integrative Biology, University of Liverpool, Liverpool, United Kingdom; 4 Department of Neurosurgery, The Walton Centre NHS Foundation Trust, Liverpool, United Kingdom

**Keywords:** glioblastoma, hexokinase, inflammation, angiogenesis, cytokine, chemokine

## Abstract

**Introduction:**

Deregulation of cellular metabolism is a hallmark of cancer, in which tumour cells upregulate glycolysis and preferentially ferment glucose to lactate in the ‘Warburg effect’. Tumour-specific upregulation of hexokinase 2 (*HK2*), which catalyses the first rate-limiting step of glycolysis, is a key driver of this phenotype and represents a promising therapeutic target in glioblastoma. Here, we sought to identify HK2-associated tumour processes that could serve as therapeutic co-targets by profiling the transcriptomic consequences of *HK2* knockdown (HK2KD) in model established glioblastoma cell lines and patient-derived short-term cell cultures.

**Methods:**

Glioblastoma cell lines and patient-derived short-term cell cultures were subjected to HK2KD using *HK2*-targeted siRNA. Glycolytic activity was assessed using the Seahorse Glycolytic Stress Test, and transcriptomic changes were analysed by RNA sequencing.

**Results:**

HK2KD was associated with reduced glycolytic activity in high-*HK2*-expressing models as well as extensive transcriptional remodelling. Integrative analysis using RNA sequencing, gene ontology and pathway enrichment, protein-protein interaction networks, and transcriptome-based perturbation matching indicated coordinated modulation of inflammatory, immune-associated and angiogenesis-linked gene programmes, including cytokine, chemokine and pro-angiogenic signalling axes. Notably, inflammatory pathway responses diverged markedly between independent glioblastoma models, representing a central finding of this study and highlighting context-dependent immunometabolic transcriptional changes following HK2KD.

**Discussion:**

These findings suggest that HK2KD is associated with transcriptional changes extending beyond glycolytic pathways, linking metabolic activity to immunological and angiogenic transcriptional networks in a model-dependent manner. Although functional effects on the tumour microenvironment were not directly assessed, this study provides a transcriptomic framework for understanding how *HK2* targeting may be associated with changes in tumour-associated signalling pathways, with potential implications for stratified metabolic and combination therapeutic strategies in glioblastoma.

## 1 Introduction

Glioblastoma is the most common type of adult brain tumour. Despite an aggressive treatment regimen (surgery, radiotherapy, concomitant and adjuvant chemotherapy), the prognosis of this cancer remains extremely poor, with a median patient survival of only ~15 months and fewer than 5% surviving 5 years (1). The dismal response rate can be attributed to the inevitable local recurrence of these tumours, which is driven by their characteristic infiltrative growth, therapeutic resistance, and heterogeneity. With current treatment for glioblastoma largely ineffective and associated with many side effects (2), there is a desperate need for new therapies that can target different glioblastoma genetic subpopulations with minimal negative effects on normal healthy cells.

Targeting of abnormal metabolic processes unique to glioblastoma has emerged as a potential strategy to meet this need (3, 4). The primary energy source for the cell, glucose, is initially broken down anaerobically into two molecules of pyruvate through a series of 10 cytosolic reactions known as glycolysis, which together net a gain of two ATP. Under aerobic conditions, pyruvate is subsequently transported into the mitochondrial matrix, where it is oxidised to acetyl coenzyme A (Acetyl-CoA) which then feeds into the tricarboxylic acid cycle (TCA) to yield proton donors (NADH, FADH_2_) for the oxidative phosphorylation (OxPhos) electron transport chain (ETC) that ultimately generates approximately 36 total ATP/glucose via ATP Synthase. When oxygen (which acts as the final electron acceptor in the ETC) is limiting, most differentiated cells redirect pyruvate generated by glycolysis away from mitochondrial OxPhos via fermentation to lactate. This lactate is then excreted as waste from the cell through H^+^ linked monocarboxylate transporters (MCTs).

Cancer cells, on the other hand, are paradoxically acknowledged to ferment pyruvate to lactate even in the presence of adequate oxygen as part of the ‘Warburg effect’. This metabolic phenotype necessitates significantly higher glucose consumption and glycolytic activity, with glycolytic rates up to 200 times higher than those observed in normal cells (5). Why Warburg-metabolising cancer cells appear to counterintuitively emphasise ‘aerobic glycolysis’ as a less efficient means of generating ATP from glucose than OxPhos remains to be fully determined. One prevailing theory is that aerobic glycolysis, which can occur 10–100 times faster than OxPhos, provides an accelerated source of ATP to cancer cells that can be fine-tuned in accord with the fluctuating nutrient availability of the tumour microenvironment (6–8). The excreted Warburg product, lactate, is also associated with immunosuppression in the tumour microenvironment (9–12), highlighting the potential for metabolic-targeted agents to enhance the effectiveness of immunotherapies against glioblastoma.

The role of hexokinases, which catalyse the phosphorylation of glucose to glucose-6-phosphate in the first rate-limiting committed step of glycolysis, has generated particular interest in the field of tumour Warburg metabolism. Four highly homologous hexokinase isoforms, encoded by separate genes, have been characterised in mammalian tissue: HK1, HK2, HK3, and HK4. Whilst sharing common biochemical properties, each isoform displays significant variation in regard to their regulation and tissue distribution (13). For instance, *HK1* is ubiquitously expressed as the ‘housekeeping isoform’, while *HK2* represents a more regulated form whose expression is limited to a select few adult tissues such as the skeletal and cardiac muscle and adipose tissue (14). HK2 is also recognised to uniquely possess active catalytic sites within both its N-terminal and C-terminal domains, while HK1 solely relies upon its C-terminal domain for enzymatic function (15, 16). With each of these catalytic domains acknowledged to possess distinct kinetic properties, HK2 has been postulated to retain its activity under conditions that are inhibitory to the single catalytic domain of HK1 such as hypoxia (17).

Studies have shown *HK2* to be abundantly and selectively overexpressed in a range of cancer types arising in tissues that normally exclusively express *HK1* (18, 19). This is particularly evident in glioblastoma, where increased *HK2* expression confers poorer patient prognosis (19–22). Evidence indicates that this differential expression is governed by epigenetic silencing of *HK2* in normal brain tissue, with hypomethylation of its promoter/intron 1 acting as the principal driver of amplified expression within glioblastoma (21, 23, 24). Given its role in catalysing the first committed step of glycolysis, the upregulation of HK2 is considered a key driver of the cancer Warburg phenotype that ultimately supports rapid production of ATP in both normoxic and hypoxic conditions. Overexpression of *HK1* has failed to rescue aerobic glycolysis in *HK2-*silenced glioblastoma cells, further implicating HK2 as the key driver of Warburg metabolism in this tumour (20). Moreover, *HK2* silencing has also been found to decrease glioblastoma tumour growth, invasion, angiogenesis, and resistance to therapy (20, 25–27).

Given its significance to tumour-specific Warburg metabolism and limited expression in normal brain, HK2 arguably represents the principal target to orient the design of glioblastoma-selective metabolic therapies. To support this approach, we aimed in this study to identify novel HK2-associated tumour processes that could serve as co-targets as part of future metabolic therapeutic strategies to treat glioblastoma. Through performing RNA sequencing (RNAseq) following HK2KD, we found the *HK2* expression of glioblastoma cell cultures to be associated with inflammatory, immune and angiogenesis-related gene signatures, suggesting that HK2 is associated with transcriptional programmes relevant to pathways targeted by immuno− and anti−angiogenic therapies.

## 2 Materials and methods

### 2.1 Ethics approval

This study was conducted under local ethical approval granted by the Life Science Ethics Committee, University of Wolverhampton: Cell biology and genetic investigations of primary and metastatic brain tumours (LSEC/201920/KK/150). Tumour material was obtained with associated ethical approval from the Brain Tumour North West (1012/1604_1) biobank and the Liverpool Neuroscience Biobank at The Walton Centre (North Wales Rec: 20/WA/0043). Written informed consent was obtained from all patients prior to their donation of tumour tissue for research at the primary centre for surgical treatment.

### 2.2 Cell culture

Human glioblastoma patient-derived short-term cell cultures and established cell lines were grown in ‘growth medium’ comprised of HAM’s F10 nutrient mixture with 25 mM HEPES and 1 mM glutamine (Gibco) supplemented with 10% FBS (Gibco). Cell cultures were incubated under normoxic conditions at 37°C in a standard non-CO_2_ incubator. Growth medium replacement or passaging was performed every 2–3 days in accordance with cell confluency. University of Wolverhampton (UWLV) and Institute of Neurology (IN) patient-derived short-term cell cultures were derived from fresh glioblastoma tumour biopsy material as previously described (28, 29). The established glioblastoma cell lines U87-MG and U251-MG were supplied by Professor Darrell Bigner (Duke University, USA).

### 2.3 Transfection

Glioblastoma cell cultures were seeded in 6 well plates at a quantity of 3.5 x10^5^ cells/well. 24 hours after plating, cells were transfected with either HK2 Ambion Silencer Select (s6562, Ambion Life Technologies) or non-targeting Silencer Select Negative Control No. 1 siRNA (Ambion Life Technologies) at a final concentration of 25 pmol. Transfection was performed with Opti-MEM serum-free medium (Gibco) and Lipofectamine 3000 (Invitrogen). Cells were utilised for experimental investigation 48 hours after transfection.

### 2.4 RNA extraction and RT-qPCR analysis

Total RNA was isolated from glioblastoma cell cultures using a RNeasy mini kit (Qiagen) according to the manufacturer’s instructions. cDNA was synthesised from extracted total RNA using a High-Capacity cDNA Reverse Transcription Kit (Applied biosystems) following the manufacturer’s instructions. Target gene expression was investigated by performing quantitative RT-PCR using TaqMan™ Gene Expression Assays (FAM) (*HK2*-Hs00606086_m1, *GAPDH*-Hs02786624_g1) (Applied biosystems) and TaqMan Gene Expression Master Mix (Applied biosystems) upon a 7500 Fast Real Time PCR System as per the manufacturer’s instructions. All reactions were performed in triplicate with non-template negative controls. Relative gene expression fold difference values were calculated via the comparative Ct (2-ΔΔCT) method, with target gene expression normalised to the housekeeping gene *GAPDH*. RNA derived from adult normal human brain tissue (Total RNA, Human Adult Normal, 5 Donor Pool, Brain, Temporal Lobe, BioGenomics, 299045) was used as a healthy normal tissue control for experimental comparisons to glioblastoma cell cultures.

### 2.5 Extracellular flux analysis of cellular glycolytic activity

The glycolytic activity of glioblastoma cell cultures was determined by measuring changes in their extracellular acidification rate (ECAR) during a Seahorse XFp Glycolytic Stress Test assay on an Agilent Seahorse XFp Analyzer (Agilent/Seahorse Bioscience, Santa Clara, CA). One day prior to assay, cell cultures were seeded at 3x10^4^ cells/well in growth medium on Seahorse XFp cell culture miniplates (Agilent/Seahorse Bioscience) coated with gelatin (25 µL/well for 30 minutes) and incubated overnight at 37 °C in a non-CO_2_ incubator. Two wells (well A, well H) of each cell culture miniplate utilised per assay were alternatively loaded with sterile PBS to act as a background control. To ensure the correct functioning of the Seahorse XFp sensor cartridge during the assay, each well of the cartridge was hydrated in Seahorse XF Calibrant (200 µL/well) and incubated overnight at 37 °C in a non-CO_2_ incubator until required for use.

On the day of the assay, plated cells were washed by removing all but 20 μL of growth medium from each well and adding 180 µL prewarmed (37 °C) ‘assay medium’ comprised of Glycolytic Stress Test Agilent Seahorse XF DMEM Base Assay Medium supplemented with 2 mM glutamine (Agilent/Seahorse Bioscience). Washing was performed twice. During the final wash, 180 μL of medium was removed from each well and replaced with 160 μL of fresh assay medium, bringing the final well volume to 180 μL.

Following medium replacement, cells were incubated at 37 °C for 45 minutes in a non-CO_2_ incubator to equilibrate prior to use in the assay. During this time, the Seahorse assay testing compounds were prepared and loaded into the injection port of the XFp sensor cartridge. Each test compound from the Glycolytic Stress Test assay kit (103346-100, Agilent/Seahorse Bioscience) was resuspended in assay medium to produce a 10x stock concentration before loading into the sensor cartridge at a specified volume as per the manufacturer’s instructions. The final Glycolytic Stress Test assay compound concentrations were 10 mM Glucose (20 µL, Port A), 1 µM Oligomycin (22 µL, Port B), and 50 mM 2-DG (25 µL, Port C).

To run the assay, the default glycolytic stress test template programme was selected upon the XFp Analyzer, and the compound-loaded sensor cartridge was inserted into the machine. After an initial 30-minute calibration period, the sensor cartridge utility plate was discarded and replaced with the cell plate, commencing the assay. All assays were run at 37 °C. Data from each assay were analysed using Seahorse Wave software.

### 2.6 RNA sequencing

RNAseq was performed on biological replicates (n = 2) of the established glioblastoma cell line U87-MG and the patient-derived short-term cell cultures IN859 and IN2045. Cells were transfected with either *HK2*-targeting siRNA or non-targeting negative control siRNA, alongside matched non-transfected wild-type controls as per 2.3 and 2.4. Transfection and sequencing were performed in single respective batches. RNASeq was performed by Biomarker Technologies (BMKGENE, https://www.bmkgene.com/). Purity, concentration, and integrity of submitted RNA (1µg/sample) were verified by BMKGENE prior to library construction. Library preparation was performed using a NEBNext Ultra™ RNA Library Prep Kit for Illumina (New England Biolabs) following manufacturer’s recommendations. Prepared libraries were sequenced on an Illumina NovaSeq X (with PE150 mode) and paired-end reads were generated. Raw sequencing data (raw reads) from each sample were processed by BMKGENE in-house to produce clean data. Clean reads were mapped to the Ensembl 95 human GRCh38 reference genome using HISAT2 (30), with StringTie (31) employed to assemble mapped reads into transcripts. Reads that align to each gene were then counted and normalised by gene length using FPKM (Fragments per kilobase of exon model per million reads mapped) by StringTie. Pearson correlation coefficient R was then applied to evaluate reproducibility between biological replicates of samples prior to differential gene expression analysis. Principal component analysis (PCA) was also performed on the FPKM of each sample.

Differentially expressed genes (DEGs) between sample groups were identified using DESeq2 (32) based on raw, unnormalised gene-level read counts. The threshold to detect a significant DEG was set at Fold Change ≥ ± 1.5 (Log_2_ Fold Change ±0.58) and False Discovery Rate (FDR) adjusted p value ≤ 0.05. Differential expression of 5 select DEGs reported by DESeq2 was validated by RT-qPCR as per 2.4.

Interactions between proteins encoded by DEGs were identified in-house using the Search Tool for the Retrieval of Interacting Genes/Proteins (STRING) (http://string-db.org; version: 12.0) online database (33). Protein-protein interactions (PPIs) were predicted based on the evidence parameters ‘Experiments’ and ‘Databases’, with PPIs with at least medium confidence (interaction score > 0.4) amalgamated into PPI networks by STRING. The recommended Markov Cluster Algorithm (MCL) was employed in STRING to identify clusters of functionally related proteins (i.e. interacting proteins with a higher STRING global score) within PPI networks, using a default inflation parameter of 3.0 (34). Clusters defined by STRING ≥ 5 PPIs were noted. Top 10 ‘hub’ DEG-encoded proteins with the highest interaction count (19, 35) were identified using node degree rank upon STRING.

Functional enrichment (FDR-adjusted p value ≤ 0.05) of DEGs for Gene Ontology (GO) Biological Process (BP) (36, 37) and the Kyoto Encyclopaedia of Genes and Genomes (KEGG) (38) pathway gene sets was also performed in-house using GOseq (39) on the bioinformatics platform Galaxy (Usegalaxy.eu) (40). GOseq was run with default settings using built-in GO annotations for the human genome (GRCh38) and a gene length file (output from a Stringtie read counts file), with Wallenius’s noncentral hypergeometric distribution method used to adjust for potential gene length bias. Upon detection of a significantly enriched GO BP, a z-score was calculated to characterise whether the process was more likely to be downregulated (negative value) or upregulated (positive value) by the expression direction of their comprising DEGs (41). To facilitate data interpretation, enriched GO BPs were further compiled into a functionally related clustered network using Enrichment map (42) upon Cytoscape (43). BP nodes were only connected (by edges) if their gene content overlapped by more than 25% (42). An inbuilt automated layout algorithm (autoannotate) placed BPs with high DEG overlap (i.e., highly similar) into descriptively labelled clusters of terms describing related pathways and processes. Maps of enriched KEGG pathways with annotated participating DEGs were generated using Pathview (44) on Galaxy.

Drug perturbation gene set enrichment analysis (dpGSEA) of differential gene expression data was also performed using the Connectivity Map (CMap) database (45). DEGs were ranked prior to dpGSEA by Sign (FC)*-log_10_ (FDR). dpGSEA was conducted using ‘fgsea’ in Galaxy, using the ranked list of DEGs and a CMap database GMT file (obtained from Drug Signature Database, https://dsigdb.tanlab.org/DSigDBv1.0/) as inputs. The number of permutations was set at 100000. An FDR-adjusted p value of ≤ 0.25 was utilised as a cutoff for identification of CMap compound-induced expression profiles that were enriched for DEGs, consistent with standard GSEA-based exploratory analyses.

## 3 Results

### 3.1 Glioblastoma cell cultures recapitulate patient tumour overexpression of *HK2*

Patient-derived short-term cell cultures are widely recognised to maintain the genetic and phenotypic characteristics of their parental original tumour tissue, while also recapitulating its heterogeneity (29, 46). With their superior clinical relevance in mind, a large number (14) of glioblastoma patient-derived short-term (grown up to a maximum passage of 15) cell cultures were utilised to model glioblastoma in this study, alongside the established glioblastoma cell lines U87-MG and U251-MG. To identify novel HK2-associated tumour processes, it was first necessary to verify that our model glioblastoma cell cultures maintained the characteristic upregulation of *HK2* expression seen in patient tumours. To achieve this, the level of *HK2* mRNA in each model cell culture was measured and compared to that of control human adult normal brain tissue by RT-qPCR.

**Figure 1:**
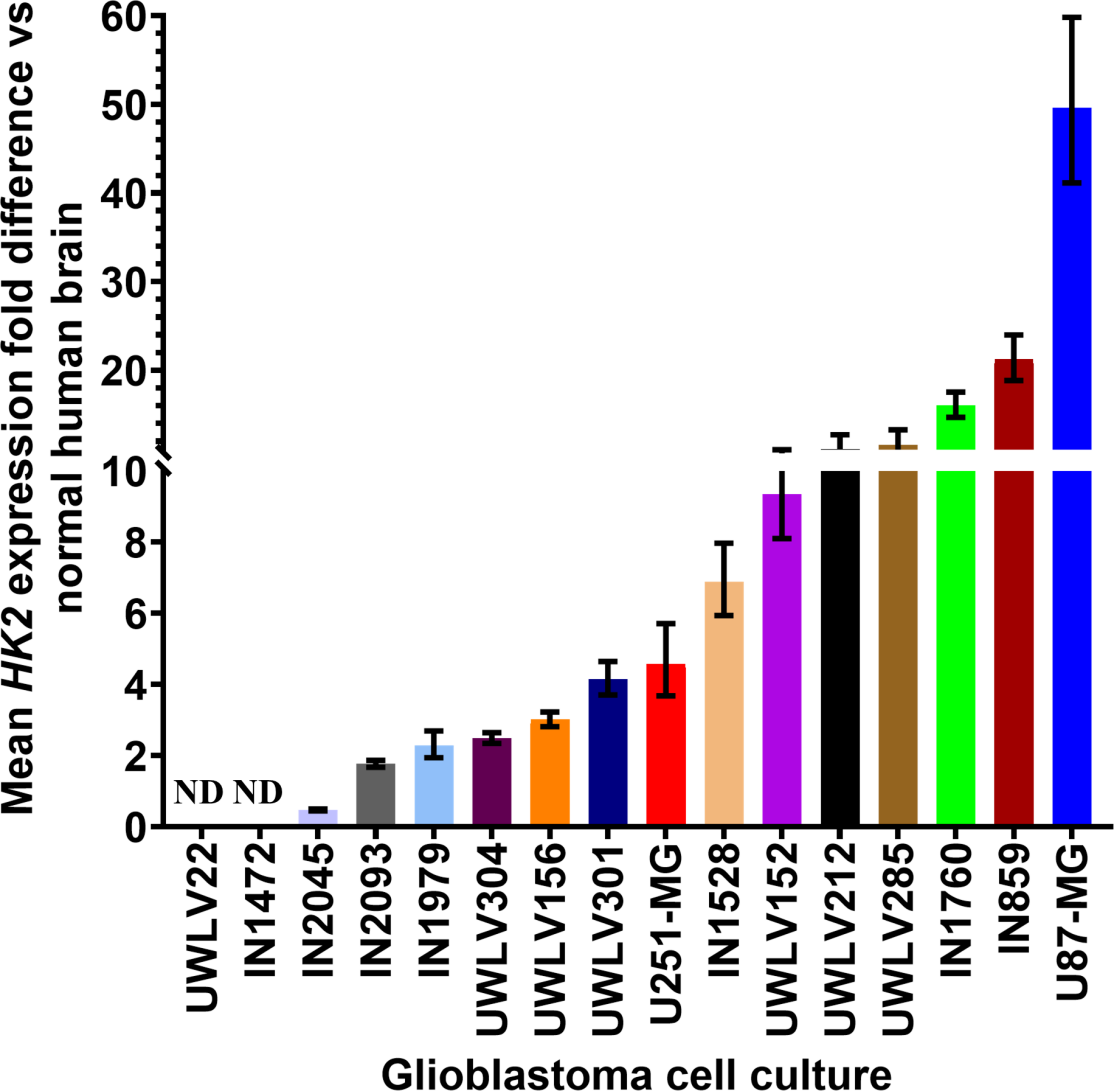
Glioblastoma cell cultures retain upregulation of *HK2* seen in patient tumours. The relative fold difference between the *HK2* expression of established glioblastoma cell lines and patient-derived short-term cell cultures against control human adult normal brain tissue (2-ΔΔCT) was determined by RT-qPCR. *GAPDH* represented the housekeeping control gene employed in this analysis. Bar heights indicate the mean *HK2* expression fold difference vs normal human brain of each cell culture, ascertained from the average of three technical replicate assays. Error bars depict the upper (FDmax) and lower (FDmin) possible limits of mean fold difference in relation to the standard deviation between the ΔCTs of each culture’s technical replicates. ND, not detectable, due to Ct>35.

Increased *HK2* mRNA was detected in 87.5% (14/16) of the cell cultures (**Figure 1**, **Table 1**). This elevated *HK2* expression ranged from a 1.77- to 46.97-fold difference compared to control normal brain tissue, which was normalised to a 1-fold level (**Figure 1**, **Table 1**). The established cell line U87-MG (46.97-fold) and the patient-derived short-term cell culture IN859 (21.26-fold) displayed the highest *HK2* upregulation (**Figure 1**, **Table 1**). In contrast, the patient-derived short-term cell cultures IN2045 (0.47-fold), UWLV22 (no expression), and IN1472 (no expression) showed either downregulation or no expression of *HK2* (**Figure 1**, **Table 1**). This data suggested that the glioblastoma cell cultures used in this study can maintain the elevated expression of *HK2* seen in patient tumours, thereby indicating their suitability as models for investigating HK2-driven glycolysis in this cancer. Conversely, the cell cultures with decreased expression of *HK2* (IN2045, UWLV22, IN1472) may depend on alternative metabolic mechanisms for energy production rather than HK2-driven glycolysis.

**Table 1:** *HK2* expression fold difference values in glioblastoma cell cultures relative to normal human brain control.

Glioblastoma cell culture	Mean HK2 expression fold difference vs normal human brain (FDmin-max)
U87-MG	49.60 (41.12-59.81)
IN859	21.26 (18.84-23.98)
IN1760	16.05 (14.69-17.54)
UWLV285	11.60 (10.14-13.27)
UWLV212	11.12 (9.71-12.73)
UWLV152	9.34 (8.10-11.04)
IN1528	6.88 (5.94-7.97)
U251-MG	4.58 (3.68-5.71)
UWLV301	4.15 (3.70-4.65)
UWLV156	3.01 (2.81-3.23)
UWLV304	2.50 (2.35-2.64)
IN1979	2.28 (1.94-2.70)
IN2093	1.77 (1.67-1.87)
IN2045	0.47 (0.45-0.50)
UWLV22	ND
IN1472	ND

ND, not detectable.

### 3.2 Inhibition of *HK2* expression dampens the glycolytic activity of glioblastoma cell cultures

Next, we sought to validate whether this upregulation of *HK2* was associated with glycolytic activity in glioblastoma. To assess this, we employed the Seahorse XFp glycolytic stress test assay to evaluate the effect of *HK2* expression inhibition on live glycolytic activity. The seahorse XFp measures glycolytic activity through isolating a small volume (~2 µl) of medium above a seeded cell monolayer to create a ‘transient microchamber’ (47) whereby real-time changes in extracellular acidification rate (ECAR) can be recorded following the export of glycolytic waste lactate with H^+^ via MCTs (47, 48).

The established cell line U87-MG (49.60-fold *HK2*) and the patient-derived short-term cell culture IN859 (21.26-fold *HK2*), which exhibited the highest *HK2* expression, were selected for this experiment. The patient-derived short-term cell culture IN2045 (0.47-fold *HK2*) was also included as a low *HK2*-expressing comparator. *HK2* expression was knocked down by transfection with *HK2*-targeting siRNA, with transfection with non-targeting negative control siRNA separately performed to generate comparative negative controls. Efficient HK2KD in U87-MG, IN859, and IN2045 was confirmed via RT-qPCR (**Supplementary Figure 1A**).

**Figure 2:**
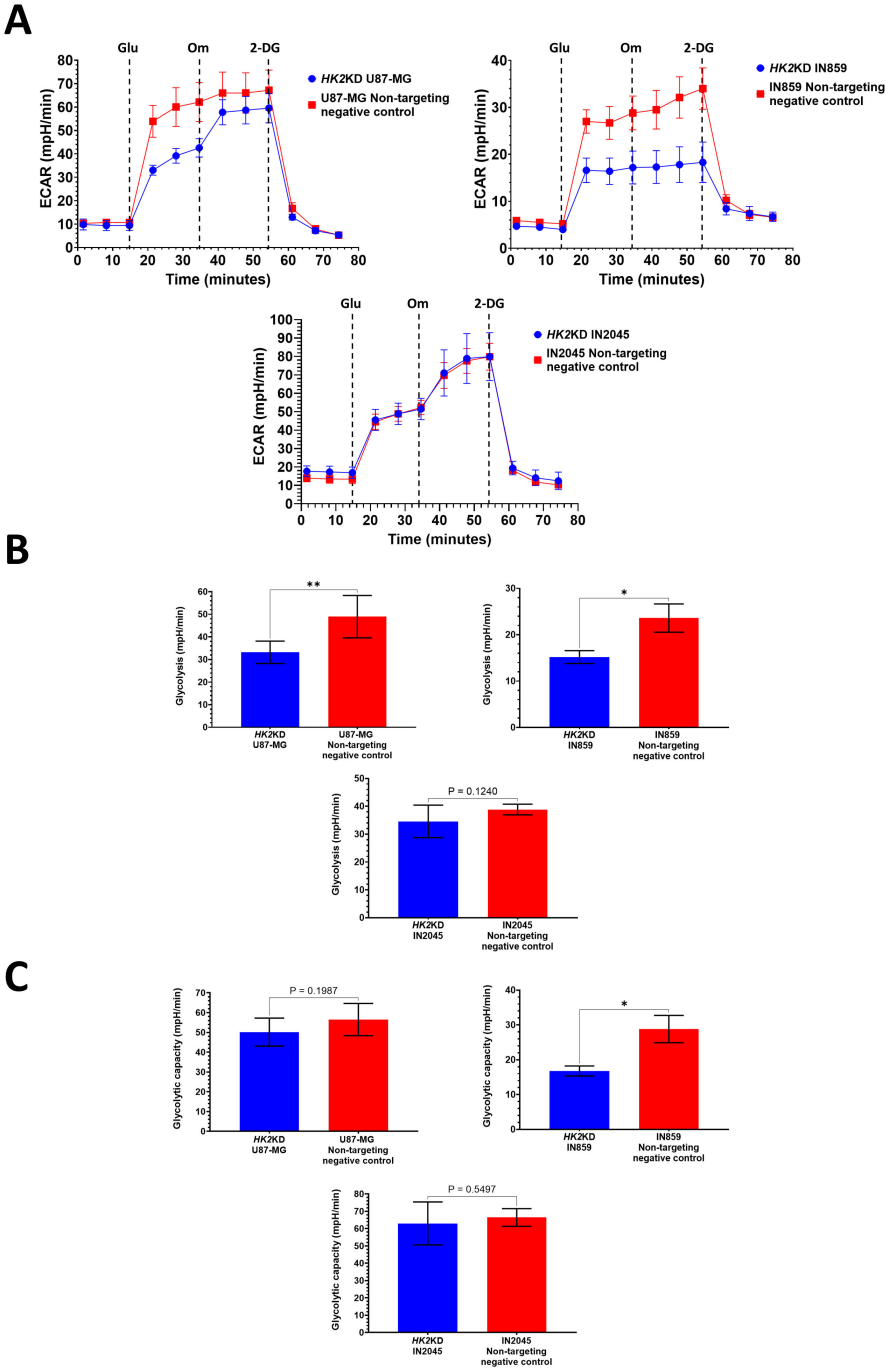
HK2KD selectively suppresses glycolytic flux in glioblastoma cell cultures with high *HK2* expression. **(A)** Extracellular acidification rate trace of HK2KD and non-targeting negative control transfected U87- MG, IN859, and IN2045 glioblastoma cells following sequential injection of glucose (20 mM), oligomycin (1 µM) and 2-Deoxy-D-glucose (100 mM) upon the Seahorse XFp Glycolytic Stress Test Assay. Basal glycolysis **(B)** and glycolytic capacity **(C)** of HK2KD and non-targeting negative control transfected glioblastoma cells following injection of glucose and oligomycin. Data values are presented as the mean ± standard deviation of two experiments, utilising separate biological replicates, performed in triplicate. Statistical comparisons were performed utilising non-paired t-test. Non-significant p values > 0.05 are indicated upon image, p ≤ 0.05=*, p ≤ 0.01=**.

To initiate the glycolytic stress test, a non-limiting concentration of glucose (10 mM) was first added to cells incubated in glucose-free, glutamine-supplemented assay medium. This led to an increase in ECAR, which subsequently represented the rate of cellular glycolysis under basal conditions (**Figure 2A**) (48). High *HK2* expressing U87-MG (p= 0.0045) and IN859 (p= 0.0386) cells that received HK2KD displayed significantly decreased levels of basal glycolysis in comparison to their negative control counterparts (**Figures 2A, B**). This indicated that *HK2* is associated with glycolytic activity in these cell cultures, although a considerable degree of basal glycolysis remained following HK2KD. HK2KD had no significant effect (p= 0.1240) upon the basal glycolysis of the low *HK2* expressing cell culture IN2045 (**Figures 2A, B**).

Oligomycin (1 µM), an ATP synthase inhibitor, was then added to the cells as the next part of the Glycolysis Stress Test assay. By inhibiting mitochondrial OxPhos, oligomycin induces cellular energy production to shift toward glycolysis as a sole remaining mode of generating ATP (48). As a result, any increase in ECAR following the addition of oligomycin thereby reflected the cellular maximum glycolytic capacity (**Figure 2A**). Similar to basal glycolysis, HK2KD IN859 (p= 0.0282) cells exhibited significantly reduced glycolytic capacity compared to controls (**Figures 2A, C**). The fellow high *HK2* expressor U87-MG (p= 0.1987), however, did not follow such a trend, with HK2KD having no significant impact on its glycolytic capacity (**Figures 2A, C**). Likewise, HK2KD did not significantly impact the glycolytic capacity of the low *HK2*-expressing cell culture IN2045 (p= 0.5497) (**Figures 2A, C**). Finally, the glucose analogue 2-deoxy-d-glucose (2-DG, 50 mM) was added to conclude the Glycolysis Stress Test assay (**Figure 2A**). Through simultaneously inhibiting hexokinases (competitive, non-competitive allosteric feedback) and phosphoglucose isomerase (PGI) (competitive) (48–50), 2-DG blocked glycolytic activity and thereby restored the ECAR of HK2KD and non-targeting negative control siRNA-transfected cells to baseline (**Figures 2A, C**).

Taken together, this data affirmed the importance of *HK2* toward driving elevated glycolytic activity in glioblastoma cell cultures that specifically upregulate its expression.

### 3.3 *HK2* expression is associated with inflammatory transcriptional programmes relevant to the glioblastoma tumour microenvironment

Interrogating the impact of *HK2* expression inhibition upon the glioblastoma transcriptome may provide hypothesis-generating insight into novel canonical/non-canonical relationships that could serve as future co-therapeutic targets.

To investigate the transcriptional consequences of HK2KD, RNAseq was performed on glioblastoma cell cultures transfected with either *HK2*-targeting siRNA or non-targeting negative control siRNA, alongside non-transfected wild-type (WT) cell cultures that were sequenced in parallel under identical experimental conditions to assess the transcriptional effects associated with siRNA transfection itself.

To facilitate identification of potential HK2-related genes and pathways, the highest *HK2*-expressing models, the established cell line U87-MG (49.60-fold *HK2*) and patient-derived short-term cell culture IN859 (21.26-fold *HK2*), were chosen for transfection and sequencing. To account for potential metabolic heterogeneity within patient tumours and act as a comparative control, the low *HK2* expressing patient-derived short-term cell culture IN2045 (0.47-fold *HK2*) was also analysed. HK2KD in these cell cultures was confirmed prior to sequencing via RT-qPCR (**Supplementary Figure 1B**).

High-quality RNAseq data were obtained, with 96.59-99.00% of clean reads mapping to the human genome (**Supplementary Table 1**). Biological replicates suggested strong concordance based on Pearson correlation analysis, with no notable deviation observed between replicates (**Supplementary Figure 2A**). Principal component analysis (PCA) of FPKM-normalised expression data further showed tight clustering of biological replicates and separation between experimental groups (**Supplementary Figure 2B**).

Differential gene expression (DEG) analysis (**Supplementary Table 2**) indicated widespread transcriptional differences between WT non-transfected samples and non-targeting negative control siRNA-transfected samples, suggesting that siRNA delivery and associated cellular stress significantly influenced global gene expression patterns. Consequently, downstream differential expression analyses were restricted to comparisons between *HK2*-targeting and non-targeting negative control siRNA conditions to isolate changes specifically associated with HK2KD.

Using this approach, we identified a broad set of DEGs associated with HK2KD. DEGs associated with HK2KD totalled 363 in U87-MG, 589 in IN859, and 231 in IN2045, respectively (**Supplementary Tables 3–5**). The direction of this differential expression varied across the HK2KD cell cultures, with HK2KD U87-MG (256/363, 70% of DEGs downregulated) and IN2045 (151/231, 65% of DEGs downregulated) skewed toward downregulation of gene expression, whereas HK2KD IN859 was marginally biased toward upregulation (325/589, 55% of DEGs upregulated).

Basally high *HK2*-expressing U87-MG and IN859 were found to share 50 common DEGs following HK2KD, the majority of which were downregulated (35/50, 70%) (**Supplementary Table 6**). An additional 35 genes were found to be differentially expressed in U87-MG, IN859, and IN2045 after HK2KD. These shared DEGs notably exhibited the same expression direction across the HK2KD cell cultures, with the majority downregulated (28/35, 80%) (**Supplementary Table 7**). To validate the RNAseq data, the expression of 5 selected DEGs (*FGF2*, *MET, PDHX*, *SDC4*, and *TGFBI*) common to all the HK2KD cell cultures was analysed by RT-qPCR. The expression direction for each of these reported DEGs was found to match that observed upon RT-qPCR, thereby validating the RNA seq-reported changes in gene expression (**Supplementary Table 8**).

To gain a deeper insight into the relationships among genes differentially expressed between the HK2KD cell cultures, the interactions of the proteins they encode were also examined using the STRING database, which generated a network featuring clusters of closely interacting proteins (≥ 5 PPIs). 20 of the 85 DEGs shared between HK2KD U87-MG and IN859 formed a STRING network with 14 total PPIs, with a single cluster of 5 PPIs orientated around the growth factor Fibroblast Growth Factor 2 (FGF2) (**Supplementary Figure 3A**). FGF2 interactors in this cluster included the FGF coreceptor SDC4, the growth factor TGFα, and the cytokine/growth receptor tyrosine kinases CSF1R and MET (**Supplementary Figure 3A**). FGF2 and its interactors were all downregulated across HK2KD U87-MG and IN859, except for MET. 6 of the 35 inputted DEGs shared between all the HK2KD cell cultures formed 4 total PPIs (**Supplementary Figure 3B**). These consisted of FGF2 with SDC4, CSF1R, MET and PAK2 with PSMC1 (**Supplementary Figure 3B**).

To further explore the processes and/or pathways associated with HK2KD-related transcriptional changes in glioblastoma, gene category enrichment analysis was performed on the entire cohort of DEGs found in each HK2KD cell culture with GO Biological Process (BP) and KEGG pathway gene sets. All enriched GO BPs were subsequently assigned a z-score indicating whether the process was more likely to be downregulated (negative value) or upregulated (positive value) based on the expression direction of their comprising DEGs (41). PPIs between DEG-encoded proteins from each HK2KD cell culture were also identified and constructed into a network using STRING.

117 GO BPs were found to be significantly enriched among the 363 DEGs associated with HK2KD in U87-MG (vs 12107 background non-DEGs). To facilitate data interpretation and minimise gene set redundancy, these HK2KD U87-MG enriched GO BPs were then graphically organised into a network using ‘enrichment map’, which grouped highly similar gene sets to generate a total of 14 functionally related clusters describing related pathways and cellular processes (42).

**Figure 3:**
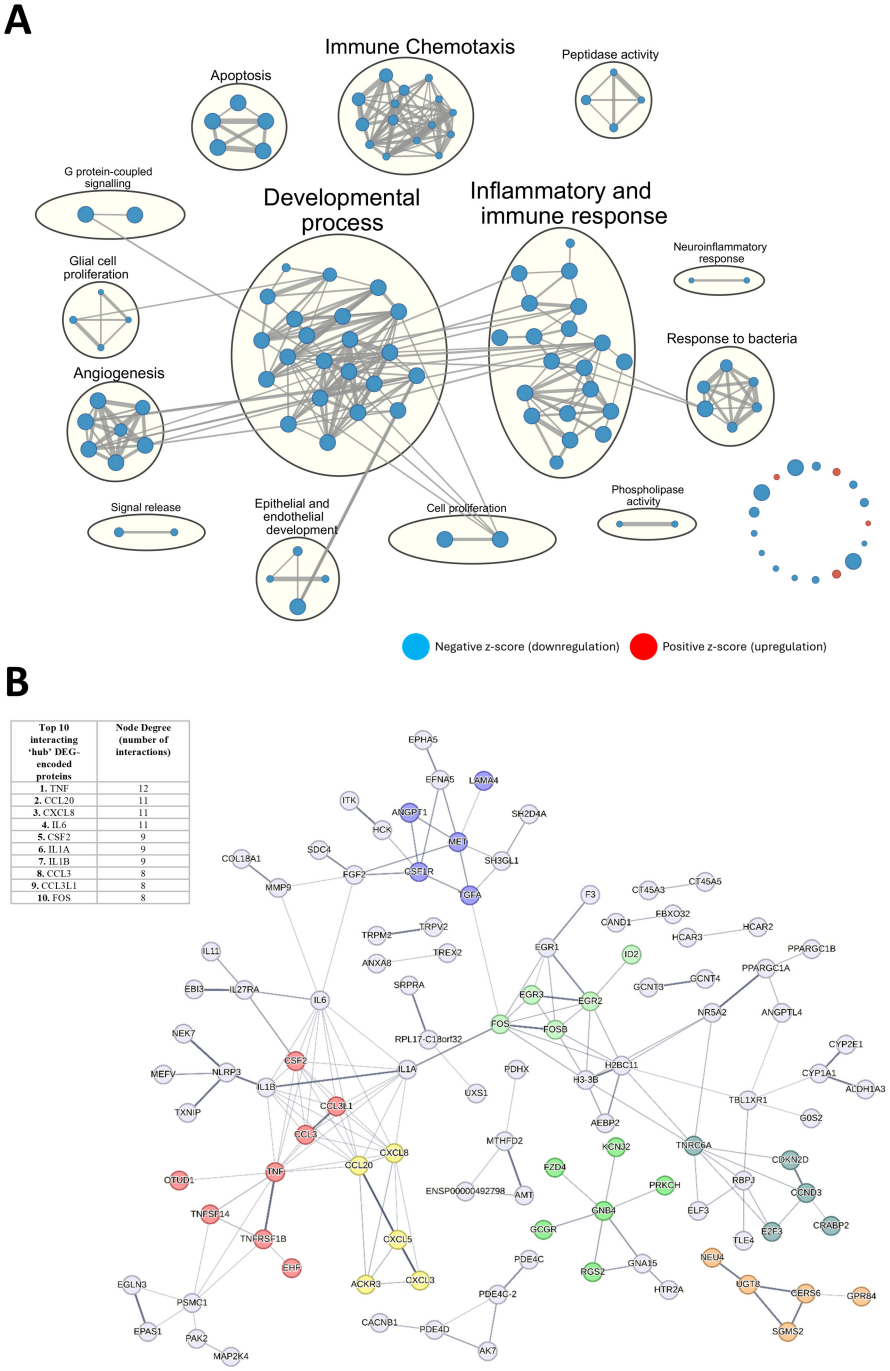
HK2KD U87-MG differentially expressed genes are associated with inflammation and angiogenesis. **(A)** Enrichment map of GO BPs enriched for HK2KD U87-MG differentially expressed genes (DEGs). Each node on the map represents an enriched GO BP, with node size corresponding to the number of DEGs comprising the enriched term. Nodes were coloured by their z-score, which was indicative of either down(blue) or up(red) regulation. Edges (shown in grey) connected nodes if their gene content overlapped by more than 25%. Edge thickness was proportional to the number of DEGs shared between nodes. Connected nodes were autonomously placed into clusters of functionally related pathways/processes using autoannotate. Cluster name size was proportional to the number of comprising connected BPs. Unconnected nodes were circularly organised. **(B)** STRING network of interactions between proteins encoded by HK2KD U87-MG DEGs. Potential protein-protein interactions (PPIs) were identified (by a medium confidence score of 0.4 or greater) upon STRING and assembled into a network. The thickness of lines indicates the strength of data support/degree of confidence supporting the predicted interaction between DEGs. Clusters of DEGs with a ‘high’ interaction score were identified using the STRING MCL algorithm. Clusters ≥ 5 PPIs are indicated by distinct colours. The top 10 ‘hub’ DEG-encoded proteins with the highest interaction counts (node degree) are listed in the embedded figure table and represent the principal drivers of connectivity within this STRING network.

All the BP clusters on the HK2KD U87-MG enrichment map had a negative z-score, which was indicative of their downregulation (**Figure 3A**). The map featured two particularly large clusters, one that was composed of various BPs related to developmental processes (22), and another that featured several BPs predominantly associated with inflammatory and/or immune response (20) (**Figure 3A**). Notable BPs within the inflammatory and/or immune response cluster included ‘immune system response’, ‘activation of innate immune response’, ‘inflammatory response’, and ‘cytokine signalling pathway’ (**Supplementary Figure 4**). Several distinct clusters of BPs associated with inflammation and immune response were also evident within the HK2KD U87-MG enrichment map. These included ‘response to bacteria’, which shared DEGs with the ‘inflammatory and/or immune response’ cluster, ‘immune cell chemotaxis’, and ‘neuroinflammatory response’ (**Figure 3A**; **Supplementary Figure 4**). The immune cell chemotaxis cluster featured a large array of BPs specifically linked to the recruitment and migration of immune cells, such as ‘mononuclear cell migration’, ‘neutrophil migration’, and ‘granulocyte chemotaxis’ (**Supplementary Figure 4**).

Several KEGG pathways, including the ‘cytokine-cytokine receptor interaction’ pathway and those associated with aberrant inflammatory and immune processes in rheumatoid arthritis and protozoan parasitic diseases, were also enriched in HK2KD U87-MG through overrepresentation of several downregulated DEG-encoded cytokines and chemokines (**Supplementary Figure 5**).

107 out of the 363 HK2KD U87-MG DEGs formed 165 total PPIs upon STRING, with 7 clusters ≥ 5 PPIs (**Figure 3B**; **Supplementary Table 12**). Consistent with the enrichment of BPs and KEGG pathways that signified HK2KD altered inflammatory signalling in U87-MG, the largest PPI cluster (shown in pink/red, n=8 DEG-encoded proteins) within the HK2KD U87-MG STRING network consisted mainly of cytokines (CSF2, TNF-α, TNFSF14) or chemokines (CCL3, CCL3L1) whose expression was downregulated. The downregulated receptor for TNF-α, TNFRSF1B, was also present in this cluster (**Figure 3B**). The downregulated trio of IL1α, IL1β, and IL6 also interacted within the network, with a cluster of interacting downregulated chemokines (CCL20, CXCL3, CXCL5, CXCL8) (shown in yellow) further evident (**Figure 3B**).

**Figure 4:**
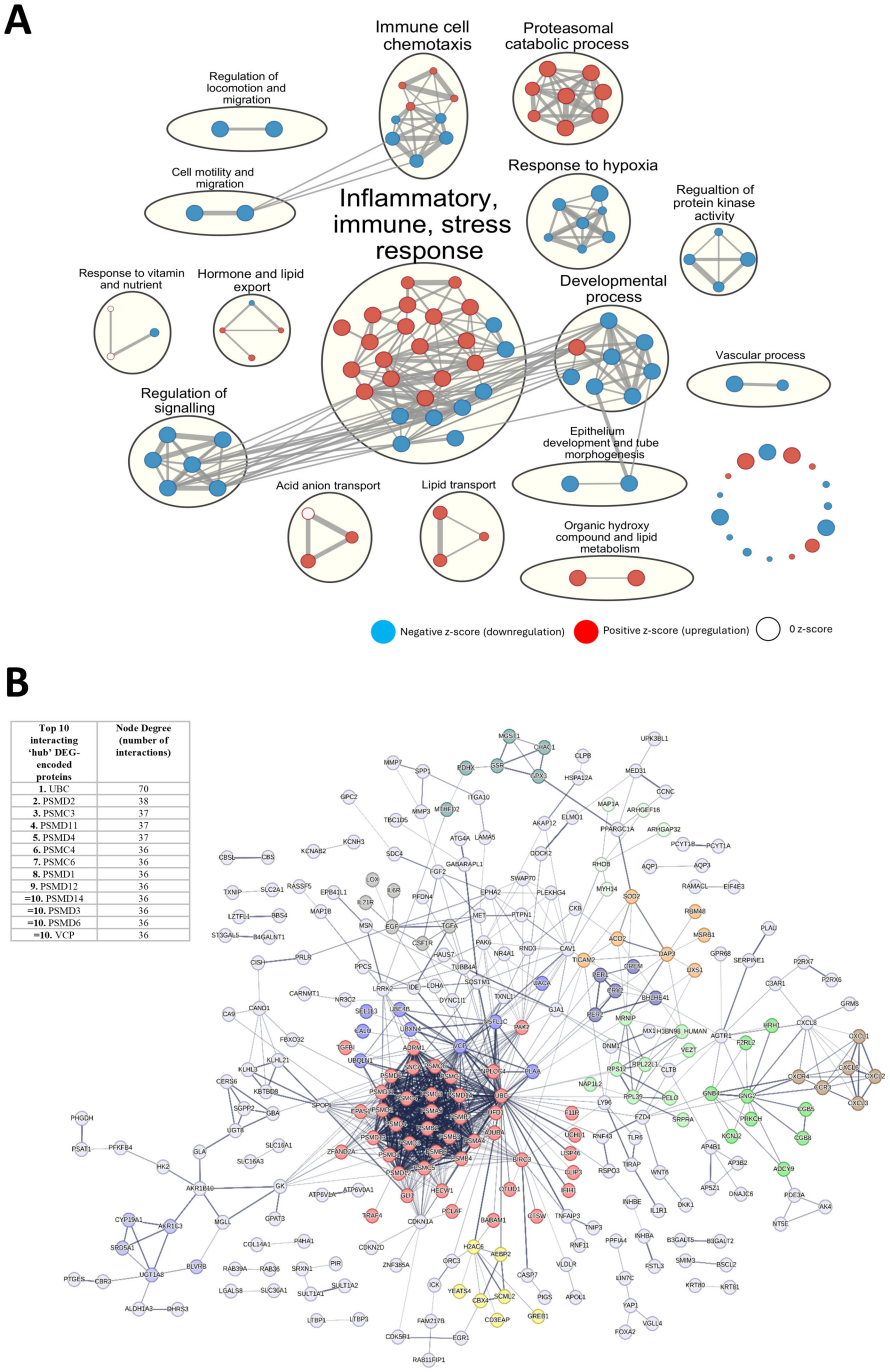
HK2KD IN859 differentially expressed genes are associated with inflammation and the proteasome. **(A)** Enrichment map of GO BPs enriched for HK2KD IN859 differentially expressed genes (DEGs). Each node on the map represents an enriched GO BP, with node size corresponding to the number of DEGs comprising the enriched term. Nodes were coloured by their z-score, which was indicative of either down(blue) or up(red) regulation. Nodes with a neutral (0) z-score are displayed in white. Edges (shown in grey) connected nodes if their gene content overlapped by more than 25%. Edge thickness was proportional to the number of DEGs shared between nodes. Connected nodes were autonomously placed into clusters of functionally related pathways/processes using autoannotate. Cluster name size was proportional to the number of connected BPs comprising it. Unconnected nodes were circularly organised. **(B)** STRING network of interactions between proteins encoded by HK2KD IN859 DEGs. Potential protein-protein interactions (PPIs) were identified (by a medium confidence score of 0.4 or greater) upon STRING and assembled into a network. The thickness of lines indicates the strength of data support/degree of confidence supporting the predicted interaction between DEGs. Clusters of DEGs with a ‘high’ interaction score were identified using the STRING MCL algorithm. Clusters ≥ 5 PPIs are indicated by distinct colours. The top 10 ‘hub’ DEG-encoded proteins with the highest interaction counts (node degree) are listed in the embedded figure table and represent the principal drivers of connectivity within this STRING network.

109 GO BPs were significantly enriched among the 589 DEGs associated with HK2KD in IN859 (vs 12222 background non-DEGs). Use of enrichment map then grouped these enriched BPs into 16 functionally related clusters (**Figure 4A**). The largest cluster (26 BPs) upon the HK2KD IN859 map was comprised of several processes related to inflammatory, immune, and stress response (**Figure 4A**), which was significant given that a similar large inflammatory and immune response associated cluster was also observable within the HK2KD U87-MG map (**Figure 3A**).

18/26 of the enriched BPs within the ‘inflammatory, immune and/or stress response’ cluster of HK2KD IN859 were characterised by a positive z-score, including ‘inflammatory response’, ‘defence response’ and ‘response to bacterium’ (**Supplementary Figure 6**), which interestingly all displayed opposing negative z-scores in HK2KD U87-MG (**Supplementary Figure 4**). In further similarity with HK2KD U87-MG, the HK2KD IN859 enrichment map also featured a substantial cluster of GO BPs associated with ‘immune cell chemotaxis’ (**Figures 3A**, **4A**; **Supplementary Figure 4**, **6**). The BPs of this cluster ‘chemotaxis’, ‘taxis’, ‘locomotion’, ‘cell chemotaxis’, and ‘myeloid cell migration’ were characterised by a negative z-score, whereas ‘leukocyte chemotaxis’, ‘(cellular) response to chemokine’, and ‘chemokine-mediated signalling pathway’ displayed a positive z-score (**Supplementary Figure 6**).

2 KEGG pathways were identified to be statistically enriched in HK2KD IN859: ‘proteasome’ and ‘cytokine-cytokine receptor interaction’. The enrichment for the proteasome KEGG pathway, and the upregulation of its composing DEGs, complemented the presence of a sizable cluster of proteasome-related BPs with positive z-scores on the HK2KD IN859 enrichment map (**Supplementary Figure 7A**; **Figure 4A**). Enrichment for the cytokine-cytokine receptor interaction KEGG pathway in HK2KD IN859 (**Supplementary Figure 7B**) was chiefly driven by the overrepresentation of various DEG-encoded chemokines that were contrastingly downregulated in HK2KD U87-MG.

Despite (1) their basal high *HK2* expression and (2) HK2KD being previously suggested to significantly lower their glycolytic activity, no BPs and KEGG pathways related to glycolysis or glucose metabolism were found to be enriched in HK2KD U87-MG or IN859.

281 out of the 589 HK2KD IN859 DEGs formed 943 total PPIs upon STRING, with 12 clusters ≥ 5 PPIs (**Figure 4B**; **Supplementary Table 13**). In line with the enrichment for the proteasome-related GO BPs and KEGG pathway, the largest PPI cluster (shown in pink/red, n= 44 DEG-encoded proteins) within the HK2KD IN859 STRING network was primarily composed of structural components forming the 26S proteasome (**Figure 4B**). These components included α- (PSMA4, PSMA5) and β-subunits (PSMB2, PSMB3, PSMB4, PSMB6, PSMB7) of the core 20S proteasome subcomplex, as well as ATPase (PSMC1, PSMC3, PSMC4, PSMC5, PSMC6) and non-ATPase subunits (PSMD1, PSMD2, PSMD3, PSMD4, PSMD6, PSMD11, PSMD12, PSMD13, PSMD14) of the regulatory 19S proteasome subcomplex. All of these proteasomal subunits were upregulated except for PSMC1, which was downregulated across all the HK2KD cell cultures (**Supplementary Table 7**). Other members of this cluster included ubiquitin C (UBC), and the modulators of endoplasmic reticulum-associated (proteasomal) degradation (ERAD), NPLOC4 and UFD1 (**Figure 4B**). Several other DEG-encoded proteins associated with ERAD, including the ATPase VCP and its cofactors UBQLN1, UBXN4, and UBE4B, also clustered in the network (shown in purple/blue) (**Figure 4B**). A cluster of DEG-encoded CXCL- type chemokines (CXCL1, CXCL2, CXCL3, CXCL6) and chemokine receptors (CCR3, CXCR4), which were all upregulated, were further notable in the network (shown in beige/light tan) (**Figure 4B**).

Just 2 GO BPs were significantly enriched among the 231 DEGs detected following KD in the low *HK2* expressing cell culture IN2045 (vs 12875 background non-DEGs). Of these enriched BPs, ‘cytoplasmic translation’ was characterised by a negative z-score, whereas ‘positive regulation of protein tyrosine kinase activity’ exhibited a positive z-score. A single KEGG pathway, ‘ribosome’, was found to be statistically enriched in HK2KD IN2045, corroborating the enrichment for the translation-related BP (**Supplementary Figure 8**).

**Figure 5:**
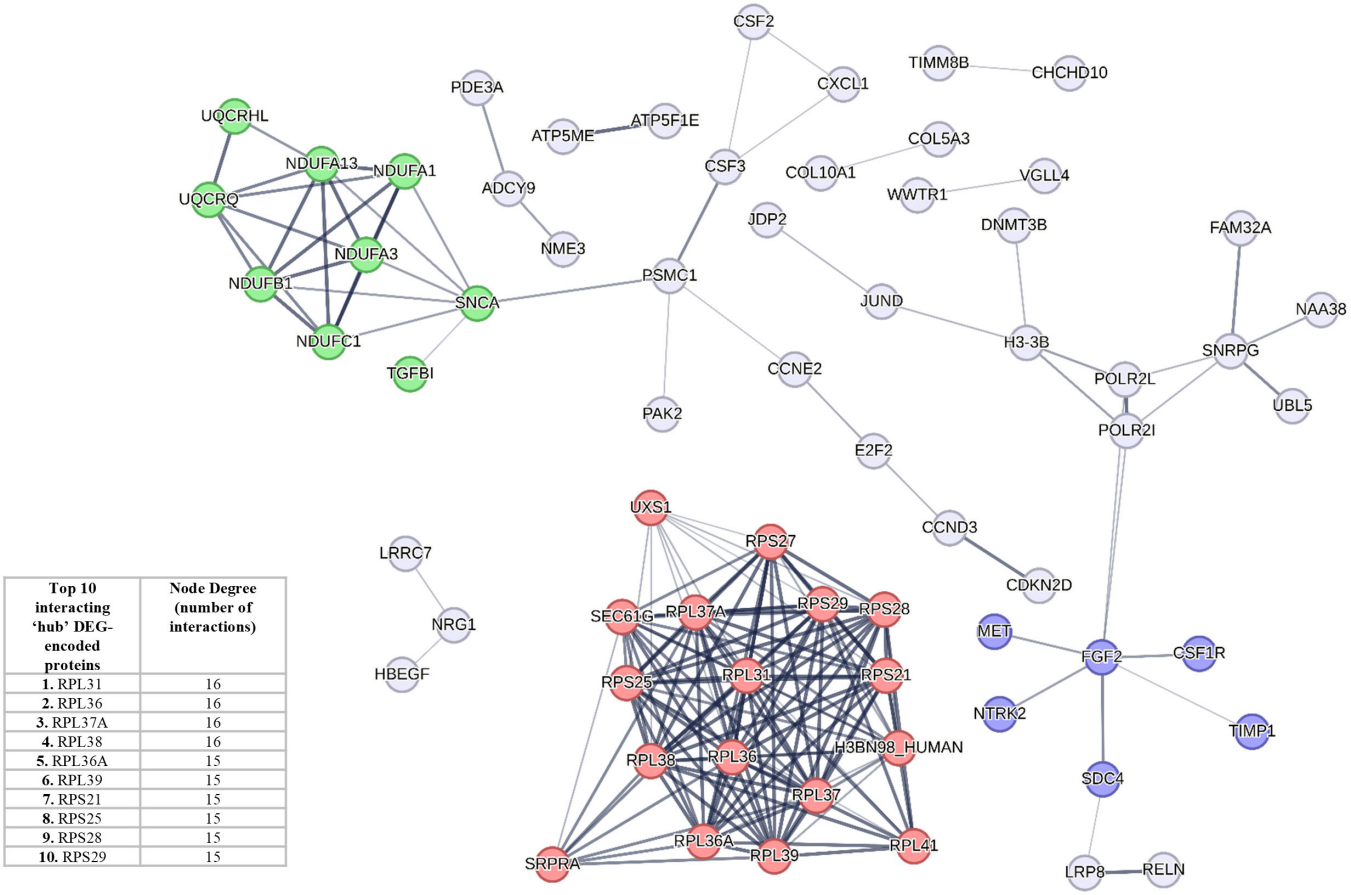
HK2KD IN2045 differentially expressed genes are associated with the ribosome. STRING network of interactions between proteins encoded by HK2KD IN2045 differentially expressed genes (DEGs). Potential protein-protein interactions (PPIs) were identified (by a medium confidence score of 0.4 or greater) upon STRING and assembled into a network. The thickness of lines indicates the strength of data support/degree of confidence supporting the predicted interaction between DEGs. Clusters of DEGs with a ‘high’ interaction score were identified upon the STRING MCL algorithm. Clusters ≥ 5 PPIs are indicated by distinct colours. The top 10 ‘hub’ DEG-encoded proteins with the highest interaction counts (node degree) are listed in the embedded figure table and represent the principal drivers of connectivity within this STRING network.

67 out of the 231 HK2KD IN2045 DEGs formed 173 total PPIs upon STRING, with 3 clusters ≥ 5 PPIs (**Figure 5**; **Supplementary Table 14**). Complementing the enrichment of ribosome-related BP and KEGG pathways, the largest cluster in the IN2045 HK2KD PPI network (shown in pink/red, n= 17 DEG-encoded proteins) was largely composed of structural components of the ribosome (**Figure 5**). These components included constituents of both the small 40s (RPS21, RPS25, RPS27, RPS28, RPS29) and large 60s (RPL31, RPL36, RPL36A, RPL37, RPL37A, RPL38, RPL39, RPL41) ribosomal subunits, which were all downregulated (**Figure 5**).

### 3.4 Identification of candidate compounds with transcriptional concordance to HK2KD

Previous efforts to translate HK2 inhibitors into the glioblastoma standard of care have been hindered by concerns about the efficacy, selectivity, and BBB permeability of the prior lead pharmacological inhibitors 3-Bromopyruvate, 2-DG, and Lonidamine (51–58).

To address this, we hypothesised that small molecule compounds capable of suppressing the HK2-associated transcriptional programme (i.e. downregulate both *HK2* and its associated transcripts) in our model glioblastoma cell cultures could phenocopy HK2KD and thus represent candidate therapeutic agents to treat *HK2* overexpressing glioblastoma patient tumours. To this end, we performed drug perturbation gene set enrichment analysis (dpGSEA) (59, 60) using the Connectivity Map (CMap) library (45, 61) upon DEGs consistently downregulated following HK2KD in both U87-MG and IN859, reasoning that these genes may reflect a core dependent transcriptional programme related to the high basal expression of *HK2* of these cell cultures rather than stress or compensatory responses activated following its knockdown. Using this approach, 24 SMCs whose downregulated gene signatures showed a negative normalised enrichment score (NES) and a GSEA standard FDR-adjusted p value ≤0.25 in both HK2KD U87-MG and IN859 were predicted as compounds whose transcriptional signatures phenocopy HK2KD for glioblastoma cells upregulating *HK2* (**Table 2**).

**Table 2:** Small−molecule compounds whose transcriptional perturbation signatures overlap with HK2KD-associated gene expression changes.

Small molecule compound	HK2KD U87-MG normalised enrichment score (padj)	HK2KD IN859 normalised enrichment score (padj)	Enriched overlapping downregulated differentially expressed genes in HK2KD U87-MG	Enriched overlapping downregulated differentially expressed genes in HK2KD IN859
0179445-0000	-1.65 (0.078)	-1.69 (0.133)	*HK2, GTPBP2, PSRC1, TCAF1*	*HK2*
Apigenin	-1.41 (0.102)	-1.42 (0.177)	*HK2, HILPDA, SERPINB2, ZBTB1, TNFAIP8, ZNF395, AMPD3, CALM1, ARNT, SLC16A1, HCAR3, SERPINB8, ANXA8L1, TLE4, HCK, SLC39A14, ANXA8, BNIP3, PTX3, ADORA2B, HEXIM1, TUBB6, TBC1D8, PLEKHO1, NGLY1, LXN, IKBKE*	*HK2, STC2, SLC16A1, ADCY9, ZNF395, INHBE, ARHGEF40, BNIP3, CALM2, SERPINB1, ARNT, ADORA2B, SLC39A14, PLIN2, IVNS1ABP, LXN, PWP2, PDCD4, LMNB1, TLE4, ADCY7, TNFAIP8, DIMT1, HILPDA, SAP30, DIXDC1, CCDC85B, GALNT11, HNRNPA0, FUCA1, OIP5, CUTC, FUT4, CALM1, RNF44, HEATR3, RUNX3, PTX3, SLC6A8, SRSF7, DLX2, NUDT4, CDCA3, BMP2K*
Betulinic acid	-1.75 (0.051)	-1.86 (0.107)	*HK2, HILPDA, ADM, ZNF395, HCAR3, NDRG1, TXNIP, ENO2, BNIP3, INSIG2, TMEM45A*	*HK2, TXNIP, P4HA1, ZNF395, BNIP3, PLIN2, ENO2, PFKFB3, PLOD2, CXCR4, HILPDA*
Dequalinium chloride	-1.79 (0.024)	-1.89 (0.107)	*HK2, HILPDA, ADM, BHLHE40, EGR1, JUN, ZNF395, DDIT4, EGR2, TPBG, HCAR3, NDRG1, TXNIP, MAFF, CCL3, ENO2, BNIP3, RGS2, TMEM45A, PTGS2*	*HK2, DDIT4, TXNIP, STC2, CDKN1A, P4HA1, ZNF395, BNIP3, TPBG, EGR1, PLIN2, TSC22D3, ENO2, PFKFB3, PLOD2*
Digoxigenin	-1.78 (0.029)	-1.82 (0.117)	*HK2, HILPDA, BHLHE40*	*HK2*
Ellipticine	-1.80 (0.011)	-1.98 (0.053)	*HK2, HILPDA, CXCL8, ADM, MTHFD2, TNFRSF1B, SLC7A5, DDIT4, F8A2, PLAUR, CCL3, SLC39A14, MTMR4, KCNN4, DPY19L4, JUND, SLC16A3, NBN*	*HK2, DDIT4, MTHFD2, SLC16A3, PCK2, EIF4A2, TRIB3, SLC1A4, SLC39A14, PLIN2, SLC7A5, DPY19L4, SLC38A2, UBE2S, JUND, DIMT1, HILPDA, RNF41, CCDC85B*
Etacrynic acid	-1.69 (0.051)	-1.83 (0.051)	*HK2, SRGN*	*HK2*
Etoposide	-1.54 (0.154)	-1.89 (0.107)	*HK2, ADM, SLC39A14*	*HK2, CENPA, CDC20, UBE2C, SLC39A14, PSRC1*
Etynodiol	-1.84 (0.010)	-1.70 (0.127)	*HK2, HILPDA, CXCL8, IL1B, ADM, BHLHE40, EGR1, ZNF395, EGR2, HCAR3, NDRG1, ICAM1, ENO2, BNIP3, RGS2, TMEM45A, PTGS2, PLEKHO1*	*HK2, P4HA1, ZNF395, BNIP3, EGR1, PLIN2, ENO2, PFKFB3, PLOD2, HILPDA, ATF3, TMEM45A*
HC Toxin	-1.34 (0.234)	-1.71 (0.127)	*HK2, F8A2, ZNF318, WNT5A, ZNF559-ZNF177, MTMR4*	*HK2, MOCS2, SRD5A1, ZNF395, GRSF1, MINPP1, EIF4A2, ARHGEF40, CALHM2, TST, TRIB3, GSAP, CHMP3, SLC25A32, PER2, PLIN2, C9orf40, IVNS1ABP, AKTIP, ENOSF1, OXLD1, SPDL1, LXN, TRAK2, CD3EAP, ADCY7, HILPDA, KLHL23, TRIOBP, EXOSC7, CCDC85B, ORAI3, TMEM45A, CPOX, OIP5, CUTC, DUSP7, PAGR1, ANXA4, TMEM243, MTMR4, UGDH, GRAMD4, ACSL3, ATP10A, CNPY2, SRSF7*
Helveticoside	-1.62 (0.087)	-1.78 (0.124)	*HK2, ZBTB1, ACKR3, ARTN, YAP1*	*HK2, STC2, SEMA4C, TRIP6, YAP1, CALHM2, TRIB3, NRP1, PER2, CTDSP1, PPRC1, ACKR3, FJX1, TMPO, LRFN4, CCND1*
Ketoconazole	-1.71 (0.070)	-1.64 (0.146)	*HK2, HILPDA, ADM, ZNF395, HCAR3, NDRG1, CCL3, ENO2, BNIP3, RGS2, TMEM45A, PTGS2*	*HK2, P4HA1, ZNF395, BNIP3, PLIN2, ENO2, PFKFB3, PLOD2, HILPDA*
Lanatoside C	-1.42 (0.076)	-1.50 (0.107)	*HK2, HILPDA, MOCS2, ADM, BHLHE40, TRAK2, SCML1, ZNF395, DDIT4, AMPD3, F8A2, TGIF1, NDRG1, MINPP1, RAC2, GRSF1, MTMR4, BNIP3, POP5, ADORA2B, SLC25A32, DPYSL2, INSIG2, TMEM45A, DICER1, PLEKHO1, PRDM4, LXN, SPDL1, PER2, DUSP6*	*HK2, DDIT4, PLAU, MOCS2, CHAC1, SRD5A1, ZNF395, GRSF1, MINPP1, TGIF1, EMP1, ARHGEF40, BNIP3, DPYSL2, SERPINB1, CALHM2, TST, TRIB3, SCML1, DICER1, KLF11, ADORA2B, SLC25A32, PER2, RAC2, PLIN2, PPIA, C9orf40, POP5, IVNS1ABP, OXLD1, SPDL1, LXN, TRAK2, CD3EAP, ADCY7, DIMT1, HILPDA, KLHL23, ELOVL6, TRIOBP, EXOSC7, TMEM45A, TMPO, OIP5, CUTC, FUT4, DUSP7, PAGR1, ANXA4, TMEM243, DUSP6, MTMR4, HEATR3, GRAMD4, ATP10A, CNPY2, SRSF7, RCL1, ERLIN1, CDKN2C, RGS10, PRDM4, HNRNPDL, HLTF*
Luteolin	-1.38 (0.150)	-1.407 (0.205)	*HK2, HILPDA, SERPINB2, USP46, TNFAIP8, AMPD3, SLC16A1, HCAR3, SERPINB8, GSAP, TLE4, HCK, TMEM45A, HEXIM1, TBC1D8, LXN*	*HK2, USP46, MARCKSL1, SLC16A1, ADCY9, SLC17A9, GSAP, PLIN2, SNTB1, ENOSF1, LXN, PWP2, CD3EAP, TLE4, ADCY7, TNFAIP8, DIMT1, HILPDA, KLHL23, DIXDC1, CCDC85B, TMEM45A, FUCA1, OIP5, CUTC, FUT4, ITPR1, SRSF7, DLX2, BMP2K*
Ly-294002	-1.60 (0.121)	-1.78 (0.117)	*HK2, CXCL8, DDIT4, SERPINE1, SPHK1*	*HK2, DDIT4, SERPINE1*
Menadione	-1.60 (0.102)	-1.99 (0.097)	*HK2, HILPDA*	*HK2, STC2, PCK2, MAK16, TMEM120B*
MG-262	-1.36 (0.140)	-1.82 (0.043)	*HK2, HILPDA, IER3, LIF, BHLHE40, CCNG2, ZNF395, MARCKS, F8A2, HIST2H2BE, SPTSSA, SLC2A1, BCL2A1, HGSNAT, C2CD2, PTX3, ADORA2B, YWHAH, SMAD3, MTSS1, ESPL1, TMEM158, SERPIND1, PSRC1, IFIT1, IER2, DUSP6, KRT15, EPB41L4B, PECAM1, FAM111A, HIVEP2, B3GNT2, CREBZF, BHLHE41*	*HK2, PLAU, SLC2A1, CARD10, FOXA2, GPRC5C, IER2, ZNF395, TSPAN15, EMP1, C2CD2, HIST1H2AC, FZD4, TMEM158, SERPINB1, PTHLH, NRP1, ADORA2B, HMGB3, PSRC1, ARL6IP5, YWHAH, STX1A, OXLD1, KIFC1, MCAM, CTDSP1, LMNB1, HGSNAT, RIN2, HILPDA, PPM1H, SPRY2, FJX1, TRIOBP, CCDC85B, GALNT11, SMAD3, AURKA, B3GNT2, AURKB, C11orf68, ZNF365, LIMA1, LRFN4, CCND1, DUSP7, MARCKS, GALNT12, PIAS3, NNMT, DUSP6, PTX3, CCNG2, GAS2L1, FBXO5, TOP2A, RNASE4, ERLIN1*
Ouabain	-1.41 (0.079)	-1.41 (0.127)	*HK2, HILPDA, MOCS2, ADM, BHLHE40, TRAK2, ZNF395, DDIT4, TPBG, SWAP70, F8A2, MINPP1, ZNF318, RAC2, GRSF1, MTMR4, POP5, ADORA2B, SLC25A32, CMAS, INSIG2, DICER1, CYTH4, PLEKHO1, PRDM4, MXI1, LXN, SPDL1, PER2, DUSP6, MAK16, PECAM1, CD9, STAMBP*	*HK2, DDIT4, MOCS2, CHAC1, SWAP70, ZNF395, GRSF1, MINPP1, MAK16, ZNF318, CYTH4, SERPINB1, CALHM2, TST, TPBG, TRIB3, DICER1, NRP1, ADORA2B, SLC25A32, PER2, RAC2, PLIN2, POP5, IVNS1ABP, ENOSF1, OXLD1, SLC1A5, SPDL1, LXN, TRAK2, CD3EAP, CD9, DIMT1, HILPDA, EXOSC7, ORAI3, TMPO, CPOX, OIP5, CUTC, FUT4, DUSP7, PAGR1, ANXA4, TMEM243, DUSP6, MTMR4, HEATR3, SLC43A3, CNPY2, SRSF7, BMP2K, MXI1, ERLIN1, CDKN2C, RGS10, PRDM4*
Piperlongumine	-1.61 (0.024)	-1.50 (0.131)	*HK2, HILPDA, CXCL8, TNFRSF1B, IER3, SERPINB2, NLRP3, ZBTB1, BHLHE40, TNFAIP8, RRAGA, TREM1, ZNF395, CCL20, ALCAM, F8A2, PPCS, GSAP, TGIF1, WIPF1, ZNF318, PLAUR, BCL2A1, CCL3, RCAN1, RGS2, POP5, KCNN4, TBC1D8, TMEM158, CLDND1, PSRC1, SLC16A3, NBN, P2RX7, POGZ, IMPA1, PECAM1, FAM111A, CREBZF, PAPOLA, TMPO, OSTM1, CHEK1*	*HK2, PPCS, SLC16A3, ADCY9, ZNF395, WIPF1, TGIF1, EMP1, ZNF318, PTPN22, TMEM158, SERPINB1, GSAP, NRP1, SMAD2, P2RX7, HMGB3, PSRC1, PLIN2, RCAN1, POP5, IVNS1ABP, ECT2, C3AR1, ADAM10, CTDSP1, DCK, CXCR4, ADCY7, TNFAIP8, DIMT1, HILPDA, CKLF, RPL37A, TMPO, ALCAM, CPOX, FUT4, COMMD8, OSTM1, SLC43A3, EFR3A, TRIM13, HNRNPDL, TBC1D8, NUDT15, BTG2, RGS19, ADSS, FAM216A, PIK3CB, HMGXB4, MAD2L1, PBK, OGT, P2RX4, RRAGA, GCOM1, TNFRSF1B, RYK*
Puromycin	-1.39 (0.162)	-1.72 (0.107)	*HK2, HILPDA, ADM, SLCO4A1, SLC25A17, SLC16A1, RPL17-C18orf32*	*HK2, RAB40B, SLC16A3, SLC25A17, SLC16A1, PRDX4, KLF11, PLIN2, FSCN1, CISH, CD3EAP, PLOD2, PPRC1, POLR2H, HILPDA, PDIA5, ELOVL6, CCDC85B, GALNT11, DCTPP1, RPL37A, VCAN, RUSC1, FUT4, INSIG1, RNF44, DUSP6, SLC6A8, GRAMD4, SRSF7, RCL1, DKC1, POLG2, FASN*
Pyrvinium	-1.74 (0.050)	-1.71 (0.128)	*HK2, HILPDA, ADM, ZNF395, AMPD3, HCAR3, NDRG1, TXNIP, CCL3, ENO2, BNIP3, TMEM45A, DHRS3, TUBB6*	*HK2, TXNIP, P4HA1, ZNF395, BNIP3, DHRS3, PDK1, PLIN2, ENO2, PFKFB3, PLOD2, HILPDA, KLHL23, ELOVL6*
Strophanthidin	-1.55 (0.093)	-1.43 (0.206)	*HK2, HILPDA, BHLHE40, F8A2, ZNF318, WNT5A, DICER1, IFIT1, POLQ*	*HK2, ZNF318, DICER1*
Trichostatin A	-1.42 (0.080)	-1.69 (0.059)	*HK2, FAM136A, CXCL8, SERPINB2, ZBTB1, BHLHE40, OTUD4, OGFRL1, DDIT4, AMPD3, ZNF24, SERPINB8, ANXA8L1, GSAP, TGIF1, SIRPA, EVI2B, VEGFA, PIK3CD, WIPF1, HCK, PLAUR, TRPM2, NFKBIA, TOR1AIP1, CCL3, ANXA8, ADORA2B, YWHAH, CARD8, KCNN4, PLEKHO1, GNA15, BID, NBN, KBTBD2, ETV6, PECAM1, NCOA6, TRIM21, FMNL1, GBE1, TMPO, TOR1A, ATF1, CD58, STK10, ARHGEF40, SRSF4, PMF1, RCBTB2, ZCCHC8, FUT4, METTL4, TNF, PSMB10, CLEC11A, TMF1, ASXL1, WEE1, CCL3L1, HNRNPL, NUDT15*	*HK2, DDIT4, PLAU, FAM136A, STC2, OGFRL1, VEGFA, TRIP6, RPL10, PCK2, MID1IP1, OTUD4, WIPF1, TGIF1, ARHGEF40, TOR1AIP1, CALHM2, ADGRE5, TRIB3, GSAP, NRP1, ATF1, SLC1A4, ADORA2B, ZNF24, TRPM2, HMGB3, PLIN2, YWHAH, GYPC, WEE1, ENOSF1, VLDLR, C3AR1, BCOR, JADE1, PSMB10, NUDT3, CTDSP1, YARS, LMNB1, CXCR4, ADCY7, PPRC1, SPRY2, HOMER3, RASSF1, CEBPG, SNX5, VCAN, TMPO, FUT4, CCNA2, MEF2D, RNF44, GRAMD4, SLC43A3, ATP10A, SLC7A11, BAZ1A, ADAP1*
Vorinostat	-1.41 (0.083)	-1.70 (0.051)	*HK2, FAM136A, CXCL8, ADM, SERPINB2, ZBTB1, BHLHE40, OTUD4, OGFRL1, DDIT4, AMPD3, SERPINB8, E2F3, ANXA8L1, GSAP, TGIF1, SIRPA, EVI2B, VEGFA, PIK3CD, WIPF1, HCK, PLAUR, TRPM2, NFKBIA, NUCKS1, TOR1AIP1, CCL3, ANXA8, TYMS, ADORA2B, YWHAH, CARD8, KCNN4, PLEKHO1, AK4, GNA15, BID, NBN, BTG3, KBTBD2, PECAM1, NCOA6, FMNL1, GBE1, TMPO, TOR1A, ATF1, CLK1, CD58, STK10, ARHGEF40, SRSF4, PMF1, RCBTB2, ZCCHC8*	*HK2, DDIT4, PLAU, FAM136A, STC2, OGFRL1, VEGFA, TRIP6, RPL10, PCK2, MID1IP1, OTUD4, WIPF1, TGIF1, AK4, ARHGEF40, E2F3, TOR1AIP1, CALHM2, ADGRE5, TRIB3, GSAP, NRP1, ATF1, SLC1A4, ADORA2B, TRPM2, HMGB3, ANPEP, PLIN2, YWHAH, TYMS, GYPC, WEE1, ENOSF1, ERF, VLDLR, C3AR1, BCOR, JADE1, PSMB10, RCC1, NUDT3, CTDSP1, YARS, LMNB1, CXCR4, ADCY7, PPRC1, FOSL2, SPRY2, HOMER3, RASSF1, CEBPG, SNX5, ORAI3, BTG3, VCAN, TMPO, FUT4, CCNA2, RNF44, GRAMD4, SLC43A3, ATP10A, SLC7A11, BAZ1A, ADAP1, ERLIN1, DEAF1, TRIM13, SLC35F2, PIK3CD, MFNG, DKC1, NUDT15, SLC43A1, XAF1, NUCKS1, EIF2S2, RGS19*

## 4 Discussion

Current standard of care treatment for glioblastoma is largely ineffective, associated with numerous side effects, and has remained largely unchanged for the past 20 years. As a result, there is a desperate need for effective new therapies that can selectively target this tumour. With *HK2* established to be overexpressed in glioblastoma and possessing limited expression in normal adult brain (19–22, 26, 27), targeting HK2 and/or its activity has been proposed as a means of selectively eliminating Warburg metabolising glioblastoma cells whilst limiting toxicity to surrounding healthy tissue. Despite this, there has been limited research to date exploring the wider transcriptional changes that may occur following therapeutic inhibition of HK2 in glioblastoma. In this study, we sought to address this gap by characterising the gene expression changes that occur following inhibition of *HK2* expression in glioblastoma cell cultures, with the aim of identifying potential co-targets for future metabolic therapeutic strategies to treat these tumours.

To begin, we first verified that our model glioblastoma cell cultures maintained the characteristic upregulation of *HK2* expression seen in patient tumours. We found that the majority of our cell cultures exhibited upregulated *HK2* expression relative to control human adult normal brain tissue, recapitulating its overexpression previously reported in patient tumours (19–22, 26, 27). The established cell line U87-MG and the patient-derived short-term cell culture IN859 were found to display the highest upregulation of *HK2*. A small number of other patient-derived short-term cell cultures, such as IN2045, contrastingly downregulated *HK2* or completely lacked its expression, suggesting they may rely upon alternative metabolic pathways to produce energy rather than HK2-driven glycolysis. This metabolic diversity, consistent with other studies (62–65), reflects the characteristic heterogeneity of glioblastoma while also signalling a need for future therapeutic strategies to consider simultaneously targeting additional metabolic pathways alongside Warburg glycolysis.

Afterwards, we sought to assess whether upregulation of *HK2* was associated with glycolytic activity in these cell cultures. To investigate this, we inhibited *HK2* expression by siRNA-mediated HK2KD and evaluated its effect on glycolytic activity using the Seahorse XFp glycolytic stress test. HK2KD was subsequently found to significantly inhibit the basal glycolytic activity of high *HK2*-expressing U87-MG and IN859. U87-MG’s decline in glycolytic activity after HK2KD was consistent with that observed in other studies (20, 26). On the other hand, HK2KD had no significant impact upon the glycolytic activity displayed by low *HK2*-expressing IN2045, validating HK2’s importance in driving elevated glycolytic activity in glioblastoma cell cultures that specifically upregulate its expression. Nevertheless, basal glycolytic activity was still observable following HK2KD in U87-MG and IN859. While *HK1* has previously been reported to be downregulated in glioblastoma by our group and others (21, 26), it may be beneficial to investigate the impact of co-HKKD (i.e., *HK1*/*3*/*4* + *HK2*) on ECAR in our cell cultures to identify the potential driver of this residual glycolytic activity.

RNAseq was subsequently performed on HK2KD cell cultures to investigate how inhibition of *HK2* expression is associated with changes in the glioblastoma transcriptome. U87-MG and IN859, which exhibited the highest *HK2* overexpression among the model cell cultures, were selected for analysis alongside low *HK2*-expressing IN2045 which was included as a comparative control. This RNA-seq analysis identified a rich array of genes differentially expressed in each cell culture after HK2KD, which were then examined using STRING PPI networks and GO/KEGG functional enrichment analyses. While selected shared DEGs (*FGF2*, *MET*, *PDHX*, *SDC4*, and *TGFBI*) common to all the HK2KD cell cultures were validated by RT–qPCR, the majority of DEGs discussed herein were solely inferred from RNA-seq analysis, with associated pathway-level interpretations based on enrichment and network analyses. Functional consequences of these transcriptional changes were not directly assessed and therefore remain to be experimentally validated. Notably, no BPs or KEGG pathways related to glycolysis or glucose metabolism were enriched in HK2KD U87-MG or IN859. With HK2KD indicated to significantly reduce glycolytic activity, it is plausible that proteomic changes in glycolytic and other metabolic pathways (62–65) may have occurred in these previously *HK2*-overexpressing cell cultures post-HK2KD.

### 4.1 HK2KD is associated with immuno-inflammatory transcriptional changes in glioblastoma

The most striking and conceptually important finding of this study was the divergent inflammatory and immune transcriptional responses observed following HK2KD in U87-MG and IN859. In HK2KD U87-MG, immune- and inflammation-related GO BPs were predominantly associated with negative z-scores, indicating broad downregulation of gene expression programmes associated with inflammatory signalling. In contrast, HK2KD IN859 displayed a more mixed response, with a substantial subset of immune-related processes showing positive z-scores, suggesting activation of specific inflammatory and immune expression programmes.

The enrichment for immune- and inflammation-related GO BPs and KEGG pathways in HK2KD U87-MG was driven in part by the downregulated expression of several cytokines known to be integral to the development of chronic neuroinflammation in glioblastoma. These included the interleukins *IL-1α*, *IL-1β* and *IL-6*, the Tumour Necrosis Factor (TNF) family members *TNFα* and *TNFSF14* (also known as LIGHT), and Colony Stimulatory Factor 2 (*CSF2*, also known as GM-CSF) (66–72), that can drive both autocrine tumour growth and paracrine activation/recruitment of immune cells to create a self-perpetuating inflammatory tumour microenvironment (TME) (73). Downregulation of the receptor *TNFRSF1B* (also known as TNFR2), which is selectively activated by the membrane-bound form of TNF-α (74), also suggested that HK2 may be associated with transcriptional modulation of TNF juxtacrine signalling in glioblastoma.

The expression of several inflammatory chemokines were also found to be downregulated in HK2KD U87-MG. Inflammatory chemokines are recognised for their ability to chemoattract leukocytes toward sites of inflammation and activate inflammatory signalling pathways that may directly promote tumour cell growth (75–77). Expression of the CXCL subfamily of chemokines was particularly disrupted in HK2KD U87-MG, with *CXCL1*, *CXCL2*, *CXCL3*, *CXCL5*, *CXCL6* and *CXCL8* all downregulated. Similarly, the CCL subfamily chemokines *CCL3*, *CCL3L1* and *CCL20*, were also downregulated. CXCLs can recruit CXCR 1 and 2 expressing neutrophils to the tumour, while CCL3 and CCL3L1 promote CCR5-mediated monocyte infiltration. Within the glioblastoma TME, these recruited myeloid populations are frequently driven toward a pathologically activated, immunosuppressive state through sustained exposure to cytokines such as IL-1β, IL-6, and CSF2, acquiring Myeloid-Derived Suppressor Cell (MDSC)-like phenotypes (76, 78–82). In this TME, monocytic MDSCs can further differentiate into ‘tumour-associated’ macrophages (TAMs), which reinforce immunosuppression and paracrinally promote glioblastoma inflammation and progression (81, 83). Given that myeloid cells can constitute up to 30-50% of the glioblastoma tumour mass and that MDSCs suppress cytotoxic T lymphocyte (CTL) activity while promoting regulatory T cell (Treg) expansion, the observed chemokine downregulation following HK2KD in U87-MG suggests reduced transcriptional support for immunosuppressive myeloid recruitment within the glioblastoma tumour microenvironment (69, 84, 85). CCL3/CCL3L1 and CCL20 are additionally recognised to promote Treg recruitment via CCR5 and CCR6, respectively (76). This control of Treg levels is of particular clinical relevance given that Tregs inhibit CTL activity via immune checkpoint pathways, including CTLA-4 and PD-L1, which represent key immunotherapeutic targets in glioblastoma (86–88). Collectively, these expression changes in HK2KD U87-MG suggested that HK2 is associated with transcriptional programmes linked to chronic inflammation and immunosuppression within the glioblastoma TME. However, this effect was not uniform, with HK2KD in IN859 eliciting a distinct chemokine response.

In IN859, HK2KD was associated with enrichment of positive z-score immune- and inflammation-related GO BPs and the cytokine-cytokine receptor interaction KEGG pathway, which was driven in part by upregulation of various CXCL chemokines (*CXCL1*, *CXCL2*, *CXCL3*, and *CXCL6*) alongside increased expression of chemokine receptors *CCR3* and *CXCR4*. In contrast to U87-MG, this upregulation suggests that HK2KD in IN859 may be associated with increased expression of programmes related to chemokine-mediated immune signalling pathways. Given the established roles of CXCL chemokines in recruiting neutrophils and other myeloid populations, their upregulated expression in response to HK2KD may reflect transcriptional programmes associated with immune cell trafficking to the glioblastoma TME; however, the functional consequence of this remains context-dependent. Under tumour-permissive cytokine conditions, this may be associated with gene expression patterns linked to the accumulation of immunosuppressive MDSC and TAM phenotypes (76, 78–83). Alternatively, in a less suppressive baseline context, these transcriptional changes could be associated with gene expression patterns linked to more acute, immunostimulatory responses (89–91).

One potential explanation for the divergence between HK2KD U87-MG and IN859 is that they may reflect distinct transcriptional glioblastoma states. In line with the well-characterised heterogeneity of glioblastoma, large-scale transcriptomic and single-cell analyses have demonstrated that these tumours exist along a spectrum ranging from proneural-like, proliferative states through to classical-like and more mesenchymal-like states characterised by distinct transcriptional programmes as well as different immune- and inflammation-related profiles (92–94). Within this framework, the divergent response to HK2KD observed here may indicate that U87-MG and IN859 occupy distinct positions along this transcriptional-immune continuum. Mesenchymal-like glioblastoma, which is strongly associated with tumour recurrence and poorer prognosis, is characterised by a highly inflammatory immunosuppressive phenotype, including extensive immune cell infiltration, elevated expression of inflammatory mediators, and activation of NF-κB and STAT3 signalling pathways. Proneural-like glioblastoma, on the other hand, generally exhibits a lower inflammatory tone and reduced immune infiltration (92, 93, 95–97). While formal subtype classification was not performed in this study, the coordinated downregulation of inflammatory and chemokine-related DEG networks following HK2KD in U87-MG is suggestive of disruption to a mesenchymal-like, inflammation-driven programme, although definitive subtype assignment would require global transcriptomic profiling. In contrast, the induction of chemokine expression in HK2KD IN859 may reflect a comparatively lower baseline inflammatory state, potentially consistent with a proneural-like transcriptional programme, in which metabolic perturbation elicits compensatory or stress-associated immune responses.

HK2 has previously been implicated in the regulation of inflammatory and immune processes in glioblastoma, with its expression reported to correlate with tumour infiltration by various immune cells (including neutrophils, monocytes and Tregs) and the release of inflammatory mediators such as IL-1 and CXCL8 (19). *HK2* levels have likewise also been positively associated with the expression of various inflammatory cytokines in several inflammatory-related diseases such as rheumatoid arthritis (98), whose KEGG pathway was found to be enriched in HK2KD U87-MG, supporting a broader role for HK2 in regulating inflammatory gene programs.

At a signalling level, HK2 has been shown to non-canonically phosphorylate Inhibitor of nuclear factor kappa B (IκBα) in both glioblastoma and breast cancer cells, leading to the activation and nuclear accumulation of the key inflammatory transcription factor NFκB (99, 100). NFκB is acknowledged to promote the expression of several cytokines and chemokines (i.e. CXCL1–2, CXCL8, IL1β, IL6, TNFα, CSF2) that were downregulated in HK2KD U87MG (101, 102), thereby suggesting that diminished NF-κB signalling may contribute to the inflammatory transcriptional alterations observed in this model following HK2KD.

In addition to NF-κB signalling, HK2 may be associated with transcriptional changes related to inflammasome, which assembles to initiate caspase 1-dependent secretion of IL-1β and IL-18 (103). NLR Family Pyrin Domain Containing 3 (*NLRP3*) and Mediterranean Fever (*MEFV*, also known as pyrin), which act as sensor components to form functional inflammasomes, were downregulated in HK2KD U87-MG (104). Several NLRP3 inflammasome activators (*HCK*, *NEK7*, *TXNIP*) were additionally downregulated across the HK2KD cell cultures. In support of this, HK2’s dissociation from mitochondria has previously been associated with enhanced NLRP3 inflammasome activity (105, 106), suggesting that HK2KD may alter glioblastoma inflammasome-dependent cytokine production.

Importantly, these putative signalling relationships provide a framework for understanding the opposing inflammatory-related gene expression profiles in HK2KD U87-MG and IN859. While the expression changes observed in HK2KD U87-MG appear to indicate suppression of NF-κB- and inflammasome-associated cytokine networks, the upregulation of CXCL chemokines in IN859 suggests that alternative signalling pathways may compensate for or override this effect.

Together, these observations suggest that HK2 is associated with immunometabolic transcriptional programmes in glioblastoma, linking glycolytic activity and potential non−canonical roles of HK2 to inflammatory and immune−related gene expression patterns. With the heterogeneity observed between HK2KD U87-MG and IN859, HK2 inhibition is unlikely to elicit uniform immunomodulatory transcriptional responses across all glioblastomas. Instead, HK2 targeting may be associated with transcriptional changes consistent with reduced chronic immunosuppressive inflammation or stress-associated inflammatory signalling, depending on tumour-specific immune and transcriptional contexts. Importantly, these proposed immunomodulatory effects remain inferred solely from transcriptional profiling and therefore require functional validation (e.g., cytokine secretion assays and conditioned-media chemotaxis assays) in more physiologically relevant co-culture, organotypic, and *in vivo* models of the TME. Such studies will be necessary to better understand these inferred context-dependent responses, which may prove critical for the clinical translation of HK2-directed therapies and their potential combination with immunotherapy.

### 4.2 HK2KD disrupts angiogenesis-associated transcriptional programmes in glioblastoma

Several BPs associated with angiogenesis, which is key to glioblastoma progression, were notably also found to be enriched in HK2KD U87-MG (107, 108). HK2KD has previously been shown to reduce glioblastoma tumour angiogenesis *in vivo* (20, 26); however, the specific underlying mechanisms involved have yet to be elucidated. One possible explanation may lie within the numerous inflammatory cytokines and chemokines downregulated in HK2KD U87-MG that are also acknowledged to play a key role in facilitating tumour blood vessel formation, and, in turn, promote the greater infiltration of inflammatory and immune cells into the tumour (109–111). These angiogenic-linked cytokines included *IL-1α*, *IL-1β*, *IL-6*, *TNF-α*, and *CSF2*, which are acknowledged to promote endothelial cell inflammatory activation and proliferation, as well as induce the release of pro-angiogenic factors such as VEGF (110–114). Downregulation of the chemokine *CXCL8* (also known as IL-8) was also particularly significant given its established potent proangiogenic activity (110, 115–117). Other prominent angiogenic factors downregulated in HK2KD U87-MG included the growth factor Angiopoietin 1 (*ANGPT1*), which promotes vessel maturation and stability in later vascular development (108), and the pro-angiogenic peptide Adrenomedullin (*ADM*) (118).

HK2KD IN859 most notably downregulated the primary initiator of tumour angiogenesis, *VEGFA* (108, 119). Previous research has demonstrated that HK2KD similarly induces downregulation of this VEGF isoform in other glioblastoma cell cultures (20, 26, 120). VEGFA has been extensively investigated as a therapeutic target in glioblastoma, with treatment using the neutralising humanised monoclonal antibody bevacizumab achieving limited clinical success (121). A further key pro-angiogenic growth factor, *FGF2*, was found to be downregulated across all the HK2KD cell cultures (108). FGF2 signalling has been associated with promoting glioblastoma proliferation, invasion, radioresistance, and stemness (122–126). Expression of the transmembrane heparan sulphate proteoglycan Syndecan-4 (*SDC4*), which binds and stabilises FGF2 to promote prolonged high-affinity interaction with its receptors, was interestingly also downregulated in each HK2KD cell culture (127). Several matrix metalloproteinases (MMPs) acknowledged to play a key role in facilitating angiogenic matrix breakdown and glioma invasion were additionally downregulated in HK2KD U87-MG (*MMP-9*) and IN859 (*MMP-2, 7*) (108, 128–133). One possible explanation for the observed angiogenesis-related transcriptional changes is the downregulation of the HIF2α encoding gene, *EPAS1*, in both HK2KD U87MG and IN859. HIF2-α is well established to promote angiogenesis and induce the expression of targets such as *VEGF-A*, *ANGPT1*, *MMP-2* and *MMP-9* (134–136). Lactate production linked to *HK2* expression has also been proposed to be associated with the expression of angiogenic factors such as *VEGF-A*, potentially representing an additional mechanism for these transcriptional changes (136, 137).

Consistent with previous studies linking glioblastoma *HK2* levels to their degree of angiogenesis *in vivo* (20, 26), the transcriptional alterations observed here suggest that angiogenesis-associated gene expression programmes may be disrupted following HK2KD. Therapeutic targeting of HK2 may therefore dampen the hallmark chronic angiogenesis that drives glioblastoma progression within the TME; however, functional vascular effects were not assessed in this study, and whether these transcriptional changes translate into altered tumour vascularisation remains to be experimentally validated.

### 4.3 Identification of compounds whose transcriptional signatures recapitulate HK2KD-associated gene expression patterns

As the potential clinical translation of HK2-directed therapies is currently limited by the lack of a safe and effective HK2-targeting agent (51–58), we next applied a dpGSEA-CMap transcriptome-based perturbation matching approach to our HK2KD glioblastoma datasets to identify compounds whose transcriptional signatures phenocopy HK2KD for HK2-overexpressing tumours.

This dpGSEA-CMap analysis was performed on DEGs that were consistently downregulated following HK2KD in U87-MG and IN859 cells, as these are likely to represent a shared, core transcriptional programme associated with their basal *HK2* overexpression. While HK2KD induced the upregulation of various genes, these DEGs were not included in the dpGSEA-CMap analysis, as they were considered more likely to reflect heterogeneous, context-dependent stress responses (such as ER stress) provoked by metabolic perturbation that could confound identification of SMCs capable of targeting the core HK2-linked transcriptional programme. Nevertheless, these secondary stress-associated programmes remained biologically informative and provided important transcriptomic context for interpreting identified compounds.

Using this strategy, we identified 24 SMCs that induce a downregulated transcriptional signature that overlaps with DEGs downregulated in both HK2KD U87-MG and IN859. This builds upon previous work by Agnihotri et al., who similarly applied dpGSEA to identify compounds that recapitulate HK2KD-associated transcriptional downregulation in glioblastoma (120) while extending such an approach to an additional independent cell culture (IN859) to refine the pool of transcriptomic concordance hits across distinct models.

The transcriptional profile induced by the antifungal agent Ketoconazole was enriched for downregulated DEGs identified in both our HK2KD cell cultures and those of Agnihotri et al. (120). Two natural flavonoids, luteolin and apigenin, were also among the enriched SMCs. Luteolin has previously been demonstrated to dock with HK2 and inhibit its activity (137), while apigenin has been linked to enhancing the cytotoxic effects of doxorubicin in hepatocellular carcinomas by inducing inhibition of their *HK2* expression (138). Piperlongumine, which has been shown to disrupt HK2’s interaction with its mitochondrial anchor VDAC1 to inhibit non-small cell lung cancer growth (139), was similarly also enriched.

The established proteasome inhibitor MG-262 was additionally enriched in both our and the Agnihotri et al. HK2KD cell culture datasets (120), however this compound has also been reported to exhibit toxicity toward normal human astrocyte cells (140). This enrichment for a proteasome inhibitor was particularly intriguing given the rich array of proteasome subunit components found to be upregulated in HK2KD IN859, which, while initially appearing paradoxical, can be explained by considering the downstream consequences of glycolytic disruption. Suppression of glycolysis has been established to promote ER stress and the accumulation of misfolded proteins necessitating increased engagement of protein quality control pathways involving the proteasome (141, 142). Consistent with this, several upregulated HK2KD IN859 DEGs were also associated with ERAD, which operates the transport of unfolded/misfolded proteins from the ER to the proteasome for degradation (143). These included the ATPase Valosin-Containing Protein (*VCP*), which functions to mobilise ubiquitinated unfolded/misfolded proteins from the ER membrane, and its various adaptors *NPLOC4*, *UFD1*, *UBQLN1* and *UBXN4* (144–146). The E4 ubiquitin ligase *UBE4B*, which multiubiquitylates proteins collected by the VCP/UFD1/NPLOC4 complex in ERAD, was also upregulated (147). Collectively, these changes are suggestive of an adaptive proteostatic response to metabolic stress following disruption of HK2 activity. Therefore, rather than recapitulating the core HK2-associated transcriptional programme, proteasome inhibition with MG262 may likely exploit a secondary, context-dependent reliability on proteostasis pathways induced by metabolic stress following HK2KD.

The identification of these various compounds whose transcriptional signatures phenocopy HK2KD across independent transcriptomic and functional screens highlight the pleiotropic mechanisms by which HK2-associated tumour programmes may be perturbed. Nevertheless, the dpGSEA-CMap analysis performed in this study was designed only as an exploratory hypothesis-generating drug-repurposing screen, rather than a definitive prioritisation pipeline. Accordingly, the 24 identified compounds are not presented as a ranked list of candidates. The prioritisation of individual compounds will require additional filtering, including validation of target specificity across glioblastoma models stratified by *HK2* expression, assessment of compensatory responses (e.g. metabolomic profiling), and the evaluation of toxicity in normal healthy cells (e.g. normal human astrocytes); to evaluate whether they induce functional effects consistent with HK2 inhibition as well as their clinical feasibility, which were beyond the scope of the present study.

## 5 Conclusions

In summary, our RNAseq profiling of HK2KD glioblastoma cell cultures identified extensive transcriptional remodelling extending beyond canonical glycolytic pathways, predicting that HK2 may be associated with inflammatory, immune and angiogenesis-related regulatory networks within this brain tumour. The divergent inflammatory-related expression responses observed between HK2KD U87−MG and IN859 suggest that HK2 could be associated with modulation of context−dependent immunometabolic transcriptional programmes that are potentially influenced by tumour−intrinsic transcriptional and inflammatory states. Together, these transcriptional data generate hypotheses that expand the current understanding of HK2 biology in glioblastoma beyond metabolism alone and suggest that therapeutic inhibition of HK2 may differentially affect immune-related gene expression patterns across distinct glioblastoma subtypes or cellular states. Collectively, this work supports further investigation of HK2-associated transcriptional programmes and their potential relevance to immunotherapeutic and anti-angiogenic approaches in molecularly stratified glioblastoma models.

### 5.1 Study limitations

This study has several important limitations that should be considered when interpreting the findings. First, all analyses were performed in glioblastoma cell monocultures, and therefore the transcriptional changes observed following HK2KD reflect tumour cell-intrinsic programmes rather than direct functional remodelling of the tumour microenvironment. While many of the DEGs identified encode cytokines, chemokines, and angiogenesis-related factors with established roles in immune cell recruitment and endothelial activation, the present study did not include functional validation experiments. Future studies would therefore need to incorporate approaches such as protein-level measurements, cytokine secretion assays, conditioned-media chemotaxis and endothelial tube-formation/activation assays, and co-culture or organotypic/*in vivo* models to mechanistically validate these findings. Consequently, the immunological and angiogenic implications discussed herein remain inferential and hypothesis-generating rather than experimentally demonstrated.

Second, RNAseq was performed with limited biological replication (n = 2 per condition), and the resulting differential expression analyses should therefore be interpreted as exploratory. To mitigate this limitation, we focused on concordant transcriptional patterns observed across independent glioblastoma models with high *HK2* expression, used conservative statistical thresholds, and validated selected shared differentially expressed genes by RT qPCR.

Finally, residual glycolytic activity following HK2KD suggests potential metabolic compensation by other hexokinase isoforms (i.e. HK1, 3, 4) or auxiliary metabolic pathways, which were not directly interrogated in this study and warrant further investigation.

Taken together, these limitations highlight that the present work is best viewed as a transcriptomic framework defining HK2-associated inflammatory and angiogenesis-linked programmes in glioblastoma cells, intended to inform and prioritise future functional and mechanistic studies in immune competent model systems.

## Data Availability

The datasets presented in this study can be found in online repositories. The names of the repository/repositories and accession number(s) can be found below: https://www.ncbi.nlm.nih.gov/geo/, GSE325873.

## References

[1] Stupp R Hegi ME Mason WP van den Bent MJ Taphoorn MJ Janzer RC . Effects of radiotherapy with concomitant and adjuvant temozolomide versus radiotherapy alone on survival in glioblastoma in a randomised phase III study: 5-year analysis of the EORTC-NCIC trial. Lancet Oncol. (2009) 10:459–66. doi: 10.1016/s1470-2045(09)70025-7 19269895

[2] Weller M Le Rhun E Van den Bent M Chang SM Cloughesy TF Goldbrunner R . Diagnosis and management of complications from the treatment of primary central nervous system tumors in adults. Neuro Oncol. (2023) 25:1200–24. doi: 10.1093/neuonc/noad038 36843451 PMC10326495

[3] Zhou W Wahl DR . Metabolic abnormalities in glioblastoma and metabolic strategies to overcome treatment resistance. Cancers (Basel). (2019) 11:1231. doi: 10.3390/cancers11091231 31450721 PMC6770393

[4] Bernhard C Reita D Martin S Entz-Werle N Dontenwill M . Glioblastoma metabolism: insights and therapeutic strategies. Int J Mol Sci. (2023) 24:9137. doi: 10.3390/ijms24119137 37298093 PMC10252397

[5] Maroon JC Seyfried TN Donohue JP Bost J . The role of metabolic therapy in treating glioblastoma multiforme. Surg Neurol Int. (2015) 6:61. doi: 10.4103/2152-7806.155259 25949849 PMC4405891

[6] Vaupel P Multhoff G . Revisiting the Warburg effect: historical dogma versus current understanding. J Physiol. (2021) 599:1745–57. doi: 10.1113/jp278810 33347611

[7] Shestov AA Liu X Ser Z Cluntun AA Hung YP Huang L . Quantitative determinants of aerobic glycolysis identify flux through the enzyme GAPDH as a limiting step. Elife. (2014) 3. doi: 10.7554/elife.03342 25009227 PMC4118620

[8] Epstein T Gatenby RA Brown JS . The Warburg effect as an adaptation of cancer cells to rapid fluctuations in energy demand. PloS One. (2017) 12:e0185085. doi: 10.1371/journal.pone.0185085 28922380 PMC5602667

[9] Heuser C Renner K Kreutz M Gattinoni L . Targeting lactate metabolism for cancer immunotherapy - a matter of precision. Semin Cancer Biol. (2023) 88:32–45. doi: 10.1016/j.semcancer.2022.12.001 36496155

[10] Wang Z Dai Z Zhang H Liang X Zhang X Wen Z . Tumor-secreted lactate contributes to an immunosuppressive microenvironment and affects CD8 T-cell infiltration in glioblastoma. Front Immunol. (2023) 14:894853. doi: 10.3389/fimmu.2023.894853 37122693 PMC10130393

[11] Wang S Huang T Wu Q Yuan H Wu X Yuan F . Lactate reprograms glioblastoma immunity through CBX3-regulated histone lactylation. J Clin Invest. (2024) 134. doi: 10.1172/jci176851 39545414 PMC11563687

[12] D'Aprile S Denaro S Gervasi A Vicario N Parenti R . Targeting metabolic reprogramming in glioblastoma as a new strategy to overcome therapy resistance. Front Cell Dev Biol. (2025) 13:1535073. doi: 10.3389/fcell.2025.1535073 40078366 PMC11897528

[13] Robey RB Hay N . Mitochondrial hexokinases, novel mediators of the antiapoptotic effects of growth factors and Akt. Oncogene. (2006) 25:4683–96. doi: 10.1038/sj.onc.1209595 16892082

[14] DeWaal D Nogueira V Terry AR Patra KC Jeon SM Guzman G . Hexokinase-2 depletion inhibits glycolysis and induces oxidative phosphorylation in hepatocellular carcinoma and sensitizes to metformin. Nat Commun. (2018) 9:446. doi: 10.1038/s41467-017-02733-4 29386513 PMC5792493

[15] Nawaz MH Ferreira JC Nedyalkova L Zhu H Carrasco-López C Kirmizialtin S . The catalytic inactivation of the N-half of human hexokinase 2 and structural and biochemical characterization of its mitochondrial conformation. Biosci Rep. (2018) 38. doi: 10.1042/bsr20171666 29298880 PMC5803496

[16] Ferreira JC Khrbtli AR Shetler CL Mansoor S Ali L Sensoy O . Linker residues regulate the activity and stability of hexokinase 2, a promising anticancer target. J Biol Chem. (2021) 296:100071. doi: 10.1074/jbc.ra120.015293 33187984 PMC7949118

[17] Patra KC Wang Q Bhaskar PT Miller L Wang Z Wheaton W . Hexokinase 2 is required for tumor initiation and maintenance and its systemic deletion is therapeutic in mouse models of cancer. Cancer Cell. (2013) 24:213–28. doi: 10.1016/j.ccr.2013.06.014 23911236 PMC3753022

[18] Li R Mei S Ding Q Wang Q Yu L Zi F . A pan-cancer analysis of the role of hexokinase II (HK2) in human tumors. Sci Rep. (2022) 12:18807. doi: 10.21203/rs.3.rs-1379746/v1 36335239 PMC9637150

[19] Huang Y Ouyang F Yang F Zhang N Zhao W Xu H . The expression of Hexokinase 2 and its hub genes are correlated with the prognosis in glioma. BMC Cancer. (2022) 22:900. doi: 10.1186/s12885-022-10001-y 35982398 PMC9386956

[20] Wolf A Agnihotri S Micallef J Mukherjee J Sabha N Cairns R . Hexokinase 2 is a key mediator of aerobic glycolysis and promotes tumor growth in human glioblastoma multiforme. J Exp Med. (2011) 208:313–26. doi: 10.1083/jcb1922oia1 PMC3039857 21242296

[21] Blakeway D . Investigating HK2 as a potential therapeutic target in glioblastoma. In: Wolverhampton (2019). Available online at: https://wlv.openrepository.com/items/c7c851eb-2af5-4f7d-abf2-d7d024e0dfb8.

[22] Stanke KM Wilson C Kidambi S . High expression of glycolytic genes in clinical glioblastoma patients correlates with lower survival. Front Mol Biosci. (2021) 8:752404. doi: 10.3389/fmolb.2021.752404 35004842 PMC8740031

[23] Wolf A Agnihotri S Munoz D Guha A . Developmental profile and regulation of the glycolytic enzyme hexokinase 2 in normal brain and glioblastoma multiforme. Neurobiol Dis. (2011) 44:84–91. doi: 10.1016/j.nbd.2011.06.007 21726646

[24] Blakeway D Karakoula K Morris M Rowther F Eagles L Darling J . Overexpression of Hexokinase 2 is epigenetically regulated by frequent hypomethylation in glioblastoma multiforme. Neuro-Oncology. (2018) 20:i12–i. doi: 10.1093/neuonc/nox238.053

[25] Sanzey M Abdul Rahim SA Oudin A Dirkse A Kaoma T Vallar L . Comprehensive analysis of glycolytic enzymes as therapeutic targets in the treatment of glioblastoma. PloS One. (2015) 10:e0123544. doi: 10.1371/journal.pone.0123544 25932951 PMC4416792

[26] Vartanian A Agnihotri S Wilson MR Burrell KE Tonge PD Alamsahebpour A . Targeting hexokinase 2 enhances response to radio-chemotherapy in glioblastoma. Oncotarget. (2016) 7:69518–35. doi: 10.18632/oncotarget.11680 27588472 PMC5342495

[27] Liu H Liu N Cheng Y Jin W Zhang P Wang X . Hexokinase 2 (HK2), the tumor promoter in glioma, is downregulated by miR-218/Bmi1 pathway. PloS One. (2017) 12:e0189353. doi: 10.1371/journal.pone.0189353 29220380 PMC5722312

[28] Lewandowicz GM Harding B Harkness W Hayward R Thomas DGT Darling JL . Chemosensitivity in childhood brain tumours *in vitro*: evidence of differential sensitivity to lomustine (CCNU) and vincristine. Eur J Cancer. (2000) 36:1955–64. doi: 10.1016/s0959-8049(00)00245-8 11000577

[29] Potter NE Phipps K Harkness W Hayward R Thompson D Jacques TS . Astrocytoma derived short-term cell cultures retain molecular signatures characteristic of the tumour in situ. Exp Cell Res. (2009) 315:2835–46. doi: 10.1016/j.yexcr.2009.06.003 19523942

[30] Kim D Paggi JM Park C Bennett C Salzberg SL . Graph-based genome alignment and genotyping with HISAT2 and HISAT-genotype. Nat Biotechnol. (2019) 37:907–15. doi: 10.1038/s41587-019-0201-4 31375807 PMC7605509

[31] Pertea M Pertea GM Antonescu CM Chang TC Mendell JT Salzberg SL . StringTie enables improved reconstruction of a transcriptome from RNA-seq reads. Nat Biotechnol. (2015) 33:290–5. doi: 10.1038/nbt.3122 25690850 PMC4643835

[32] Love MI Huber W Anders S . Moderated estimation of fold change and dispersion for RNA-seq data with DESeq2. Genome Biol. (2014) 15:550. doi: 10.1186/s13059-014-0550-8 25516281 PMC4302049

[33] Szklarczyk D Kirsch R Koutrouli M Nastou K Mehryary F Hachilif R . The STRING database in 2023: protein-protein association networks and functional enrichment analyses for any sequenced genome of interest. Nucleic Acids Res. (2023) 51:D638–46. doi: 10.1093/nar/gkac1000 36370105 PMC9825434

[34] Brohée S van Helden J . Evaluation of clustering algorithms for protein-protein interaction networks. BMC Bioinf. (2006) 7:488. doi: 10.1186/1471-2105-7-488 PMC1637120 17087821

[35] Noman AA Saba AA Sayem M Yasmin T Nabi A . Identification of potential hub genes and molecular mechanisms in breast cancer microenvironment: A comprehensive transcriptomics approach. Med (Baltimore). (2025) 104:e44142. doi: 10.1097/md.0000000000044142 40898538 PMC12401218

[36] Ashburner M Ball CA Blake JA Botstein D Butler H Cherry JM . Gene Ontology: tool for the unification of biology. Nat Genet. (2000) 25:25–9. doi: 10.1038/75556 10802651 PMC3037419

[37] GeneOntologyConsortium . The Gene Ontology knowledgebase in 2023. Genetics. (2023) 224:iyad031. doi: 10.1093/genetics/iyad031 36866529 PMC10158837

[38] Kanehisa M Furumichi M Tanabe M Sato Y Morishima K . KEGG: new perspectives on genomes, pathways, diseases and drugs. Nucleic Acids Res. (2017) 45:D353–61. doi: 10.1093/nar/gkw1092 27899662 PMC5210567

[39] Young MD Wakefield MJ Smyth GK Oshlack A . Gene ontology analysis for RNA-seq: accounting for selection bias. Genome Biol. (2010) 11:R14. doi: 10.1186/gb-2010-11-2-r14 20132535 PMC2872874

[40] GalaxyCommunity . The Galaxy platform for accessible, reproducible and collaborative biomedical analyses: 2022 update. Nucleic Acids Res. (2022) 50:W345–51. doi: 10.1093/nar/gkac247 PMC9252830 35446428

[41] Walter W Sánchez-Cabo F Ricote M . GOplot: an R package for visually combining expression data with functional analysis. Bioinformatics. (2015) 31:2912–4. doi: 10.1093/bioinformatics/btv300 25964631

[42] Merico D Isserlin R Stueker O Emili A Bader GD . Enrichment map: a network-based method for gene-set enrichment visualization and interpretation. PloS One. (2010) 5:e13984. doi: 10.1371/journal.pone.0013984 21085593 PMC2981572

[43] Shannon P Markiel A Ozier O Baliga NS Wang JT Ramage D . Cytoscape: a software environment for integrated models of biomolecular interaction networks. Genome Res. (2003) 13:2498–504. doi: 10.1101/gr.1239303 14597658 PMC403769

[44] Luo W Brouwer C . Pathview: an R/Bioconductor package for pathway-based data integration and visualization. Bioinformatics. (2013) 29:1830–1. doi: 10.1093/bioinformatics/btt285 23740750 PMC3702256

[45] Lamb J Crawford ED Peck D Modell JW Blat IC Wrobel MJ . The Connectivity Map: using gene-expression signatures to connect small molecules, genes, and disease. Science. (2006) 313:1929–35. doi: 10.1126/science.1132939 17008526

[46] Yost SE Pastorino S Rozenzhak S Smith EN Chao YS Jiang P . High-resolution mutational profiling suggests the genetic validity of glioblastoma patient-derived pre-clinical models. PloS One. (2013) 8:e56185. doi: 10.1371/journal.pone.0056185 23441165 PMC3575368

[47] Agilent . XFp Extracellular Flux Analyser User Guide 2015. Available online at: https://www.agilent.com/cs/pubimages/misc/XFp_Extracellular_Flux_Analyzer_User_Guide.pdf (Accessed September 15, 2022).

[48] Agilent . Seahorse XFp Glycolytic Stress Test User Guide 2019. Available online at: https://www.agilent.com/cs/library/usermanuals/public/XFp_Glycolysis_Stress_Test_User_Guide%20Rev_D.pdf (Accessed September 15, 2022).

[49] Xiao H Li S Zhang D Liu T Yu M Wang F . Separate and concurrent use of 2-deoxy-D-glucose and 3-bromopyruvate in pancreatic cancer cells. Oncol Rep. (2013) 29:329–34. doi: 10.3892/or.2012.2085 23076497

[50] Pajak B Siwiak E Sołtyka M Priebe A Zieliński R Fokt I . 2-Deoxy-d-Glucose and its analogs: from diagnostic to therapeutic agents. Int J Mol Sci. (2019) 21:234. doi: 10.3390/ijms21010234 31905745 PMC6982256

[51] Ben-Yoseph O Lyons JC Song CW Ross BD . Mechanism of action of lonidamine in the 9L brain tumor model involves inhibition of lactate efflux and intracellular acidification. J Neuro-Oncol. (1998) 36:149–57. doi: 10.1023/a:1005819604858 9525814

[52] Raez LE Papadopoulos K Ricart AD Chiorean EG Dipaola RS Stein MN . A phase I dose-escalation trial of 2-deoxy-D-glucose alone or combined with docetaxel in patients with advanced solid tumors. Cancer Chemother Pharmacol. (2013) 71:523–30. doi: 10.1007/s00280-012-2045-1 23228990

[53] Kunjithapatham R Geschwind JF Rao PP Boronina TN Cole RN Ganapathy-Kanniappan S . Systemic administration of 3-bromopyruvate reveals its interaction with serum proteins in a rat model. BMC Res Notes. (2013) 6:277. doi: 10.1186/1756-0500-6-277 23866825 PMC3728150

[54] Azevedo-Silva J Queirós O Baltazar F Ułaszewski S Goffeau A Ko Y . The anticancer agent 3-bromopyruvate: a simple but powerful molecule taken from the lab to the bedside. J Bioenerg Biomembr. (2016) 48:349–62. doi: 10.1007/s10863-016-9670-z 27457582

[55] Nancolas B Guo L Zhou R Nath K Nelson DS Leeper DB . The anti-tumour agent lonidamine is a potent inhibitor of the mitochondrial pyruvate carrier and plasma membrane monocarboxylate transporters. Biochem J. (2016) 473:929–36. doi: 10.1042/bj20151120 26831515 PMC4814305

[56] Yadav S Pandey SK Kumar A Kujur PK Singh RP Singh SM . Antitumor and chemosensitizing action of 3-bromopyruvate: implication of deregulated metabolism. Chem Biol Interact. (2017) 270:73–89. doi: 10.1016/j.cbi.2017.04.015 28433571

[57] Garcia SN Guedes RC Marques MM . Unlocking the potential of HK2 in cancer metabolism and therapeutics. Curr Med Chem. (2019) 26:7285–322. doi: 10.2174/0929867326666181213092652 30543165

[58] Zhang Y Li Q Huang Z Li B Nice EC Huang C . Targeting glucose metabolism enzymes in cancer treatment: current and emerging strategies. Cancers (Basel). (2022) 14:4568. doi: 10.3390/cancers14194568 36230492 PMC9559313

[59] Fang M Richardson B Cameron CM Dazard J-E Cameron MJ . Drug perturbation gene set enrichment analysis (dpGSEA): a new transcriptomic drug screening approach. BMC Bioinf. (2021) 22:22. doi: 10.1186/s12859-020-03929-0 33435872 PMC7805197

[60] Pruteanu L-L Bender A . Using transcriptomics and cell morphology data in drug discovery: the long road to practice. ACS Med Chem Lett. (2023) 14:386–95. doi: 10.1021/acsmedchemlett.3c00015 37077392 PMC10107910

[61] Musa A Ghoraie LS Zhang SD Glazko G Yli-Harja O Dehmer M . A review of connectivity map and computational approaches in pharmacogenomics. Brief Bioinform. (2018) 19:506–23. doi: 10.1093/bib/bbx023 28069634 PMC5952941

[62] Saga I Shibao S Okubo J Osuka S Kobayashi Y Yamada S . Integrated analysis identifies different metabolic signatures for tumor-initiating cells in a murine glioblastoma model. Neuro Oncol. (2014) 16:1048–56. doi: 10.1093/neuonc/nou096 24860177 PMC4096179

[63] Hoang-Minh LB Siebzehnrubl FA Yang C Suzuki-Hatano S Dajac K Loche T . Infiltrative and drug-resistant slow-cycling cells support metabolic heterogeneity in glioblastoma. EMBO J. (2018) 37. doi: 10.15252/embj.201798772 30322894 PMC6276884

[64] Garofano L Migliozzi S Oh YT D'Angelo F Najac RD Ko A . Pathway-based classification of glioblastoma uncovers a mitochondrial subtype with therapeutic vulnerabilities. Nat Cancer. (2021) 2:141–56. doi: 10.1038/s43018-020-00159-4 33681822 PMC7935068

[65] Seliger C Meyer AL Leidgens V Rauer L Moeckel S Jachnik B . Metabolic heterogeneity of brain tumor cells of proneural and mesenchymal origin. Int J Mol Sci. (2022) 23:11629. doi: 10.3390/ijms231911629 36232951 PMC9569970

[66] Basheer AS Abas F Othman I Naidu R . Role of inflammatory mediators, macrophages, and neutrophils in glioma maintenance and progression: mechanistic understanding and potential therapeutic applications. Cancers (Basel). (2021) 13:4226. doi: 10.3390/cancers13164226 34439380 PMC8393628

[67] Șovrea AS Boșca B Melincovici CS Constantin AM Crintea A Mărginean M . Multiple faces of the glioblastoma microenvironment. Int J Mol Sci. (2022) 23:595. doi: 10.3390/ijms23020595 35054779 PMC8775531

[68] Han M Sun Y Zhao W Xiang G Wang X Jiang Z . Comprehensive characterization of TNFSF14/LIGHT with implications in prognosis and immunotherapy of human gliomas. Front Immunol. (2022) 13:1025286. doi: 10.3389/fimmu.2022.1025286 36341396 PMC9632349

[69] Yeo ECF Brown MP Gargett T Ebert LM . The role of cytokines and chemokines in shaping the immune microenvironment of glioblastoma: implications for immunotherapy. Cells. (2021) 10:607. doi: 10.3390/cells10030607 33803414 PMC8001644

[70] Yang Y Lv W Xu S Shi F Shan A Wang J . Molecular and clinical characterization of LIGHT/TNFSF14 expression at transcriptional level via 998 samples with brain glioma. Front Mol Biosci. (2021) 8:567327. doi: 10.3389/fmolb.2021.567327 34513918 PMC8430338

[71] Kast RE Hill QA Wion D Mellstedt H Focosi D Karpel-Massler G . Glioblastoma-synthesized G-CSF and GM-CSF contribute to growth and immunosuppression: potential therapeutic benefit from dapsone, fenofibrate, and ribavirin. Tumour Biol. (2017) 39:1010428317699797. doi: 10.1177/1010428317699797 28459367

[72] Revoltella RP Menicagli M Campani D . Granulocyte–macrophage colony-stimulating factor as an autocrine survival-growth factor in human gliomas. Cytokine. (2012) 57:347–59. doi: 10.1016/j.cyto.2011.11.016 22200506

[73] Kartikasari AER Huertas CS Mitchell A Plebanski M . Tumor-induced inflammatory cytokines and the emerging diagnostic devices for cancer detection and prognosis. Front Oncol. (2021) 11:692142. doi: 10.3389/fonc.2021.692142 34307156 PMC8294036

[74] Ham B Fernandez MC D'Costa Z Brodt P . The diverse roles of the TNF axis in cancer progression and metastasis. Trends Cancer Res. (2016) 11:1–27. 27928197 PMC5138060

[75] Raza S Rajak S Tewari A Gupta P Chattopadhyay N Sinha RA . Multifaceted role of chemokines in solid tumors: from biology to therapy. Semin Cancer Biol. (2022) 86:1105–21. doi: 10.1016/j.semcancer.2021.12.011 34979274 PMC7613720

[76] Abdul-Rahman T Ghosh S Badar SM Nazir A Bamigbade GB Aji N . The paradoxical role of cytokines and chemokines at the tumor microenvironment: a comprehensive review. Eur J Med Res. (2024) 29:124. doi: 10.1186/s40001-024-01711-z 38360737 PMC10868116

[77] Li H Wu M Zhao X . Role of chemokine systems in cancer and inflammatory diseases. MedComm (2020). (2022) 3:e147. doi: 10.1002/mco2.147 35702353 PMC9175564

[78] Mi Y Guo N Luan J Cheng J Hu Z Jiang P . The emerging role of myeloid-derived suppressor cells in the glioma immune suppressive microenvironment. Front Immunol. (2020) 11:737. doi: 10.3389/fimmu.2020.00737 32391020 PMC7193311

[79] Veglia F Sanseviero E Gabrilovich DI . Myeloid-derived suppressor cells in the era of increasing myeloid cell diversity. Nat Rev Immunol. (2021) 21:485–98. doi: 10.1038/s41577-020-00490-y 33526920 PMC7849958

[80] Hu J Zhao Q Kong LY Wang J Yan J Xia X . Regulation of tumor immune suppression and cancer cell survival by CXCL1/2 elevation in glioblastoma multiforme. Sci Adv. (2021) 7. doi: 10.1126/sciadv.abc2511 33571109 PMC7840139

[81] De Leo A Ugolini A Veglia F . Myeloid cells in glioblastoma microenvironment. Cells [Internet]. (2021) 10:18. doi: 10.3390/cells10010018 33374253 PMC7824606

[82] Kohli K Pillarisetty VG Kim TS . Key chemokines direct migration of immune cells in solid tumors. Cancer Gene Ther. (2022) 29:10–21. doi: 10.1038/s41417-021-00303-x 33603130 PMC8761573

[83] Wang J Li S Lan Y Liu X Li W . Glioma-associated macrophages: unraveling their dual role in the microenvironment and therapeutic implications. Curr Med. (2024) 3:4. doi: 10.1007/s44194-024-00031-y 30311153

[84] Lindau D Gielen P Kroesen M Wesseling P Adema GJ . The immunosuppressive tumour network: myeloid-derived suppressor cells, regulatory T cells and natural killer T cells. Immunology. (2013) 138:105–15. doi: 10.1111/imm.12036 23216602 PMC3575763

[85] Kamran N Kadiyala P Saxena M Candolfi M Li Y Moreno-Ayala MA . Immunosuppressive myeloid cells' blockade in the glioma microenvironment enhances the efficacy of immune-stimulatory gene therapy. Mol Ther. (2017) 25:232–48. doi: 10.1016/j.ymthe.2016.10.003 28129117 PMC5363306

[86] González-Navajas JM Fan DD Yang S Yang FM Lozano-Ruiz B Shen L . The impact of Tregs on the anticancer immunity and the efficacy of immune checkpoint inhibitor therapies. Front Immunol. (2021) 12:625783. doi: 10.3389/fimmu.2021.625783 33717139 PMC7952426

[87] Bausart M Préat V Malfanti A . Immunotherapy for glioblastoma: the promise of combination strategies. J Exp Clin Cancer Res. (2022) 41:35. doi: 10.1186/s13046-022-02251-2 35078492 PMC8787896

[88] Qin D Zhang Y Shu P Lei Y Li X Wang Y . Targeting tumor-infiltrating tregs for improved antitumor responses. Front Immunol. (2024) 15:1325946. doi: 10.3389/fimmu.2024.1325946 38500876 PMC10944859

[89] Zhao H Wu L Yan G Chen Y Zhou M Wu Y . Inflammation and tumor progression: signaling pathways and targeted intervention. Signal Transduction Targeted Ther. (2021) 6:263. doi: 10.1038/s41392-021-00658-5 34248142 PMC8273155

[90] Liu X Yin L Shen S Hou Y . Inflammation and cancer: paradoxical roles in tumorigenesis and implications in immunotherapies. Genes Dis. (2023) 10:151–64. doi: 10.1016/j.gendis.2021.09.006 37013041 PMC10066281

[91] Demir E Rahmanipour E Ghorbani M Gupta K Zeinali M Karsy M . Neutrophils in glioblastoma: orchestrators of the tumor microenvironment and immune evasion. Mol Biol Rep. (2026) 53:349. doi: 10.1007/s11033-026-11525-3 41632336 PMC12868103

[92] Verhaak RG Hoadley KA Purdom E Wang V Qi Y Wilkerson MD . Integrated genomic analysis identifies clinically relevant subtypes of glioblastoma characterized by abnormalities in PDGFRA, IDH1, EGFR, and NF1. Cancer Cell. (2010) 17:98–110. doi: 10.1016/j.ccr.2009.12.020 20129251 PMC2818769

[93] Wang Q Hu B Hu X Kim H Squatrito M Scarpace L . Tumor evolution of glioma-intrinsic gene expression subtypes associates with immunological changes in the microenvironment. Cancer Cell. (2017) 32:42–56.e6. doi: 10.1016/j.ccell.2017.06.003 28697342 PMC5599156

[94] Neftel C Laffy J Filbin MG Hara T Shore ME Rahme GJ . An integrative model of cellular states, plasticity, and genetics for glioblastoma. Cell. (2019) 178:835–849.e21. doi: 10.1016/j.cell.2019.06.024 31327527 PMC6703186

[95] Zanotto-Filho A Gonçalves RM Klafke K de Souza PO Dillenburg FC Carro L . Inflammatory landscape of human brain tumors reveals an NFκB dependent cytokine pathway associated with mesenchymal glioblastoma. Cancer Lett. (2017) 390:176–87. doi: 10.1016/j.canlet.2016.12.015 28007636

[96] Kaffes I Szulzewsky F Chen Z Herting CJ Gabanic B Velázquez Vega JE . Human mesenchymal glioblastomas are characterized by an increased immune cell presence compared to proneural and classical tumors. Oncoimmunology. (2019) 8:e1655360. doi: 10.1080/2162402x.2019.1655360 31646100 PMC6791439

[97] Xu C Hou P Li X Xiao M Zhang Z Li Z . Comprehensive understanding of glioblastoma molecular phenotypes: classification, characteristics, and transition. Cancer Biol Med. (2024) 21:363–81. doi: 10.20892/j.issn.2095-3941.2023.0510 38712813 PMC11131044

[98] Chen J Li G Sun D Li H Chen L . Research progress of hexokinase 2 in inflammatory-related diseases and its inhibitors. Eur J Med Chem. (2024) 264:115986. doi: 10.1016/j.ejmech.2023.115986 38011767

[99] Guo D Tong Y Jiang X Meng Y Jiang H Du L . Aerobic glycolysis promotes tumor immune evasion by hexokinase2-mediated phosphorylation of IκBα. Cell Metab. (2022) 34:1312–1324.e6. doi: 10.1016/j.cmet.2022.08.002 36007522

[100] Lin J Fang W Xiang Z Wang Q Cheng H Chen S . Glycolytic enzyme HK2 promotes PD-L1 expression and breast cancer cell immune evasion. Front Immunol. (2023) 14:1189953. doi: 10.3389/fimmu.2023.1189953 37377974 PMC10291184

[101] Liu T Zhang L Joo D Sun S-C . NF-κB signaling in inflammation. Signal Transduction Targeted Ther. (2017) 2:17023. doi: 10.1038/sigtrans.2017.23 PMC5661633 29158945

[102] Hibino S Kawazoe T Kasahara H Itoh S Ishimoto T Sakata-Yanagimoto M . Inflammation-induced tumorigenesis and metastasis. Int J Mol Sci. (2021) 22. doi: 10.3390/ijms22115421 34063828 PMC8196678

[103] Keller CW Münz C Lünemann JD . Autophagy pathways in CNS myeloid cell immune functions. Trends Neurosci. (2020) 43:1024–33. doi: 10.1016/j.tins.2020.09.003 33010946

[104] de Torre-Minguela C Mesa Del Castillo P Pelegrín P . The NLRP3 and pyrin inflammasomes: Implications in the pathophysiology of autoinflammatory diseases. Front Immunol. (2017) 8:43. doi: 10.3389/fimmu.2017.00043 28191008 PMC5271383

[105] Olona A Leishman S Anand PK . The NLRP3 inflammasome: Regulation by metabolic signals. Trends Immunol. (2022) 43:978–89. doi: 10.1016/j.it.2022.10.003 36371361

[106] Baik SH Ramanujan VK Becker C Fett S Underhill DM Wolf AJ . Hexokinase dissociation from mitochondria promotes oligomerization of VDAC that facilitates NLRP3 inflammasome assembly and activation. Sci Immunol. (2023) 8:eade7652. doi: 10.1126/sciimmunol.ade7652 37327321 PMC10360408

[107] Guarnaccia L Navone SE Trombetta E Cordiglieri C Cherubini A Crisà FM . Angiogenesis in human brain tumors: Screening of drug response through a patient-specific cell platform for personalized therapy. Sci Rep. (2018) 8:8748. doi: 10.1038/s41598-018-27116-7 29884885 PMC5993734

[108] Ahir BK Engelhard HH Lakka SS . Tumor development and angiogenesis in adult brain tumor: Glioblastoma. Mol Neurobiol. (2020) 57:2461–78. doi: 10.1007/s12035-020-01892-8 32152825 PMC7170819

[109] Lai WK Adams DH . Angiogenesis and chronic inflammation; the potential for novel therapeutic approaches in chronic liver disease. J Hepatol. (2005) 42:7–11. doi: 10.1016/j.jhep.2004.11.008 15629498

[110] Zhou W Yang L Nie L Lin H . Unraveling the molecular mechanisms between inflammation and tumor angiogenesis. Am J Cancer Res. (2021) 11:301–17. PMC7868762 33575073

[111] Ebeling S Kowalczyk A Perez-Vazquez D Mattiola I . Regulation of tumor angiogenesis by the crosstalk between innate immunity and endothelial cells. Front Oncol. (2023) 13:1171794. doi: 10.3389/fonc.2023.1171794 37234993 PMC10206118

[112] Jiang X Wang J Deng X Xiong F Zhang S Gong Z . The role of microenvironment in tumor angiogenesis. J Exp Clin Cancer Res. (2020) 39:204. doi: 10.1186/s13046-020-01709-5 32993787 PMC7526376

[113] Geindreau M Bruchard M Vegran F . Role of cytokines and chemokines in angiogenesis in a tumor context. Cancers (Basel). (2022) 14:2446. doi: 10.3390/cancers14102446 35626056 PMC9139472

[114] Kumar A Taghi Khani A Sanchez Ortiz A Swaminathan S . GM-CSF: A double-edged sword in cancer immunotherapy. Front Immunol. (2022) 13:901277. doi: 10.3389/fimmu.2022.901277 35865534 PMC9294178

[115] Tazzyman S Lewis CE Murdoch C . Neutrophils: Key mediators of tumour angiogenesis. Int J Exp Pathol. (2009) 90:222–31. doi: 10.1111/j.1365-2613.2009.00641.x 19563607 PMC2697547

[116] Xiong X Liao X Qiu S Xu H Zhang S Wang S . CXCL8 in tumor biology and its implications for clinical translation. Front Mol Biosci. (2022) 9:723846. doi: 10.3389/fmolb.2022.723846 35372515 PMC8965068

[117] Ozel I Duerig I Domnich M Lang S Pylaeva E Jablonska J . The good, the bad, and the ugly: Neutrophils, angiogenesis, and cancer. Cancers (Basel). (2022) 14:536. doi: 10.3390/cancers14030536 35158807 PMC8833332

[118] Larráyoz IM Martínez-Herrero S García-Sanmartín J Ochoa-Callejero L Martínez A . Adrenomedullin and tumour microenvironment. J Transl Med. (2014) 12:339. doi: 10.1186/s12967-014-0339-2 25475159 PMC4272513

[119] Chi AS Sorensen AG Jain RK Batchelor TT . Angiogenesis as a therapeutic target in malignant gliomas. Oncologist. (2009) 14:621–36. doi: 10.1634/theoncologist.2008-0272 19487335 PMC4790121

[120] Agnihotri S Mansouri S Burrell K Li M Mamatjan Y Liu J . Ketoconazole and posaconazole selectively target HK2-expressing glioblastoma cells. Clin Cancer Res. (2019) 25:844–55. doi: 10.1158/1078-0432.ccr-18-1854 30322879 PMC8103287

[121] Fu M Zhou Z Huang X Chen Z Zhang L Zhang J . Use of bevacizumab in recurrent glioblastoma: A scoping review and evidence map. BMC Cancer. (2023) 23:544. doi: 10.1186/s12885-023-11043-6 37316802 PMC10265794

[122] Pollard SM Yoshikawa K Clarke ID Danovi D Stricker S Russell R . Glioma stem cell lines expanded in adherent culture have tumor-specific phenotypes and are suitable for chemical and genetic screens. Cell Stem Cell. (2009) 4:568–80. doi: 10.1016/j.stem.2009.03.014 19497285

[123] Loilome W Joshi AD ap Rhys CM Piccirillo S Vescovi AL Gallia GL . Glioblastoma cell growth is suppressed by disruption of fibroblast growth factor pathway signaling. J Neuro-Oncol. (2009) 94:359–66. doi: 10.1007/s11060-009-9885-5 19340397

[124] Gouazé-Andersson V Delmas C Taurand M Martinez-Gala J Evrard S Mazoyer S . FGFR1 induces glioblastoma radioresistance through the PLCγ/Hif1α pathway. Cancer Res. (2016) 76:3036–44. doi: 10.1158/0008-5472.can-15-2058 26896280

[125] Jimenez-Pascual A Mitchell K Siebzehnrubl FA Lathia JD . FGF2: A novel druggable target for glioblastoma? Expert Opin Ther Targets. (2020) 24:311–8. doi: 10.1080/14728222.2020.1736558 32174197 PMC9075824

[126] Alshahrany N Begum A Siebzehnrubl D Jimenez-Pascual A Siebzehnrubl FA . Spatial distribution and functional relevance of FGFR1 and FGFR2 expression for glioblastoma tumor invasion. Cancer Lett. (2023) 571:216349. doi: 10.1016/j.canlet.2023.216349 37579831 PMC10840508

[127] Elfenbein A Simons M . Syndecan-4 signaling at a glance. J Cell Sci. (2013) 126:3799–804. doi: 10.1242/jcs.124636 23970415 PMC3757327

[128] Deryugina EI Quigley JP . Pleiotropic roles of matrix metalloproteinases in tumor angiogenesis: Contrasting, overlapping and compensatory functions. Biochim Biophys Acta. (2010) 1803:103–20. doi: 10.1016/j.bbamcr.2009.09.017 19800930 PMC2824055

[129] Quintero-Fabián S Arreola R Becerril-Villanueva E Torres-Romero JC Arana-Argáez V Lara-Riegos J . Role of matrix metalloproteinases in angiogenesis and cancer. Front Oncol. (2019) 9:1370. doi: 10.3389/fonc.2019.01370 31921634 PMC6915110

[130] Choe G Park JK Jouben-Steele L Kremen TJ Liau LM Vinters HV . Active matrix metalloproteinase 9 expression is associated with primary glioblastoma subtype1. Clin Cancer Res. (2002) 8:2894–901. 12231534

[131] Nakada M Okada Y Yamashita J . The role of matrix metalloproteinases in glioma invasion. Front Biosci. (2003) 8:e261-9. doi: 10.2741/1016 12456313

[132] Yu C-F Chen F-H Lu M-H Hong J-H Chiang C-S . Dual roles of tumour cells-derived matrix metalloproteinase 2 on brain tumour growth and invasion. Br J Cancer. (2017) 117:1828–36. doi: 10.1038/bjc.2017.362 29065106 PMC5729475

[133] Zhou W Yu X Sun S Zhang X Yang W Zhang J . Increased expression of MMP-2 and MMP-9 indicates poor prognosis in glioma recurrence. Biomedicine Pharmacotherapy. (2019) 118:109369. doi: 10.1016/j.biopha.2019.109369 31545229

[134] Koh MY Powis G . Passing the baton: the HIF switch. Trends Biochem Sci. (2012) 37:364–72. doi: 10.1016/j.tibs.2012.06.004 PMC3433036 22818162

[135] Tong W-W Tong G-H Chen X-X Zheng H-C Wang Y-Z . HIF2α is associated with poor prognosis and affects the expression levels of survivin and cyclin D1 in gastric carcinoma. Int J Oncol. (2015) 46:233–42. doi: 10.3892/ijo.2014.2719 25338835

[136] Befani C Liakos P . The role of hypoxia-inducible factor-2 alpha in angiogenesis. J Cell Physiol. (2018) 233:9087–98. doi: 10.1002/jcp.26805 29968905

[137] Palombo R Caporali S Falconi M Iacovelli F Morozzo Della Rocca B Lo Surdo A . Luteolin-7-O-β-d-Glucoside Inhibits Cellular Energy Production Interacting with HEK2 in Keratinocytes. Int J Mol Sci. (2019) 20:2689. doi: 10.3390/ijms20112689 31159225 PMC6600217

[138] Korga A Ostrowska M Jozefczyk A Iwan M Wojcik R Zgorka G . Apigenin and hesperidin augment the toxic effect of doxorubicin against HepG2 cells. BMC Pharmacol Toxicol. (2019) 20:22. doi: 10.1186/s40360-019-0301-2 31053173 PMC6499973

[139] Zhou L Li M Yu X Gao F Li W . Repression of hexokinases II-mediated glycolysis contributes to piperlongumine-induced tumor suppression in non-small cell lung cancer cells. Int J Biol Sci. (2019) 15:826–37. doi: 10.7150/ijbs.31749 30906213 PMC6429016

[140] Kisselev AF Goldberg AL . Proteasome inhibitors: From research tools to drug candidates. Chem Biol. (2001) 8:739–58. doi: 10.1016/s1074-5521(01)00056-4 11514224

[141] Zhong J Lu S Jia X Li Q Liu L Xie P . Role of endoplasmic reticulum stress in apoptosis induced by HK2 inhibitor and its potential as a new drug combination strategy. Cell Stress Chaperones. (2022) 27:273–83. doi: 10.1007/s12192-022-01267-z 35355227 PMC9106785

[142] Jalil AT Abdulhadi MA Alkubaisy SA Thejeel SH Essa IM Merza MS . The role of endoplasmic reticulum stress in promoting aerobic glycolysis in cancer cells: An overview. Pathol Res Pract. (2023) 251:154905. doi: 10.1016/j.prp.2023.154905 37925820

[143] Xu Y Fang D . Endoplasmic reticulum-associated degradation and beyond: The multitasking roles for HRD1 in immune regulation and autoimmunity. J Autoimmun. (2020) 109:102423. doi: 10.1016/j.jaut.2020.102423 32057541 PMC9434712

[144] Lim PJ Danner R Liang J Doong H Harman C Srinivasan D . Ubiquilin and p97/VCP bind erasin, forming a complex involved in ERAD. J Cell Biol. (2009) 187:201–17. doi: 10.1083/jcb.200903024 19822669 PMC2768832

[145] El Ayadi A Stieren ES Barral JM Boehning D . Ubiquilin-1 and protein quality control in Alzheimer disease. Prion. (2013) 7:164–9. doi: 10.4161/pri.23711 23360761 PMC3609125

[146] Lee JJ Park JK Jeong J Jeon H Yoon JB Kim EE . Complex of Fas-associated factor 1 (FAF1) with valosin-containing protein (VCP)-Npl4-Ufd1 and polyubiquitinated proteins promotes endoplasmic reticulum-associated degradation (ERAD). J Biol Chem. (2013) 288:6998–7011. doi: 10.1074/jbc.m112.417576 23293021 PMC3591610

[147] Richly H Rape M Braun S Rumpf S Hoege C Jentsch S . A series of ubiquitin binding factors connects CDC48/p97 to substrate multiubiquitylation and proteasomal targeting. Cell. (2005) 120:73–84. doi: 10.1016/j.cell.2004.11.013 15652483

