# Supplementary material for: HK2 knockdown is associated with context-dependent inflammatory and angiogenesis-related transcriptional programmes in glioblastoma cells
Source: Front Immunol. 2026 Jun 16;17:1842240. doi: 10.3389/fimmu.2026.1842240 (PMC13316742; doi:10.3389/fimmu.2026.1842240)
Supplement: Supplementary file 1 [file Presentation1.pptx]

## Slide 1
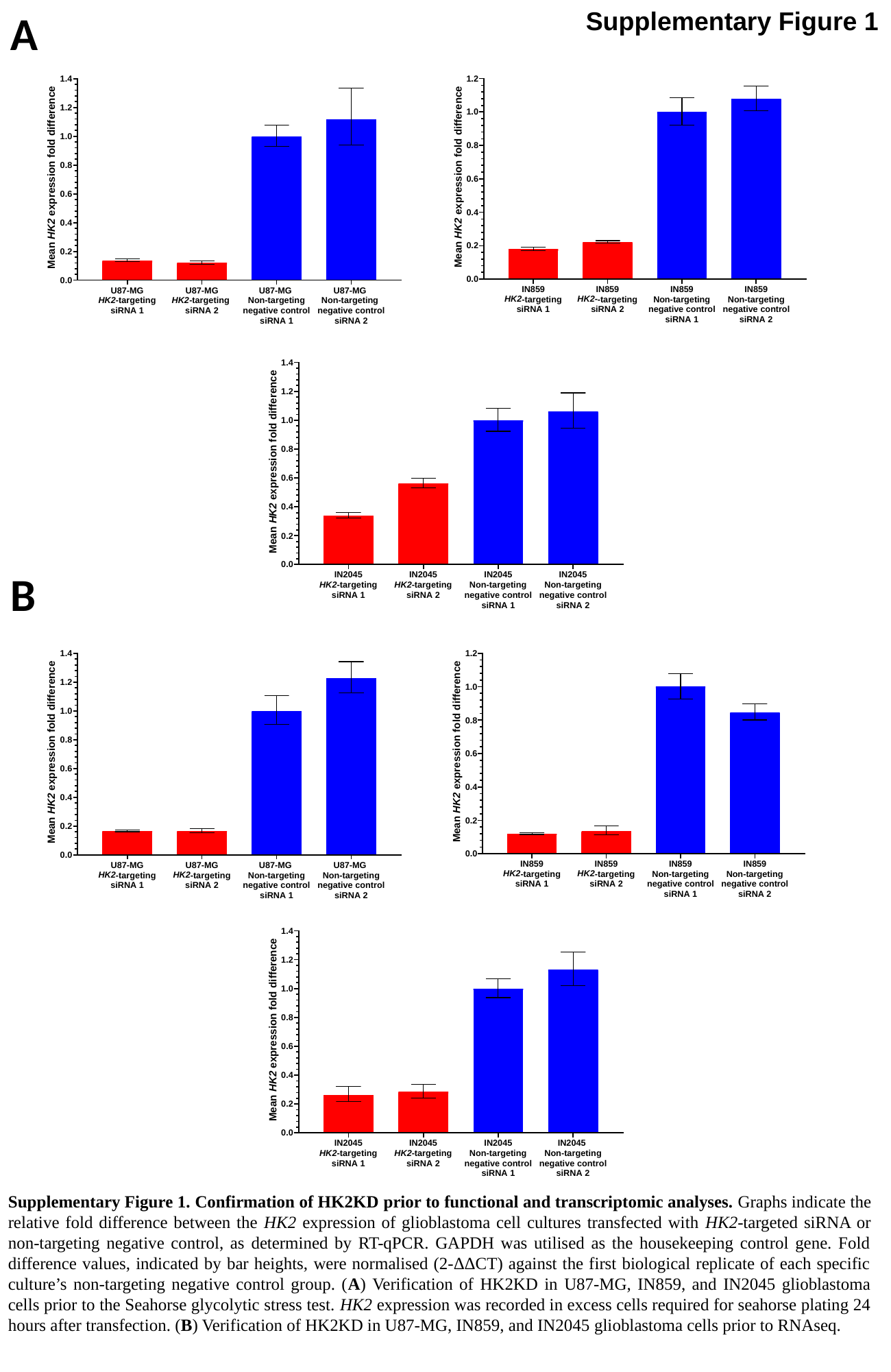

A
Supplementary Figure 1
B
Supplementary Figure 1. Confirmation of HK2KD prior to functional and transcriptomic analyses. Graphs indicate the relative fold difference between the HK2 expression of glioblastoma cell cultures transfected with HK2-targeted siRNA or non-targeting negative control, as determined by RT-qPCR. GAPDH was utilised as the housekeeping control gene. Fold difference values, indicated by bar heights, were normalised (2-ΔΔCT) against the first biological replicate of each specific culture’s non-targeting negative control group. (A) Verification of HK2KD in U87-MG, IN859, and IN2045 glioblastoma cells prior to the Seahorse glycolytic stress test. HK2 expression was recorded in excess cells required for seahorse plating 24 hours after transfection. (B) Verification of HK2KD in U87-MG, IN859, and IN2045 glioblastoma cells prior to RNAseq.

## Slide 2
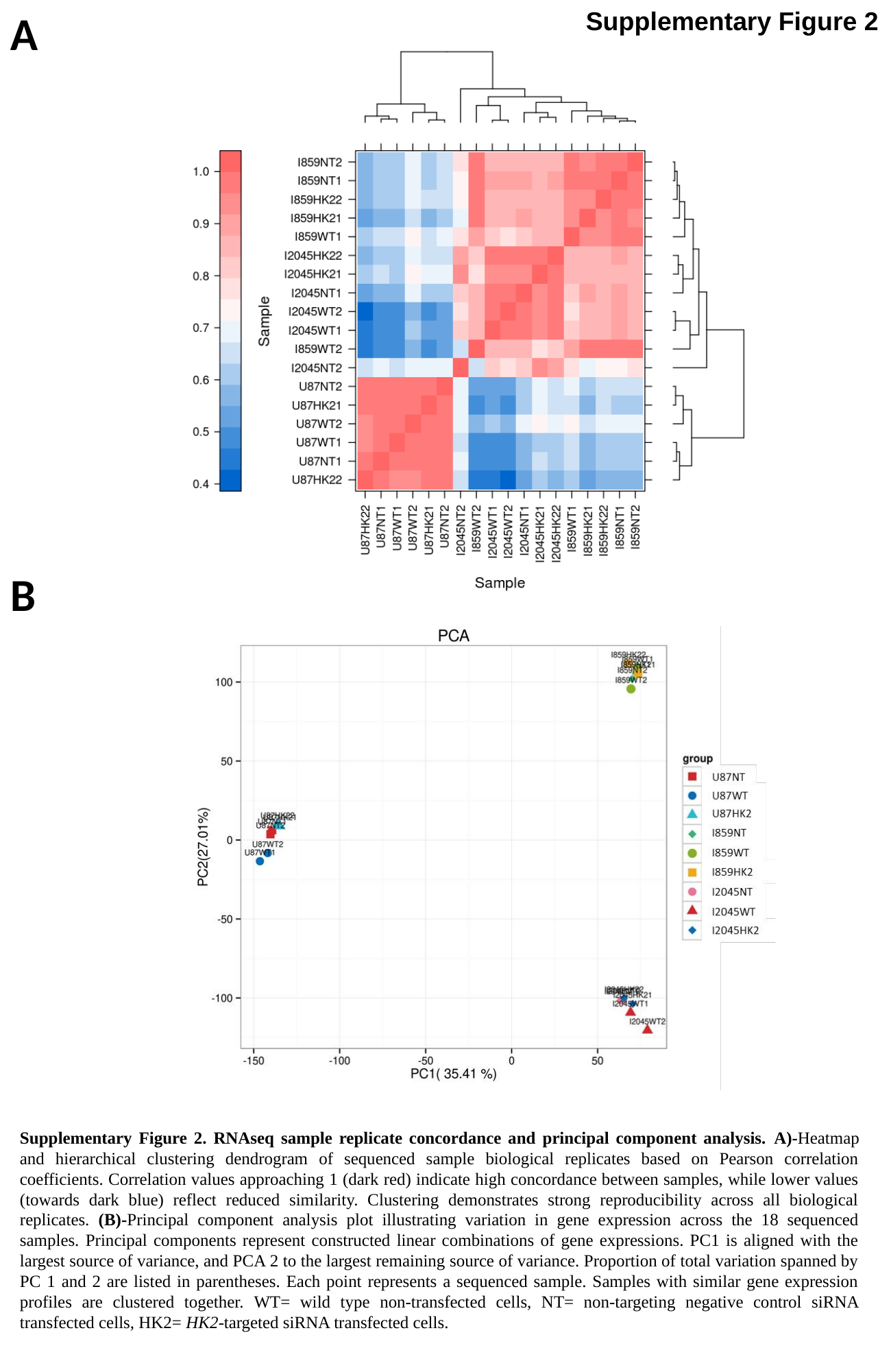

A
Supplementary Figure 2
B
Supplementary Figure 2. RNAseq sample replicate concordance and principal component analysis. A)-Heatmap and hierarchical clustering dendrogram of sequenced sample biological replicates based on Pearson correlation coefficients. Correlation values approaching 1 (dark red) indicate high concordance between samples, while lower values (towards dark blue) reflect reduced similarity. Clustering demonstrates strong reproducibility across all biological replicates. (B)-Principal component analysis plot illustrating variation in gene expression across the 18 sequenced samples. Principal components represent constructed linear combinations of gene expressions. PC1 is aligned with the largest source of variance, and PCA 2 to the largest remaining source of variance. Proportion of total variation spanned by PC 1 and 2 are listed in parentheses. Each point represents a sequenced sample. Samples with similar gene expression profiles are clustered together. WT= wild type non-transfected cells, NT= non-targeting negative control siRNA transfected cells, HK2= HK2-targeted siRNA transfected cells.

## Slide 3
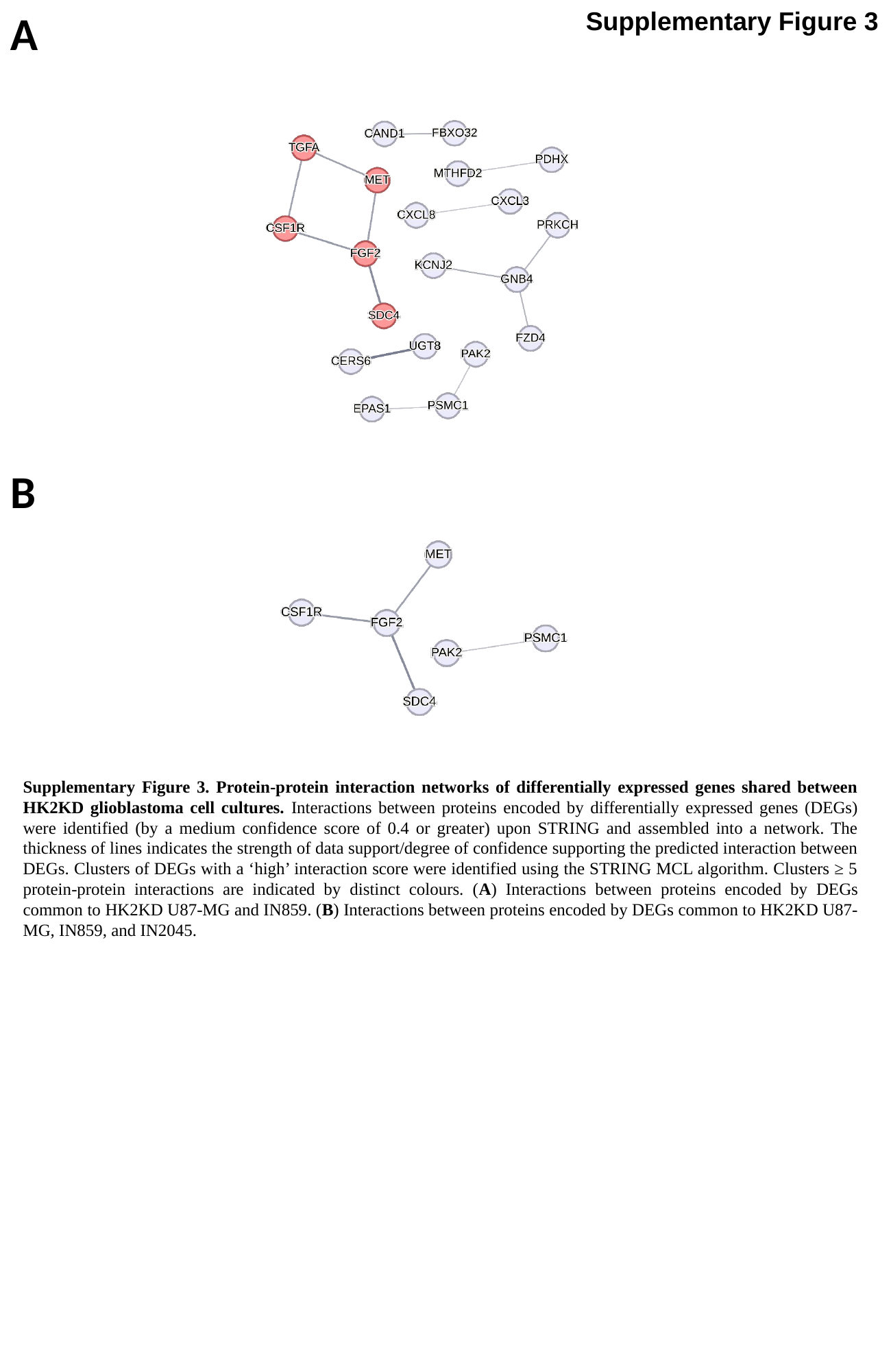

A
Supplementary Figure 3
B
Supplementary Figure 3. Protein-protein interaction networks of differentially expressed genes shared between HK2KD glioblastoma cell cultures. Interactions between proteins encoded by differentially expressed genes (DEGs) were identified (by a medium confidence score of 0.4 or greater) upon STRING and assembled into a network. The thickness of lines indicates the strength of data support/degree of confidence supporting the predicted interaction between DEGs. Clusters of DEGs with a ‘high’ interaction score were identified using the STRING MCL algorithm. Clusters ≥ 5 protein-protein interactions are indicated by distinct colours. (A) Interactions between proteins encoded by DEGs common to HK2KD U87-MG and IN859. (B) Interactions between proteins encoded by DEGs common to HK2KD U87-MG, IN859, and IN2045.

## Slide 4
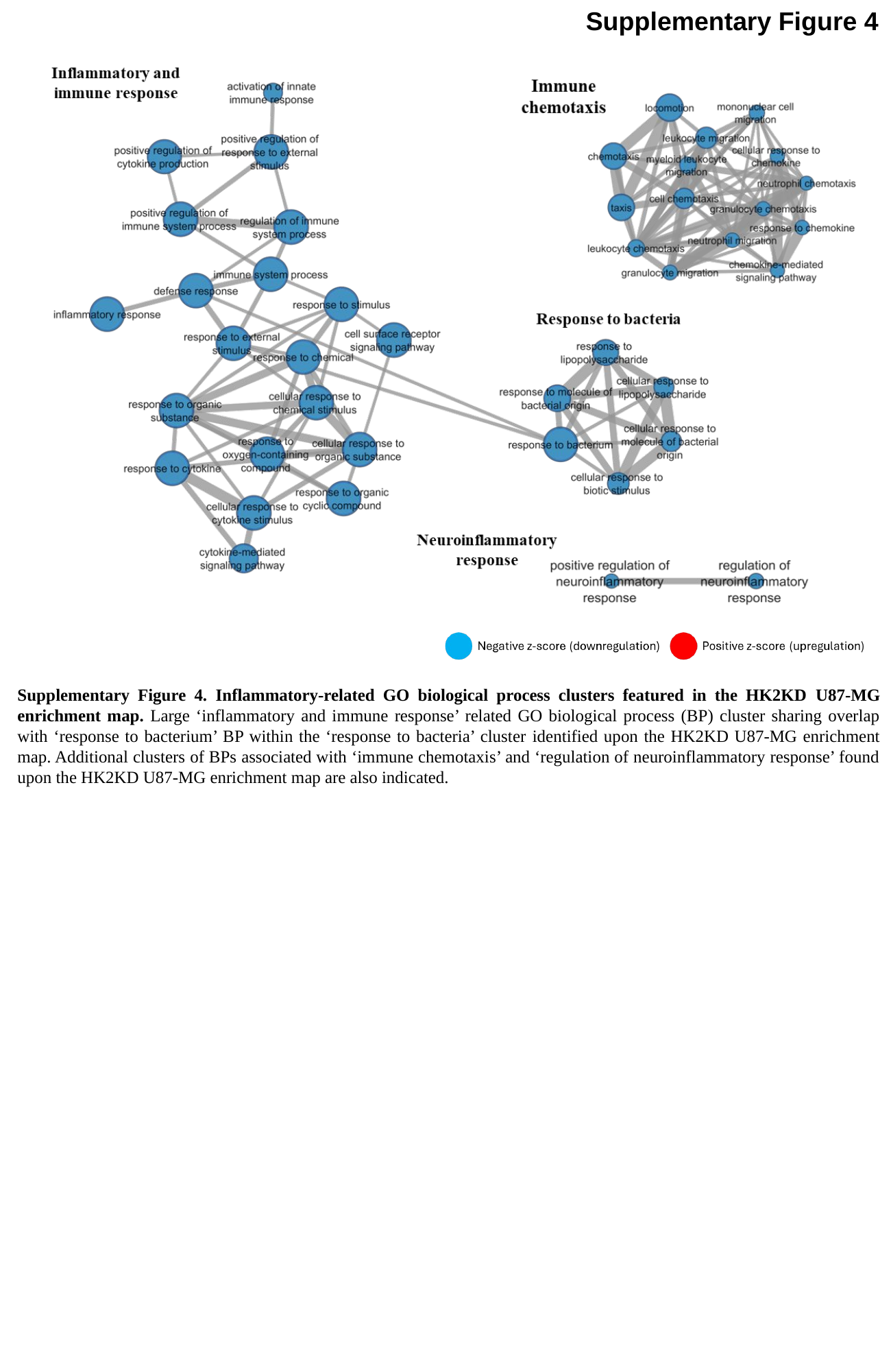

Supplementary Figure 4
Supplementary Figure 4. Inflammatory-related GO biological process clusters featured in the HK2KD U87-MG enrichment map. Large ‘inflammatory and immune response’ related GO biological process (BP) cluster sharing overlap with ‘response to bacterium’ BP within the ‘response to bacteria’ cluster identified upon the HK2KD U87-MG enrichment map. Additional clusters of BPs associated with ‘immune chemotaxis’ and ‘regulation of neuroinflammatory response’ found upon the HK2KD U87-MG enrichment map are also indicated.

## Slide 5
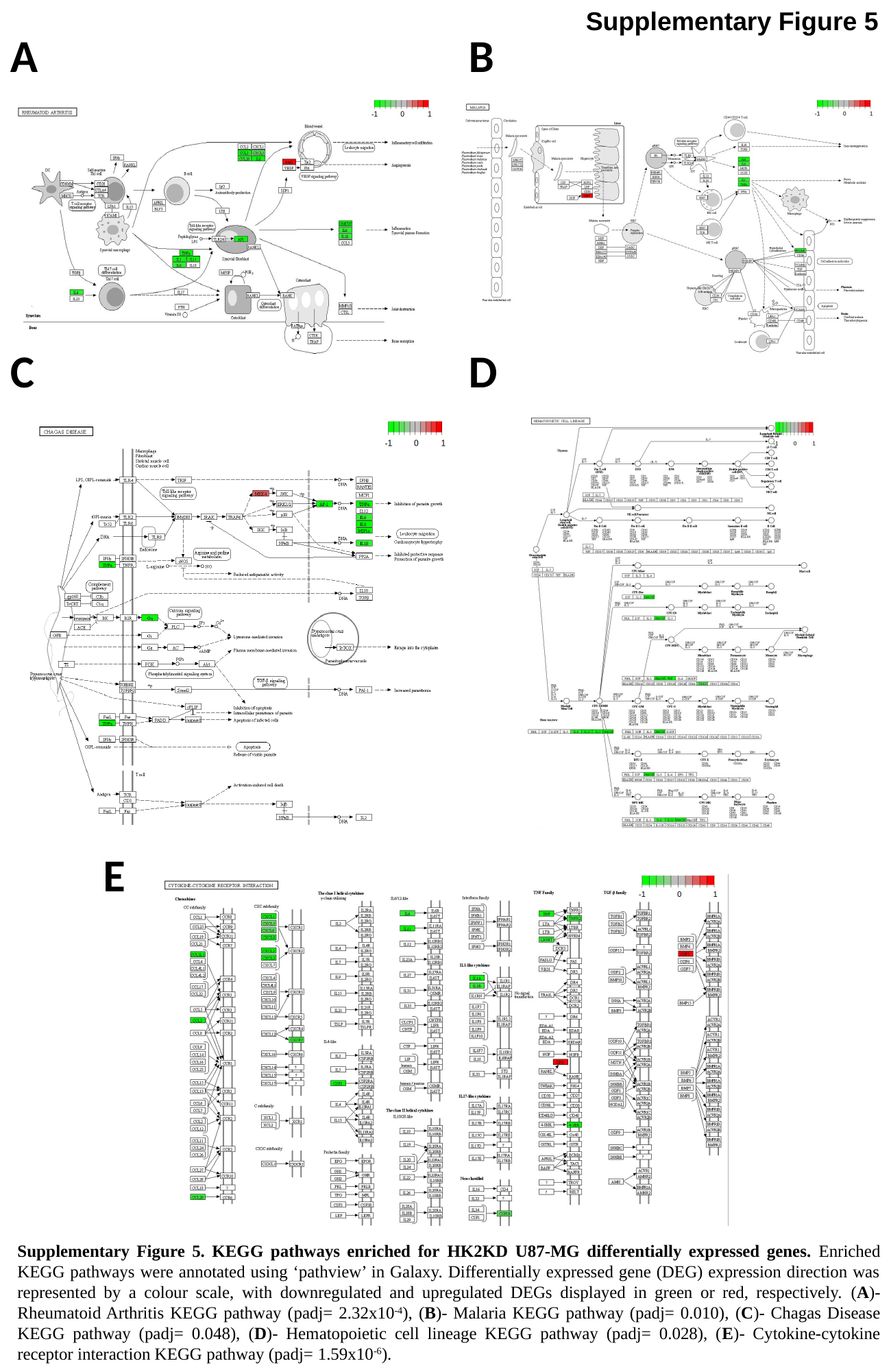

Supplementary Figure 5
A
B
C
D
E
Supplementary Figure 5. KEGG pathways enriched for HK2KD U87-MG differentially expressed genes. Enriched KEGG pathways were annotated using ‘pathview’ in Galaxy. Differentially expressed gene (DEG) expression direction was represented by a colour scale, with downregulated and upregulated DEGs displayed in green or red, respectively. (A)- Rheumatoid Arthritis KEGG pathway (padj= 2.32x10^-4^), (B)- Malaria KEGG pathway (padj= 0.010), (C)- Chagas Disease KEGG pathway (padj= 0.048), (D)- Hematopoietic cell lineage KEGG pathway (padj= 0.028), (E)- Cytokine-cytokine receptor interaction KEGG pathway (padj= 1.59x10^-6^).

## Slide 6
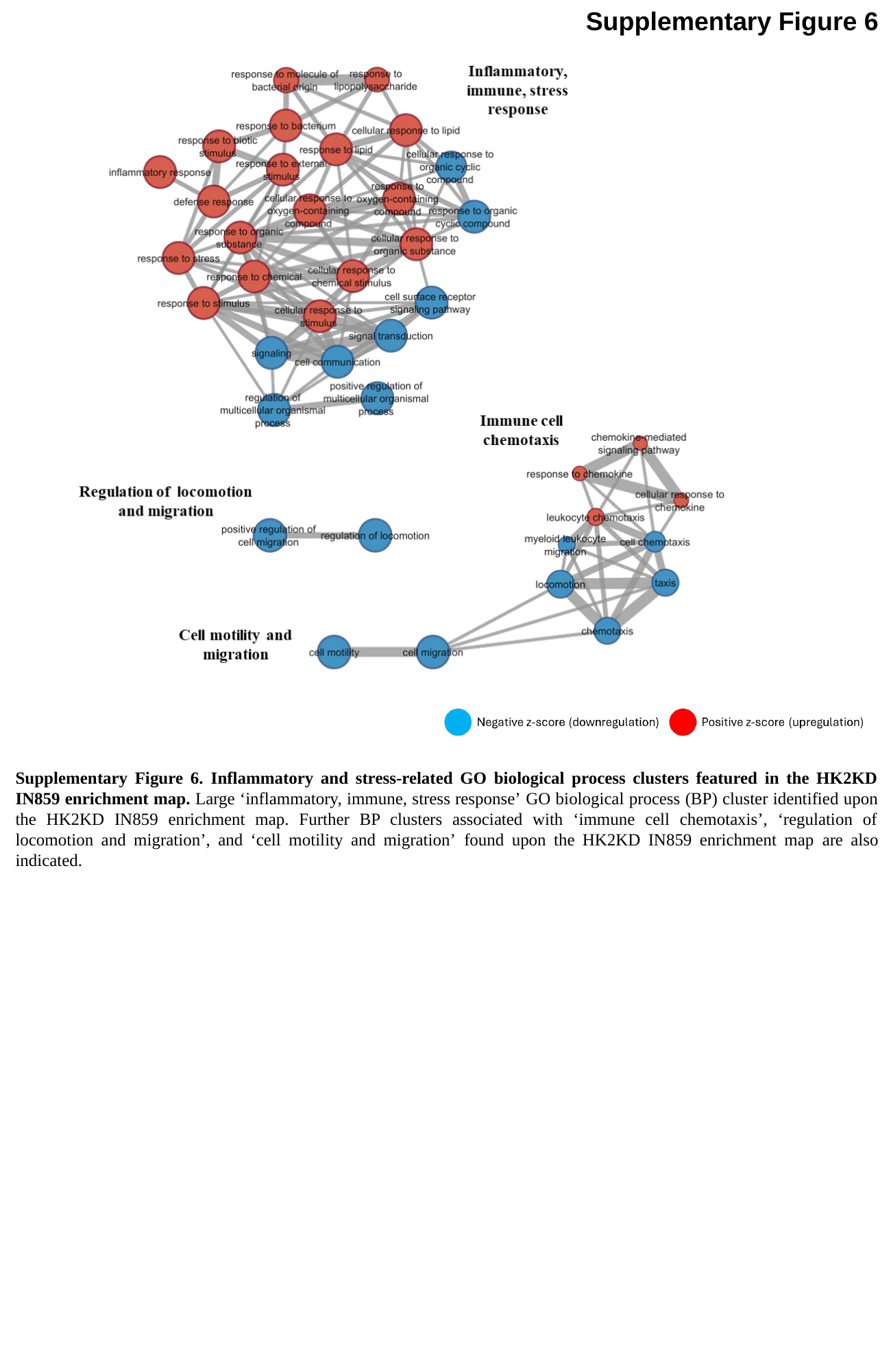

Supplementary Figure 6
Supplementary Figure 6. Inflammatory and stress-related GO biological process clusters featured in the HK2KD IN859 enrichment map. Large ‘inflammatory, immune, stress response’ GO biological process (BP) cluster identified upon the HK2KD IN859 enrichment map. Further BP clusters associated with ‘immune cell chemotaxis’, ‘regulation of locomotion and migration’, and ‘cell motility and migration’ found upon the HK2KD IN859 enrichment map are also indicated.

## Slide 7
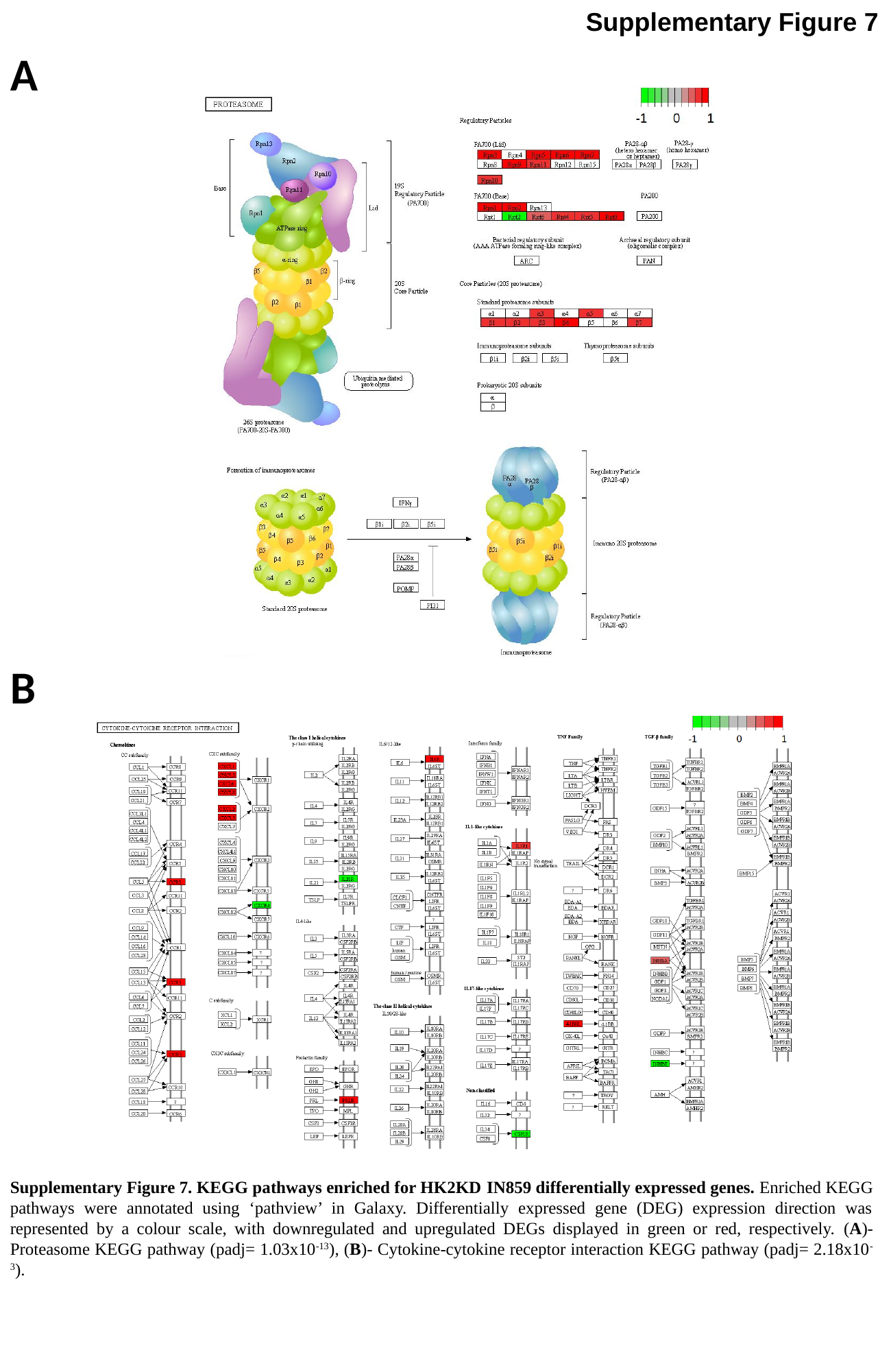

Supplementary Figure 7
A
B
Supplementary Figure 7. KEGG pathways enriched for HK2KD IN859 differentially expressed genes. Enriched KEGG pathways were annotated using ‘pathview’ in Galaxy. Differentially expressed gene (DEG) expression direction was represented by a colour scale, with downregulated and upregulated DEGs displayed in green or red, respectively. (A)- Proteasome KEGG pathway (padj= 1.03x10^-13^), (B)- Cytokine-cytokine receptor interaction KEGG pathway (padj= 2.18x10^-3^).

## Slide 8
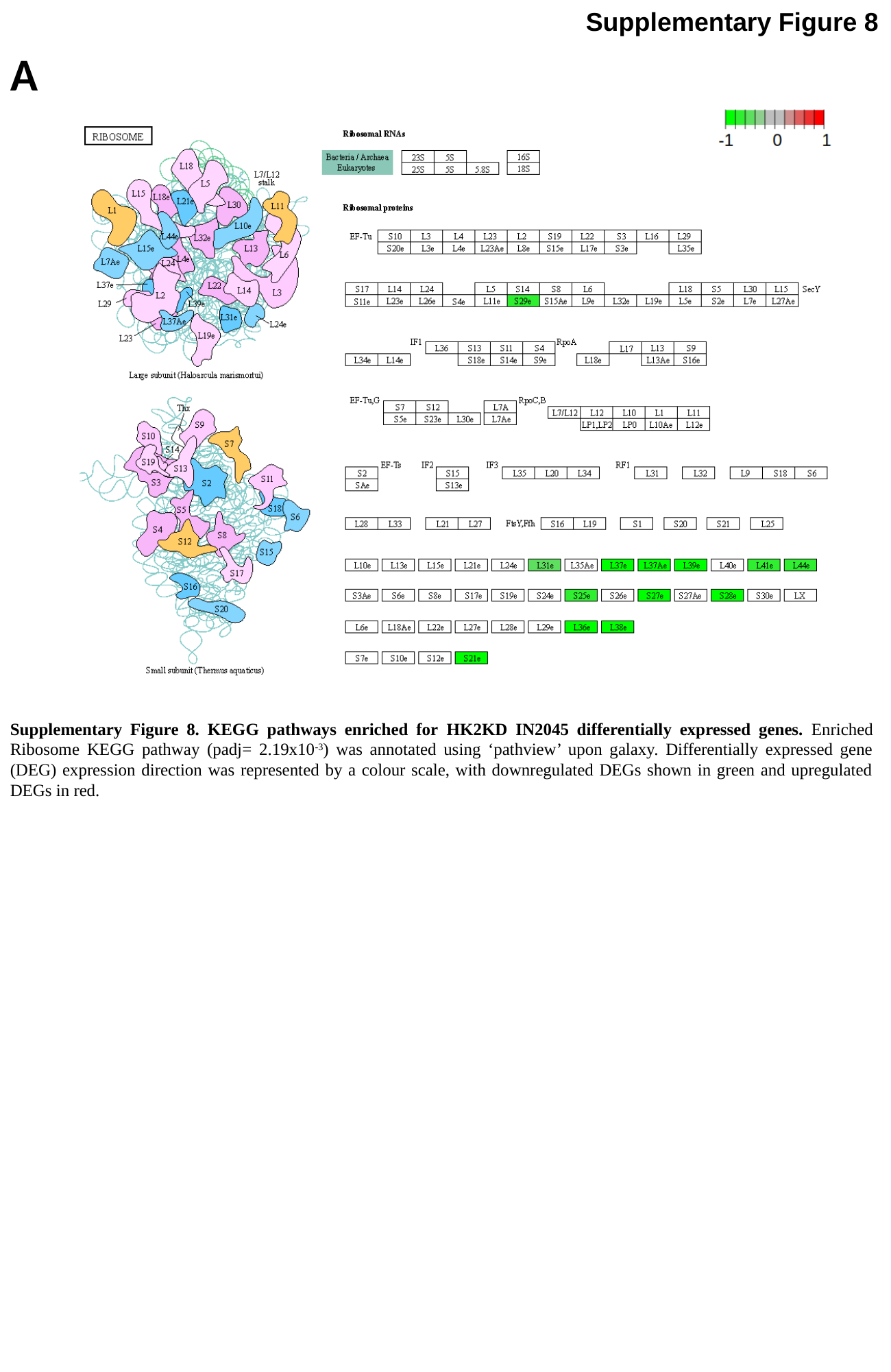

Supplementary Figure 8
A
Supplementary Figure 8. KEGG pathways enriched for HK2KD IN2045 differentially expressed genes. Enriched Ribosome KEGG pathway (padj= 2.19x10^-3^) was annotated using ‘pathview’ upon galaxy. Differentially expressed gene (DEG) expression direction was represented by a colour scale, with downregulated DEGs shown in green and upregulated DEGs in red.

## Slide 9
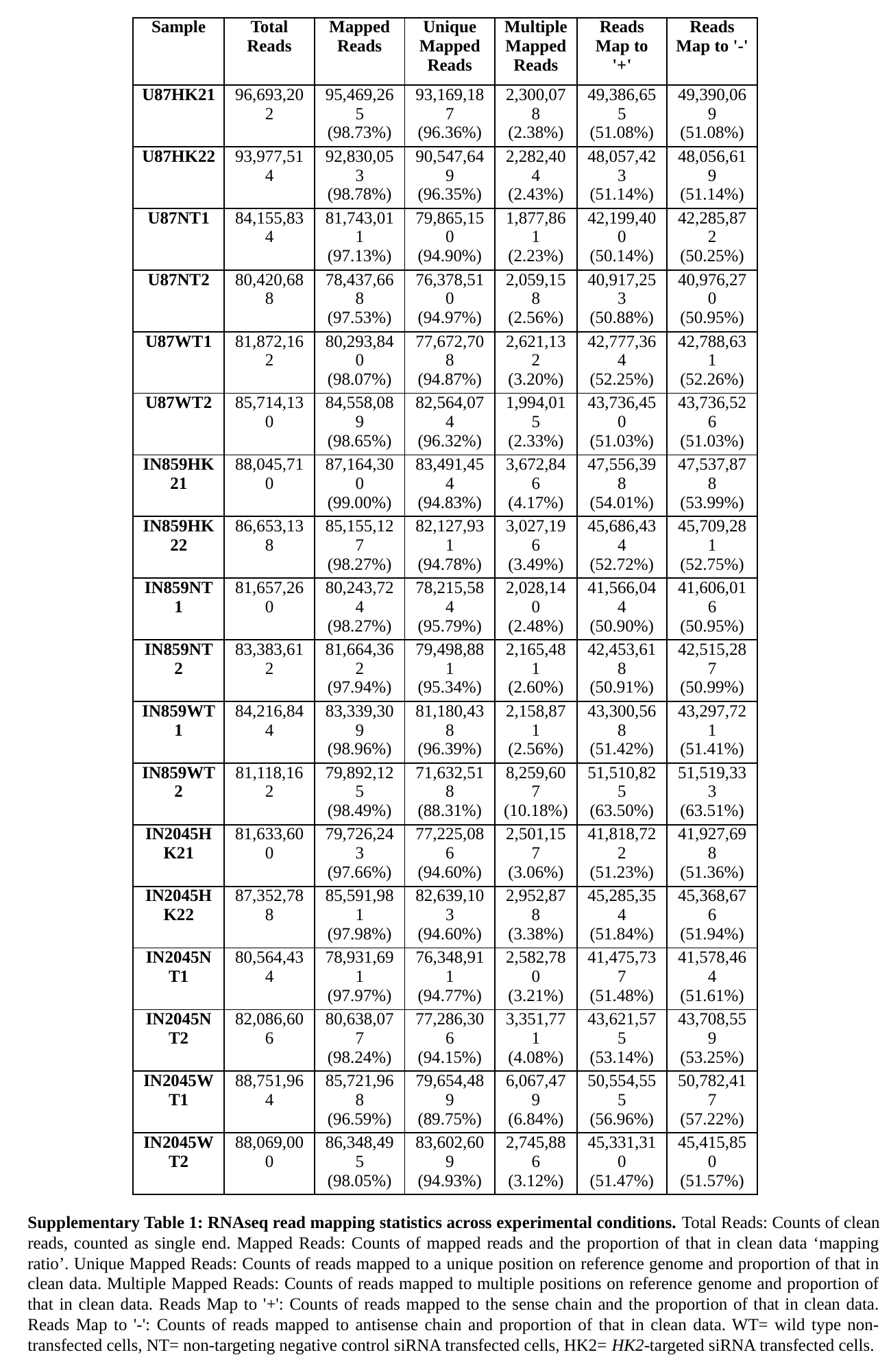

| Sample | Total Reads | Mapped Reads | Unique Mapped Reads | Multiple Mapped Reads | Reads Map to '+' | Reads Map to '-' |
| --- | --- | --- | --- | --- | --- | --- |
| U87HK21 | 96,693,202 | 95,469,265 (98.73%) | 93,169,187 (96.36%) | 2,300,078 (2.38%) | 49,386,655 (51.08%) | 49,390,069 (51.08%) |
| U87HK22 | 93,977,514 | 92,830,053 (98.78%) | 90,547,649 (96.35%) | 2,282,404 (2.43%) | 48,057,423 (51.14%) | 48,056,619 (51.14%) |
| U87NT1 | 84,155,834 | 81,743,011 (97.13%) | 79,865,150 (94.90%) | 1,877,861 (2.23%) | 42,199,400 (50.14%) | 42,285,872 (50.25%) |
| U87NT2 | 80,420,688 | 78,437,668 (97.53%) | 76,378,510 (94.97%) | 2,059,158 (2.56%) | 40,917,253 (50.88%) | 40,976,270 (50.95%) |
| U87WT1 | 81,872,162 | 80,293,840 (98.07%) | 77,672,708 (94.87%) | 2,621,132 (3.20%) | 42,777,364 (52.25%) | 42,788,631 (52.26%) |
| U87WT2 | 85,714,130 | 84,558,089 (98.65%) | 82,564,074 (96.32%) | 1,994,015 (2.33%) | 43,736,450 (51.03%) | 43,736,526 (51.03%) |
| IN859HK21 | 88,045,710 | 87,164,300 (99.00%) | 83,491,454 (94.83%) | 3,672,846 (4.17%) | 47,556,398 (54.01%) | 47,537,878 (53.99%) |
| IN859HK22 | 86,653,138 | 85,155,127 (98.27%) | 82,127,931 (94.78%) | 3,027,196 (3.49%) | 45,686,434 (52.72%) | 45,709,281 (52.75%) |
| IN859NT1 | 81,657,260 | 80,243,724 (98.27%) | 78,215,584 (95.79%) | 2,028,140 (2.48%) | 41,566,044 (50.90%) | 41,606,016 (50.95%) |
| IN859NT2 | 83,383,612 | 81,664,362 (97.94%) | 79,498,881 (95.34%) | 2,165,481 (2.60%) | 42,453,618 (50.91%) | 42,515,287 (50.99%) |
| IN859WT1 | 84,216,844 | 83,339,309 (98.96%) | 81,180,438 (96.39%) | 2,158,871 (2.56%) | 43,300,568 (51.42%) | 43,297,721 (51.41%) |
| IN859WT2 | 81,118,162 | 79,892,125 (98.49%) | 71,632,518 (88.31%) | 8,259,607 (10.18%) | 51,510,825 (63.50%) | 51,519,333 (63.51%) |
| IN2045HK21 | 81,633,600 | 79,726,243 (97.66%) | 77,225,086 (94.60%) | 2,501,157 (3.06%) | 41,818,722 (51.23%) | 41,927,698 (51.36%) |
| IN2045HK22 | 87,352,788 | 85,591,981 (97.98%) | 82,639,103 (94.60%) | 2,952,878 (3.38%) | 45,285,354 (51.84%) | 45,368,676 (51.94%) |
| IN2045NT1 | 80,564,434 | 78,931,691 (97.97%) | 76,348,911 (94.77%) | 2,582,780 (3.21%) | 41,475,737 (51.48%) | 41,578,464 (51.61%) |
| IN2045NT2 | 82,086,606 | 80,638,077 (98.24%) | 77,286,306 (94.15%) | 3,351,771 (4.08%) | 43,621,575 (53.14%) | 43,708,559 (53.25%) |
| IN2045WT1 | 88,751,964 | 85,721,968 (96.59%) | 79,654,489 (89.75%) | 6,067,479 (6.84%) | 50,554,555 (56.96%) | 50,782,417 (57.22%) |
| IN2045WT2 | 88,069,000 | 86,348,495 (98.05%) | 83,602,609 (94.93%) | 2,745,886 (3.12%) | 45,331,310 (51.47%) | 45,415,850 (51.57%) |
Supplementary Table 1: RNAseq read mapping statistics across experimental conditions. Total Reads: Counts of clean reads, counted as single end. Mapped Reads: Counts of mapped reads and the proportion of that in clean data ‘mapping ratio’. Unique Mapped Reads: Counts of reads mapped to a unique position on reference genome and proportion of that in clean data. Multiple Mapped Reads: Counts of reads mapped to multiple positions on reference genome and proportion of that in clean data. Reads Map to '+': Counts of reads mapped to the sense chain and the proportion of that in clean data. Reads Map to '-': Counts of reads mapped to antisense chain and proportion of that in clean data. WT= wild type non-transfected cells, NT= non-targeting negative control siRNA transfected cells, HK2= HK2-targeted siRNA transfected cells.

## Slide 10
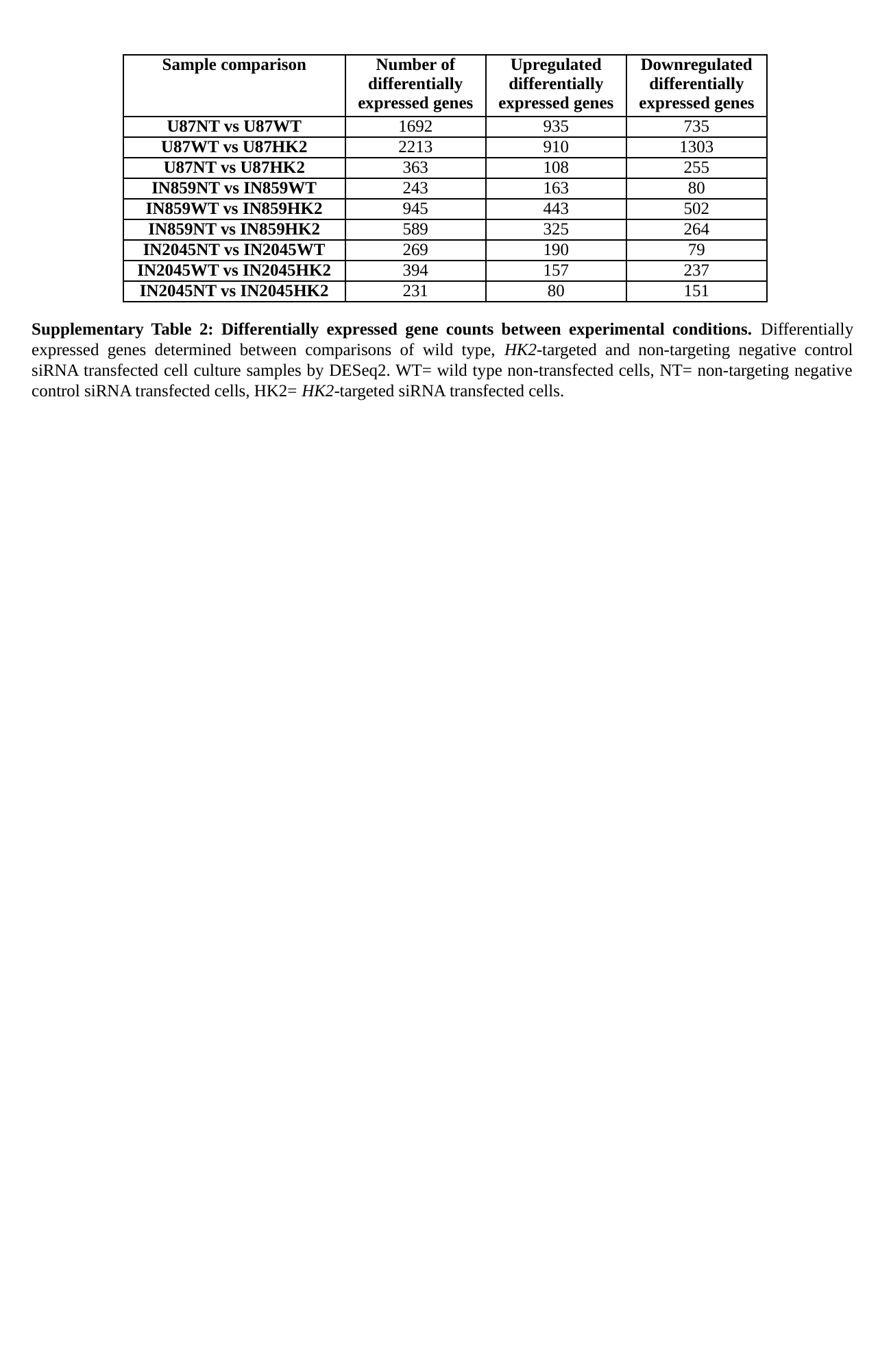

| Sample comparison | Number of differentially expressed genes | Upregulated differentially expressed genes | Downregulated differentially expressed genes |
| --- | --- | --- | --- |
| U87NT vs U87WT | 1692 | 935 | 735 |
| U87WT vs U87HK2 | 2213 | 910 | 1303 |
| U87NT vs U87HK2 | 363 | 108 | 255 |
| IN859NT vs IN859WT | 243 | 163 | 80 |
| IN859WT vs IN859HK2 | 945 | 443 | 502 |
| IN859NT vs IN859HK2 | 589 | 325 | 264 |
| IN2045NT vs IN2045WT | 269 | 190 | 79 |
| IN2045WT vs IN2045HK2 | 394 | 157 | 237 |
| IN2045NT vs IN2045HK2 | 231 | 80 | 151 |
Supplementary Table 2: Differentially expressed gene counts between experimental conditions. Differentially expressed genes determined between comparisons of wild type, HK2-targeted and non-targeting negative control siRNA transfected cell culture samples by DESeq2. WT= wild type non-transfected cells, NT= non-targeting negative control siRNA transfected cells, HK2= HK2-targeted siRNA transfected cells.

## Slide 11
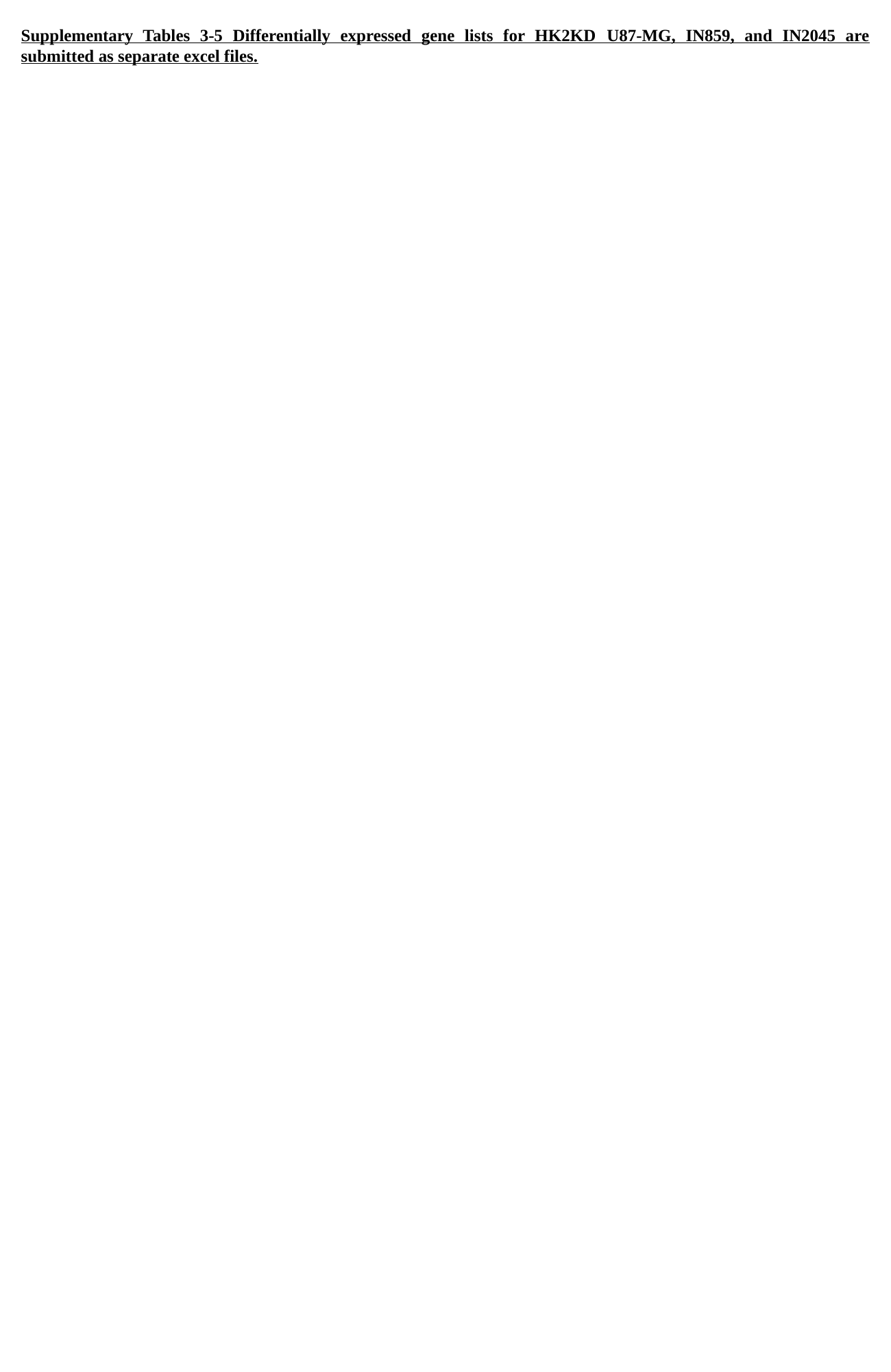

Supplementary Tables 3-5 Differentially expressed gene lists for HK2KD U87-MG, IN859, and IN2045 are submitted as separate excel files.

## Slide 12
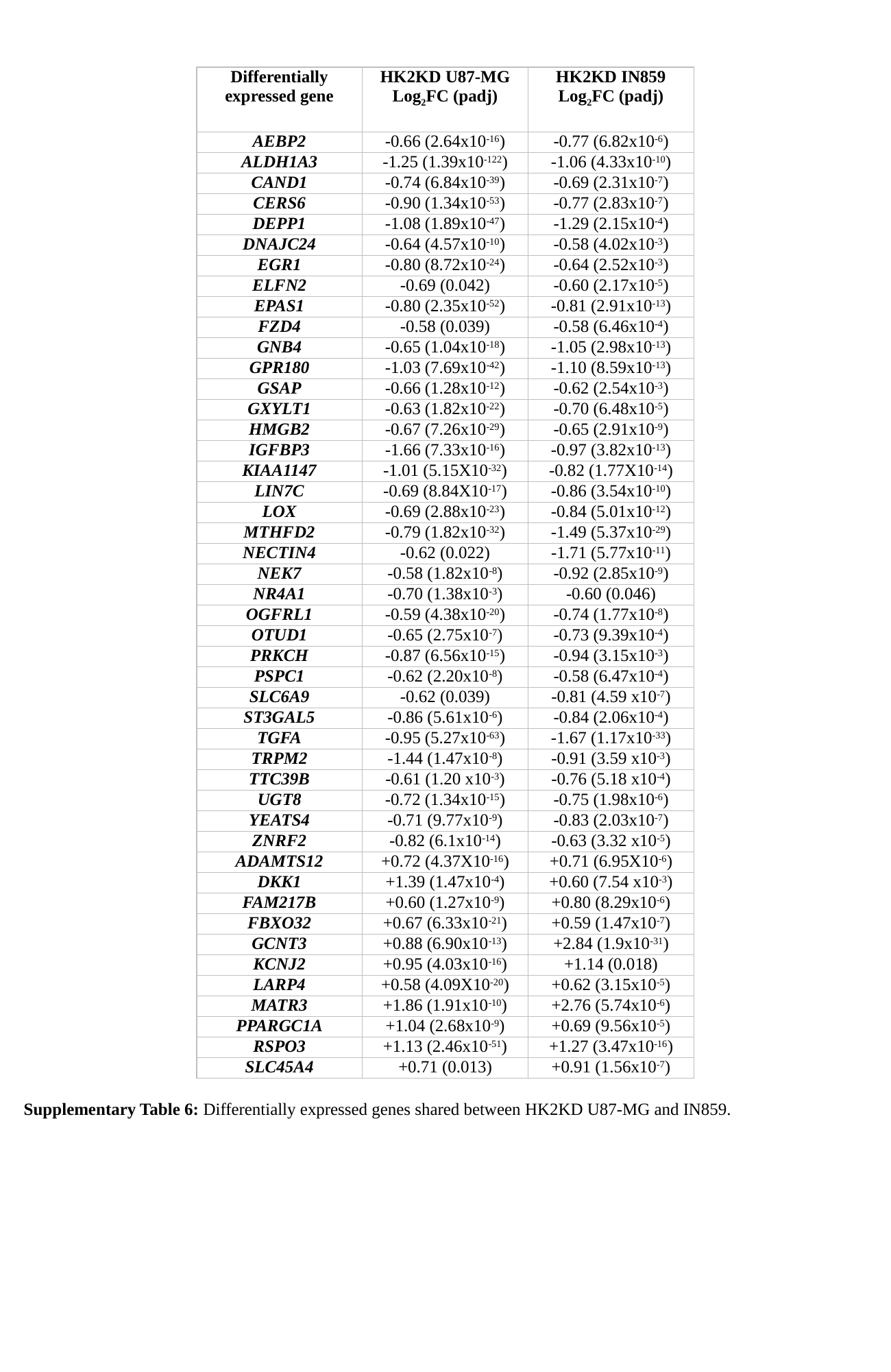

| Differentially expressed gene | HK2KD U87-MG Log2FC (padj) | HK2KD IN859 Log2FC (padj) |
| --- | --- | --- |
| AEBP2 | -0.66 (2.64x10^-16^) | -0.77 (6.82x10^-6^) |
| ALDH1A3 | -1.25 (1.39x10-122) | -1.06 (4.33x10^-10^) |
| CAND1 | -0.74 (6.84x10^-39^) | -0.69 (2.31x10^-7^) |
| CERS6 | -0.90 (1.34x10^-53^) | -0.77 (2.83x10^-7^) |
| DEPP1 | -1.08 (1.89x10^-47^) | -1.29 (2.15x10^-4^) |
| DNAJC24 | -0.64 (4.57x10^-10^) | -0.58 (4.02x10^-3^) |
| EGR1 | -0.80 (8.72x10^-24^) | -0.64 (2.52x10^-3^) |
| ELFN2 | -0.69 (0.042) | -0.60 (2.17x10^-5^) |
| EPAS1 | -0.80 (2.35x10^-52^) | -0.81 (2.91x10^-13^) |
| FZD4 | -0.58 (0.039) | -0.58 (6.46x10^-4^) |
| GNB4 | -0.65 (1.04x10^-18^) | -1.05 (2.98x10^-13^) |
| GPR180 | -1.03 (7.69x10^-42^) | -1.10 (8.59x10^-13^) |
| GSAP | -0.66 (1.28x10^-12^) | -0.62 (2.54x10^-3^) |
| GXYLT1 | -0.63 (1.82x10^-22^) | -0.70 (6.48x10^-5^) |
| HMGB2 | -0.67 (7.26x10^-29^) | -0.65 (2.91x10^-9^) |
| IGFBP3 | -1.66 (7.33x10^-16^) | -0.97 (3.82x10^-13^) |
| KIAA1147 | -1.01 (5.15X10-32) | -0.82 (1.77X10-14) |
| LIN7C | -0.69 (8.84X10-17) | -0.86 (3.54x10^-10^) |
| LOX | -0.69 (2.88x10^-23^) | -0.84 (5.01x10^-12^) |
| MTHFD2 | -0.79 (1.82x10^-32^) | -1.49 (5.37x10^-29^) |
| NECTIN4 | -0.62 (0.022) | -1.71 (5.77x10^-11^) |
| NEK7 | -0.58 (1.82x10^-8^) | -0.92 (2.85x10^-9^) |
| NR4A1 | -0.70 (1.38x10^-3^) | -0.60 (0.046) |
| OGFRL1 | -0.59 (4.38x10^-20^) | -0.74 (1.77x10^-8^) |
| OTUD1 | -0.65 (2.75x10^-7^) | -0.73 (9.39x10^-4^) |
| PRKCH | -0.87 (6.56x10^-15^) | -0.94 (3.15x10^-3^) |
| PSPC1 | -0.62 (2.20x10^-8^) | -0.58 (6.47x10^-4^) |
| SLC6A9 | -0.62 (0.039) | -0.81 (4.59 x10^-7^) |
| ST3GAL5 | -0.86 (5.61x10^-6^) | -0.84 (2.06x10^-4^) |
| TGFA | -0.95 (5.27x10^-63^) | -1.67 (1.17x10^-33^) |
| TRPM2 | -1.44 (1.47x10^-8^) | -0.91 (3.59 x10^-3^) |
| TTC39B | -0.61 (1.20 x10^-3^) | -0.76 (5.18 x10^-4^) |
| UGT8 | -0.72 (1.34x10^-15^) | -0.75 (1.98x10^-6^) |
| YEATS4 | -0.71 (9.77x10^-9^) | -0.83 (2.03x10^-7^) |
| ZNRF2 | -0.82 (6.1x10^-14^) | -0.63 (3.32 x10^-5^) |
| ADAMTS12 | +0.72 (4.37X10-16) | +0.71 (6.95X10-6) |
| DKK1 | +1.39 (1.47x10^-4^) | +0.60 (7.54 x10^-3^) |
| FAM217B | +0.60 (1.27x10^-9^) | +0.80 (8.29x10^-6^) |
| FBXO32 | +0.67 (6.33x10^-21^) | +0.59 (1.47x10^-7^) |
| GCNT3 | +0.88 (6.90x10^-13^) | +2.84 (1.9x10^-31^) |
| KCNJ2 | +0.95 (4.03x10^-16^) | +1.14 (0.018) |
| LARP4 | +0.58 (4.09X10-20) | +0.62 (3.15x10^-5^) |
| MATR3 | +1.86 (1.91x10^-10^) | +2.76 (5.74x10^-6^) |
| PPARGC1A | +1.04 (2.68x10^-9^) | +0.69 (9.56x10^-5^) |
| RSPO3 | +1.13 (2.46x10^-51^) | +1.27 (3.47x10^-16^) |
| SLC45A4 | +0.71 (0.013) | +0.91 (1.56x10^-7^) |
Supplementary Table 6: Differentially expressed genes shared between HK2KD U87-MG and IN859.

## Slide 13
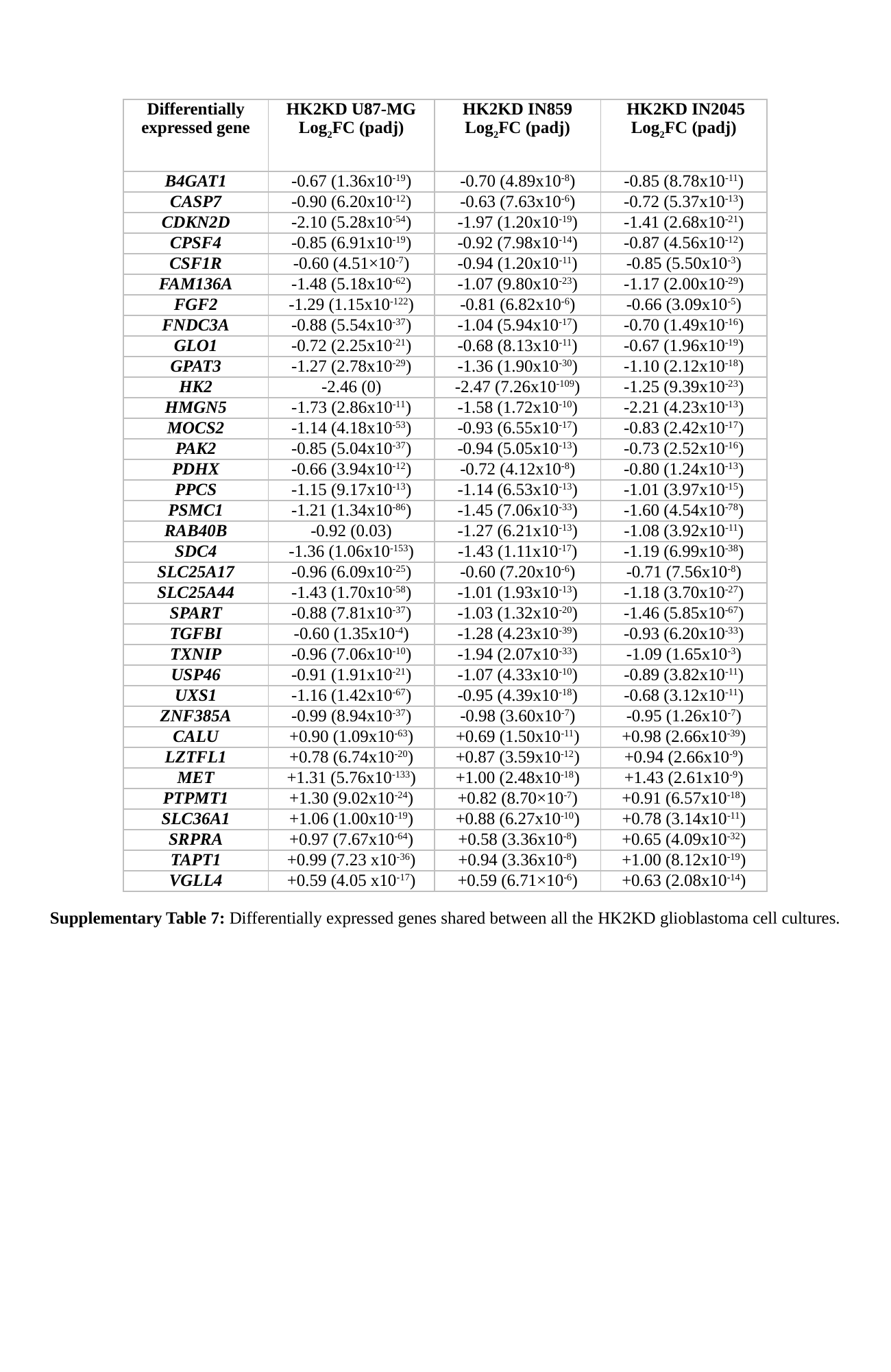

| Differentially expressed gene | HK2KD U87-MG Log2FC (padj) | HK2KD IN859 Log2FC (padj) | HK2KD IN2045 Log2FC (padj) |
| --- | --- | --- | --- |
| B4GAT1 | -0.67 (1.36x10^-19^) | -0.70 (4.89x10^-8^) | -0.85 (8.78x10^-11^) |
| CASP7 | -0.90 (6.20x10^-12^) | -0.63 (7.63x10^-6^) | -0.72 (5.37x10^-13^) |
| CDKN2D | -2.10 (5.28x10^-54^) | -1.97 (1.20x10^-19^) | -1.41 (2.68x10^-21^) |
| CPSF4 | -0.85 (6.91x10^-19^) | -0.92 (7.98x10^-14^) | -0.87 (4.56x10^-12^) |
| CSF1R | -0.60 (4.51×10^-7^) | -0.94 (1.20x10^-11^) | -0.85 (5.50x10^-3^) |
| FAM136A | -1.48 (5.18x10^-62^) | -1.07 (9.80x10^-23^) | -1.17 (2.00x10^-29^) |
| FGF2 | -1.29 (1.15x10-122) | -0.81 (6.82x10^-6^) | -0.66 (3.09x10^-5^) |
| FNDC3A | -0.88 (5.54x10^-37^) | -1.04 (5.94x10^-17^) | -0.70 (1.49x10^-16^) |
| GLO1 | -0.72 (2.25x10^-21^) | -0.68 (8.13x10^-11^) | -0.67 (1.96x10^-19^) |
| GPAT3 | -1.27 (2.78x10^-29^) | -1.36 (1.90x10^-30^) | -1.10 (2.12x10^-18^) |
| HK2 | -2.46 (0) | -2.47 (7.26x10-109) | -1.25 (9.39x10^-23^) |
| HMGN5 | -1.73 (2.86x10^-11^) | -1.58 (1.72x10^-10^) | -2.21 (4.23x10^-13^) |
| MOCS2 | -1.14 (4.18x10^-53^) | -0.93 (6.55x10^-17^) | -0.83 (2.42x10^-17^) |
| PAK2 | -0.85 (5.04x10^-37^) | -0.94 (5.05x10^-13^) | -0.73 (2.52x10^-16^) |
| PDHX | -0.66 (3.94x10^-12^) | -0.72 (4.12x10^-8^) | -0.80 (1.24x10^-13^) |
| PPCS | -1.15 (9.17x10^-13^) | -1.14 (6.53x10^-13^) | -1.01 (3.97x10^-15^) |
| PSMC1 | -1.21 (1.34x10^-86^) | -1.45 (7.06x10^-33^) | -1.60 (4.54x10^-78^) |
| RAB40B | -0.92 (0.03) | -1.27 (6.21x10^-13^) | -1.08 (3.92x10^-11^) |
| SDC4 | -1.36 (1.06x10-153) | -1.43 (1.11x10^-17^) | -1.19 (6.99x10^-38^) |
| SLC25A17 | -0.96 (6.09x10^-25^) | -0.60 (7.20x10^-6^) | -0.71 (7.56x10^-8^) |
| SLC25A44 | -1.43 (1.70x10^-58^) | -1.01 (1.93x10^-13^) | -1.18 (3.70x10^-27^) |
| SPART | -0.88 (7.81x10^-37^) | -1.03 (1.32x10^-20^) | -1.46 (5.85x10^-67^) |
| TGFBI | -0.60 (1.35x10^-4^) | -1.28 (4.23x10^-39^) | -0.93 (6.20x10^-33^) |
| TXNIP | -0.96 (7.06x10^-10^) | -1.94 (2.07x10^-33^) | -1.09 (1.65x10^-3^) |
| USP46 | -0.91 (1.91x10^-21^) | -1.07 (4.33x10^-10^) | -0.89 (3.82x10^-11^) |
| UXS1 | -1.16 (1.42x10^-67^) | -0.95 (4.39x10^-18^) | -0.68 (3.12x10^-11^) |
| ZNF385A | -0.99 (8.94x10^-37^) | -0.98 (3.60x10^-7^) | -0.95 (1.26x10^-7^) |
| CALU | +0.90 (1.09x10^-63^) | +0.69 (1.50x10^-11^) | +0.98 (2.66x10^-39^) |
| LZTFL1 | +0.78 (6.74x10^-20^) | +0.87 (3.59x10^-12^) | +0.94 (2.66x10^-9^) |
| MET | +1.31 (5.76x10-133) | +1.00 (2.48x10^-18^) | +1.43 (2.61x10^-9^) |
| PTPMT1 | +1.30 (9.02x10^-24^) | +0.82 (8.70×10^-7^) | +0.91 (6.57x10^-18^) |
| SLC36A1 | +1.06 (1.00x10^-19^) | +0.88 (6.27x10^-10^) | +0.78 (3.14x10^-11^) |
| SRPRA | +0.97 (7.67x10^-64^) | +0.58 (3.36x10^-8^) | +0.65 (4.09x10^-32^) |
| TAPT1 | +0.99 (7.23 x10^-36^) | +0.94 (3.36x10^-8^) | +1.00 (8.12x10^-19^) |
| VGLL4 | +0.59 (4.05 x10^-17^) | +0.59 (6.71×10^-6^) | +0.63 (2.08x10^-14^) |
Supplementary Table 7: Differentially expressed genes shared between all the HK2KD glioblastoma cell cultures.

## Slide 14
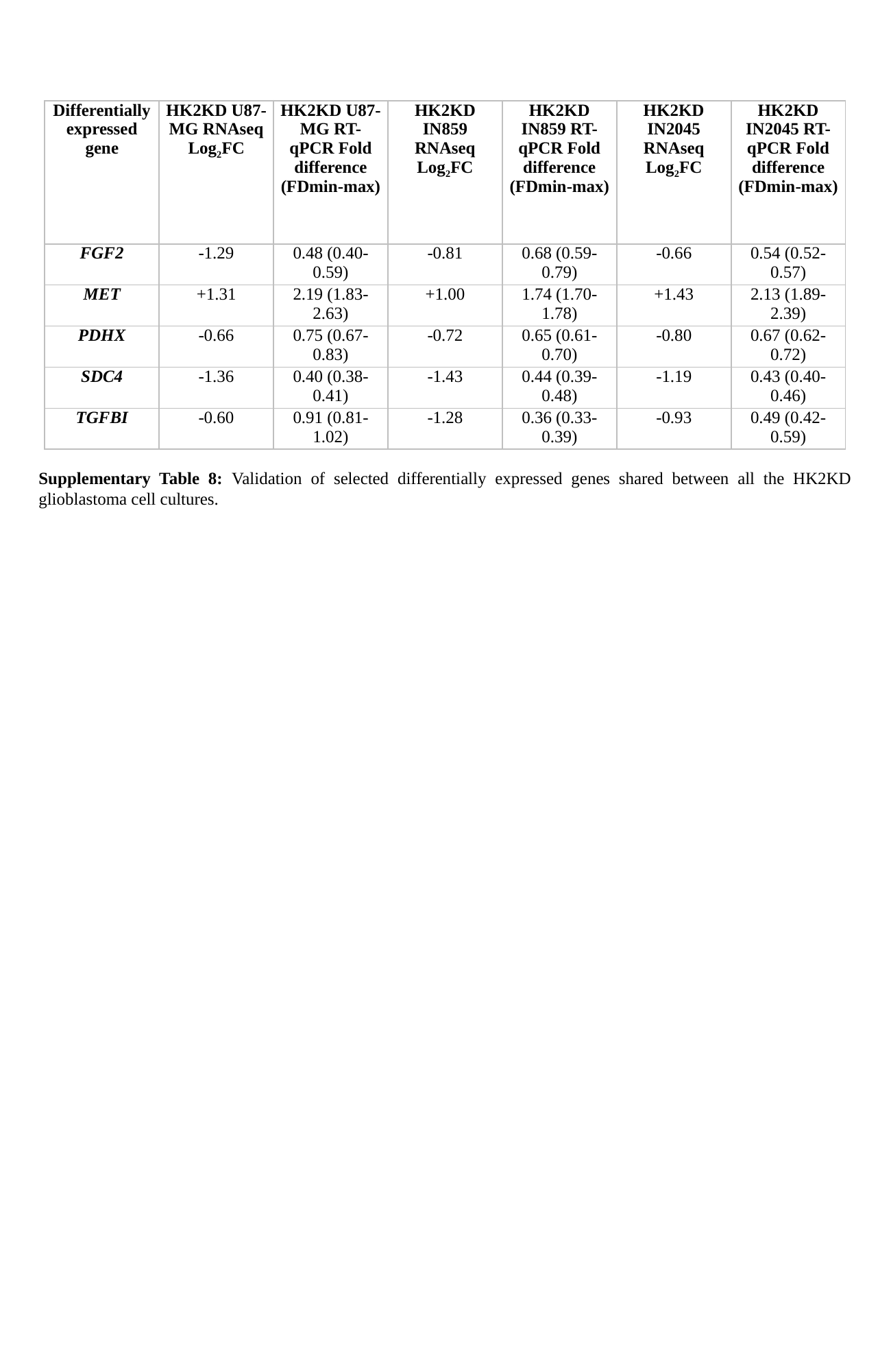

| Differentially expressed gene | HK2KD U87-MG RNAseq Log2FC | HK2KD U87-MG RT-qPCR Fold difference (FDmin-max) | HK2KD IN859 RNAseq Log2FC | HK2KD IN859 RT-qPCR Fold difference (FDmin-max) | HK2KD IN2045 RNAseq Log2FC | HK2KD IN2045 RT-qPCR Fold difference (FDmin-max) |
| --- | --- | --- | --- | --- | --- | --- |
| FGF2 | -1.29 | 0.48 (0.40-0.59) | -0.81 | 0.68 (0.59-0.79) | -0.66 | 0.54 (0.52-0.57) |
| MET | +1.31 | 2.19 (1.83-2.63) | +1.00 | 1.74 (1.70-1.78) | +1.43 | 2.13 (1.89-2.39) |
| PDHX | -0.66 | 0.75 (0.67-0.83) | -0.72 | 0.65 (0.61-0.70) | -0.80 | 0.67 (0.62-0.72) |
| SDC4 | -1.36 | 0.40 (0.38-0.41) | -1.43 | 0.44 (0.39-0.48) | -1.19 | 0.43 (0.40-0.46) |
| TGFBI | -0.60 | 0.91 (0.81-1.02) | -1.28 | 0.36 (0.33-0.39) | -0.93 | 0.49 (0.42-0.59) |
Supplementary Table 8: Validation of selected differentially expressed genes shared between all the HK2KD glioblastoma cell cultures.

## Slide 15
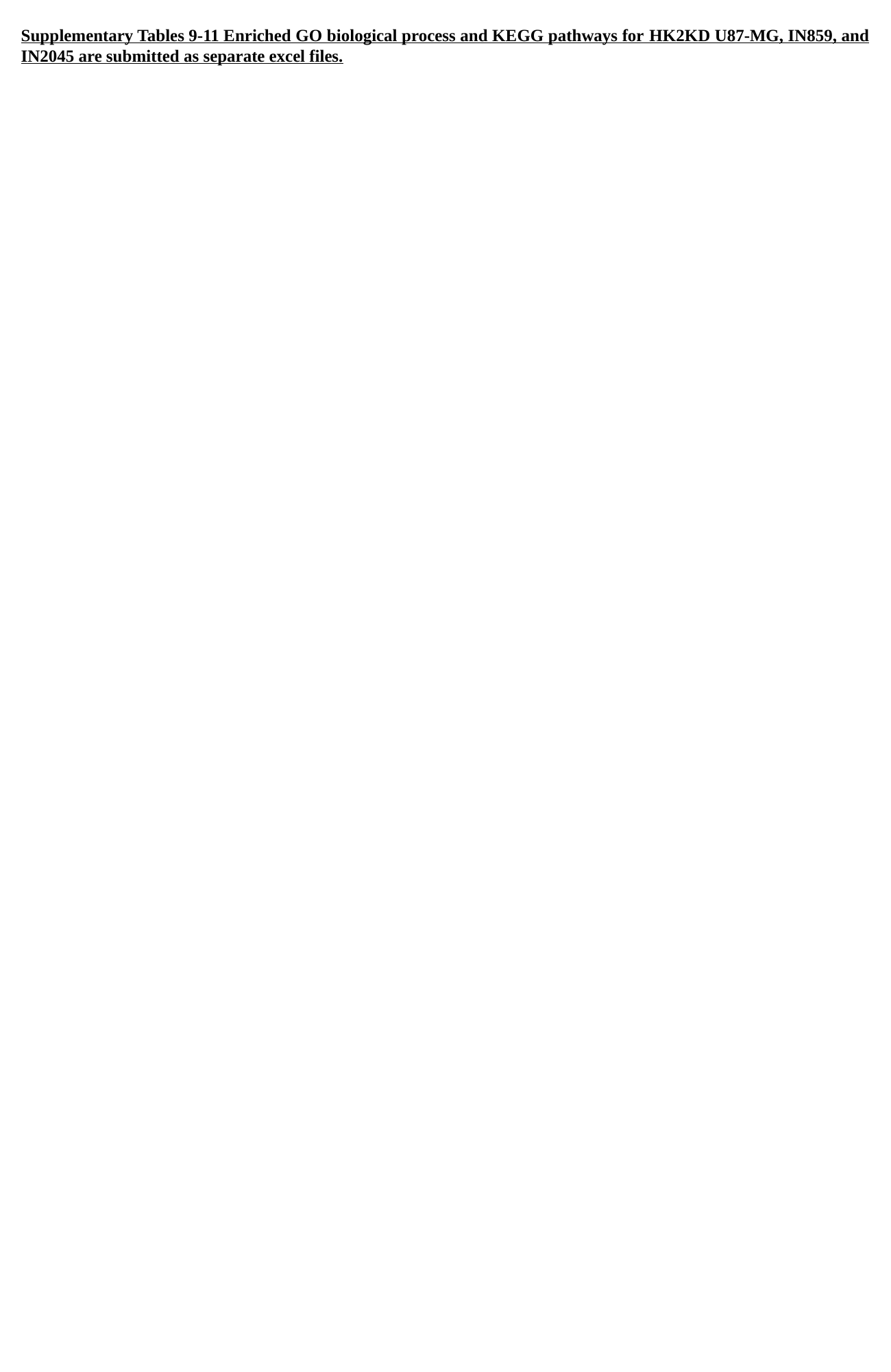

Supplementary Tables 9-11 Enriched GO biological process and KEGG pathways for HK2KD U87-MG, IN859, and IN2045 are submitted as separate excel files.

## Slide 16
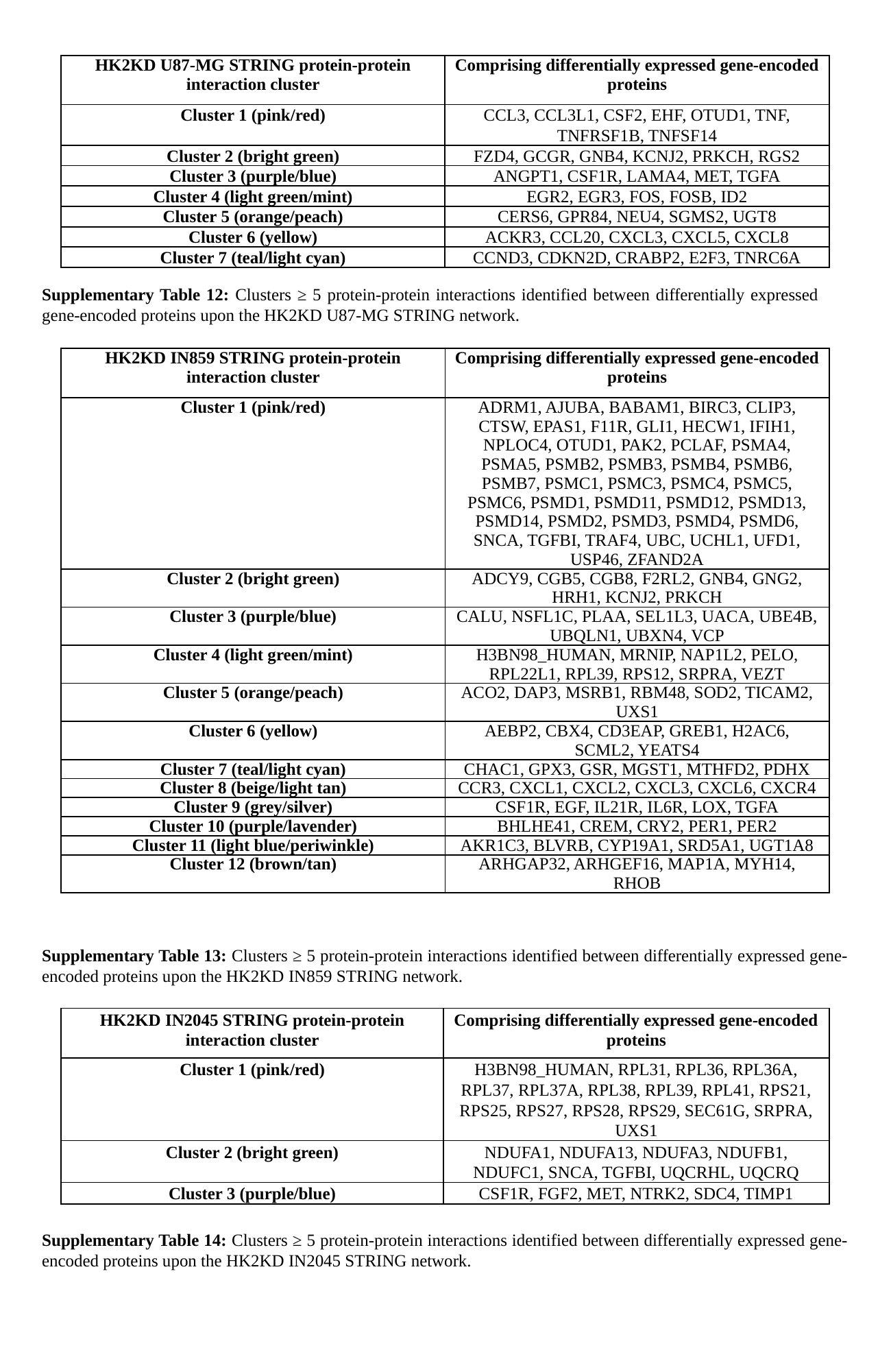

| HK2KD U87-MG STRING protein-protein interaction cluster | Comprising differentially expressed gene-encoded proteins |
| --- | --- |
| Cluster 1 (pink/red) | CCL3, CCL3L1, CSF2, EHF, OTUD1, TNF, TNFRSF1B, TNFSF14 |
| Cluster 2 (bright green) | FZD4, GCGR, GNB4, KCNJ2, PRKCH, RGS2 |
| Cluster 3 (purple/blue) | ANGPT1, CSF1R, LAMA4, MET, TGFA |
| Cluster 4 (light green/mint) | EGR2, EGR3, FOS, FOSB, ID2 |
| Cluster 5 (orange/peach) | CERS6, GPR84, NEU4, SGMS2, UGT8 |
| Cluster 6 (yellow) | ACKR3, CCL20, CXCL3, CXCL5, CXCL8 |
| Cluster 7 (teal/light cyan) | CCND3, CDKN2D, CRABP2, E2F3, TNRC6A |
Supplementary Table 12: Clusters ≥ 5 protein-protein interactions identified between differentially expressed gene-encoded proteins upon the HK2KD U87-MG STRING network.
| HK2KD IN859 STRING protein-protein interaction cluster | Comprising differentially expressed gene-encoded proteins |
| --- | --- |
| Cluster 1 (pink/red) | ADRM1, AJUBA, BABAM1, BIRC3, CLIP3, CTSW, EPAS1, F11R, GLI1, HECW1, IFIH1, NPLOC4, OTUD1, PAK2, PCLAF, PSMA4, PSMA5, PSMB2, PSMB3, PSMB4, PSMB6, PSMB7, PSMC1, PSMC3, PSMC4, PSMC5, PSMC6, PSMD1, PSMD11, PSMD12, PSMD13, PSMD14, PSMD2, PSMD3, PSMD4, PSMD6, SNCA, TGFBI, TRAF4, UBC, UCHL1, UFD1, USP46, ZFAND2A |
| Cluster 2 (bright green) | ADCY9, CGB5, CGB8, F2RL2, GNB4, GNG2, HRH1, KCNJ2, PRKCH |
| Cluster 3 (purple/blue) | CALU, NSFL1C, PLAA, SEL1L3, UACA, UBE4B, UBQLN1, UBXN4, VCP |
| Cluster 4 (light green/mint) | H3BN98\_HUMAN, MRNIP, NAP1L2, PELO, RPL22L1, RPL39, RPS12, SRPRA, VEZT |
| Cluster 5 (orange/peach) | ACO2, DAP3, MSRB1, RBM48, SOD2, TICAM2, UXS1 |
| Cluster 6 (yellow) | AEBP2, CBX4, CD3EAP, GREB1, H2AC6, SCML2, YEATS4 |
| Cluster 7 (teal/light cyan) | CHAC1, GPX3, GSR, MGST1, MTHFD2, PDHX |
| Cluster 8 (beige/light tan) | CCR3, CXCL1, CXCL2, CXCL3, CXCL6, CXCR4 |
| Cluster 9 (grey/silver) | CSF1R, EGF, IL21R, IL6R, LOX, TGFA |
| Cluster 10 (purple/lavender) | BHLHE41, CREM, CRY2, PER1, PER2 |
| Cluster 11 (light blue/periwinkle) | AKR1C3, BLVRB, CYP19A1, SRD5A1, UGT1A8 |
| Cluster 12 (brown/tan) | ARHGAP32, ARHGEF16, MAP1A, MYH14, RHOB |
Supplementary Table 13: Clusters ≥ 5 protein-protein interactions identified between differentially expressed gene-encoded proteins upon the HK2KD IN859 STRING network.
| HK2KD IN2045 STRING protein-protein interaction cluster | Comprising differentially expressed gene-encoded proteins |
| --- | --- |
| Cluster 1 (pink/red) | H3BN98\_HUMAN, RPL31, RPL36, RPL36A, RPL37, RPL37A, RPL38, RPL39, RPL41, RPS21, RPS25, RPS27, RPS28, RPS29, SEC61G, SRPRA, UXS1 |
| Cluster 2 (bright green) | NDUFA1, NDUFA13, NDUFA3, NDUFB1, NDUFC1, SNCA, TGFBI, UQCRHL, UQCRQ |
| Cluster 3 (purple/blue) | CSF1R, FGF2, MET, NTRK2, SDC4, TIMP1 |
Supplementary Table 14: Clusters ≥ 5 protein-protein interactions identified between differentially expressed gene-encoded proteins upon the HK2KD IN2045 STRING network.
